# IRP1 deficiency alters mitochondrial metabolism and protects against metabolic syndrome pathologies

**DOI:** 10.1172/jci.insight.183247

**Published:** 2026-01-06

**Authors:** Wen Gu, Nicole Wilkinson, Carine Fillebeen, Darren M. Blackburn, Korin Sahinyan, Eric Bonneil, Tao Zhao, Zhi Luo, Vahab D. Soleimani, Vincent Richard, Christoph H. Borchers, Albert Koulman, Benjamin Jenkins, Bernhard Michalke, Hans Zischka, Judith Sailer, Vivek Venkataramani, Othon Iliopoulos, Gary Sweeney, Kostas Pantopoulos

**Affiliations:** 1 Lady Davis Institute for Medical Research, Jewish General Hospital and Department of Medicine, McGill University, Montreal, Quebec, Canada.; 2 Department of Biochemistry, Microbiology & Immunology, University of Ottawa, Ottawa, Ontario, Canada.; 3 Institute for Research in Immunology and Cancer, University of Montreal, Montreal, Quebec, Canada.; 4 Hubei Hongshan Laboratory, Fishery College, Huazhong Agricultural University, Wuhan, China.; 5 Institute of Metabolic Science-Metabolic Research Laboratories, University of Cambridge, Cambridge, United Kingdom.; 6 Helmholtz Zentrum München GmbH – German Research Center for Environmental Health, Research Unit Analytical BioGeoChemistry, Neuherberg, Germany.; 7 Environmental Hygiene, Technical University Munich, Munich, Germany.; 8 Helmholtz Zentrum München GmbH – German Research Center for Environmental Health, Institute of Molecular Toxicology and Pharmacology, Neuherberg, Germany.; 9 Department of Internal Medicine II, Medical Oncology, and Department of Neurosurgery, University Hospital Würzburg; Bavarian Cancer Research Center (BZKF), National Center for Tumor Diseases (NCT) WERA, Germany.; 10 Department of Medicine, Hematology-Oncology Unit, Massachusetts General Hospital and the Massachusetts General Hospital Cancer Center, Boston, Massachusetts, USA.; 11 Department of Biology, York University, Toronto, Ontario, Canada.

**Keywords:** Hepatology, Metabolism, Diabetes, Glucose metabolism, Proteomics

## Abstract

Iron regulatory protein 1 (IRP1) is a posttranscriptional regulator of cellular iron metabolism. In mice, loss of IRP1 causes polycythemia through translational de-repression of HIF2α mRNA, which increases renal erythropoietin production. Here, we show that *Irp1^–/–^* mice develop fasting hypoglycemia and are protected against high-fat diet–induced hyperglycemia and hepatic steatosis. Discovery-based proteomics of *Irp1^–/–^* livers revealed a mitochondrial dysfunction signature. Seahorse flux analysis in primary hepatocytes and differentiated skeletal muscle myotubes confirmed impaired respiratory capacity, with a shift from oxidative phosphorylation to glycolytic ATP production. This metabolic rewiring was associated with enhanced insulin sensitivity and increased glucose uptake in skeletal muscle. Under metabolic stress, IRP1 deficiency altered the redox balance of mitochondrial iron, resulting in inefficient energy production and accumulation of amino acids and metabolites in skeletal muscles, rendering them unavailable for hepatic gluconeogenesis. These findings identify IRP1 as a critical regulator of systemic energy homeostasis.

## Abstract 2

Mice lacking iron regulatory protein 1 (IRP1) show altered energy metabolism and are protected against metabolic syndrome pathologies

## Introduction

Iron regulatory proteins IRP1 and IRP2 coordinately control the expression of mRNAs containing iron responsive elements (IREs). These include *Tfrc*, *Fth/Ftl,* and *Slc40α1* mRNAs, which encode proteins of iron uptake (transferrin receptor 1), storage (ferritin), and efflux (ferroportin), respectively (1). In iron-starved cells, IRE/IRP interactions protect *Tfrc* mRNA against degradation and inhibit translation of *Fth/Ftl* and *Slc40α1* mRNAs, in a homeostatic response to secure adequate iron supply. Conversely, in iron-replete cells, IRP1 is converted to cytosolic aconitase at the expense of its RNA-binding activity, while IRP2 undergoes proteasomal degradation. Inactivation of IRPs permits the decay of *Tfrc* mRNA and the de novo synthesis of ferritin and ferroportin to prevent detrimental iron overload.

IRP1 and IRP2 exhibit extensive sequence homology but only partial functional redundancy (2). Although global or tissue-specific disruption of both IRPs has yielded lethal phenotypes (3–5), single *Irp1^–/–^* and *Irp2^–/–^* mice are viable and have distinct pathophysiological features. Thus, *Irp1^–/–^* mice develop polycythemia due to translational de-repression of the IRE-containing mRNA encoding HIF2α, which in turn stimulates erythropoiesis via transcriptional induction of erythropoietin in the kidney (6–8). On the other hand, *Irp2^–/–^* mice manifest microcytic anemia, mild iron overload in the liver and duodenum, erythropoietic protoporphyria, diabetes, and late-onset neurodegeneration (9–11).

Herein, we show that *Irp1^–/–^* mice are protected against hyperglycemia, insulin resistance, and liver steatosis. These are common clinical manifestations of metabolic syndrome, a pathological state defined by the combined presentation of at least 3 of the following conditions: abdominal obesity, hyperglycemia due to insulin resistance, dyslipidemia, and hypertension. Metabolic syndrome has a prevalence of approximately 25% in the adult US population and increases the risk for type 2 diabetes, cardiovascular and liver disease, and all-cause mortality (12). We demonstrate here that IRP1 deficiency causes mitochondrial dysfunction and metabolic rewiring that alters energy homeostasis. Our data unveil an unexpected role of IRP1 in the control of intermediary metabolism.

## Results

### Irp1^–/–^ mice are protected against high fat diet–induced hyperglycemia.

**Figure 1:**
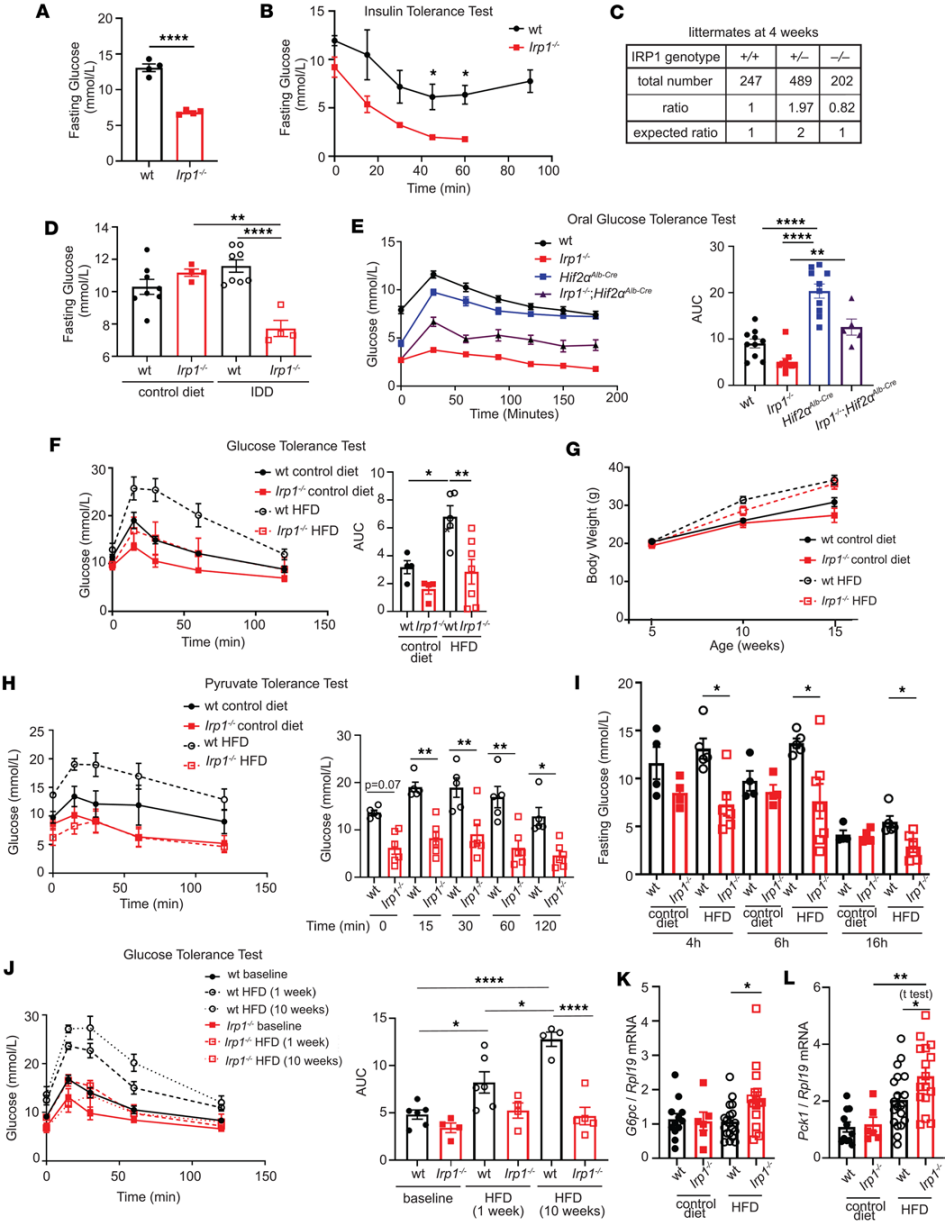
Young *Irp1^–/–^* mice are hypoglycemic, and adult animals do not develop high fat diet–induced hyperglycemia. In **A**, **B**, **D**, and **F**–**L**, male *Irp1^–/–^* mice and WT littermates (*n* = 4–8 per experimental group) were analyzed at weaning (age of 5 weeks) or following dietary interventions initiated immediately after weaning (10 weeks, unless otherwise indicated). In **E**, male *Irp1^–/–-^,* WT, *Irp1^–/–^* *Hif2α^Alb-Cre^*, and *Hif2α^Alb-Cre^* mice (*n* = 5–10 per experimental group) were analyzed at weaning. (**A**) Fasting (5 hours) blood glucose levels and (**B**) insulin tolerance test at weaning, after 4 hours’ fasting. (**C**) Genotyping of all *Irp1^+/+^*, *Irp1^+/–^*, and *Irp1^–/–^* littermates generated throughout this study. (**D**) Fasting (5 hours) blood glucose levels in littermate mice fed a control or an iron-deficient diet (IDD). (**E**) Oral GTT after 16 hours’ fasting. (**F**) GTT after 5 hours’ fasting; (**G**) time-dependent changes in body weight of mice fed a control or a high-fat diet (HFD); and (**H**) pyruvate tolerance test after 6 hours’ fasting. (**I**) Blood glucose levels in littermate mice fed control or HFD following fasting for 4, 6, or 16 hours. (**J**) GTT in littermate mice after 4 hours’ fasting at baseline, and after feeding HFD for 1 week and 10 weeks. (**K** and **L**) qPCR analysis of liver *G6pc* (**K**) and *Pck1* (**L**) mRNA expression in non-fasted littermate mice fed control or HFD for 12 weeks. The right panels in **E**, **F**, and **J** show AUC; in **H** pairwise comparisons of data from HFD-fed WT and *Irp1^–/–^* mice at various time points. Quantitative data are presented as the mean ± SEM. Statistical analysis was performed with 2-way ANOVA except E, which was 1-way ANOVA, with Tukey’s multiple-comparison test; comparisons between 2 groups were done with 2-tailed Student’s *t* test. *P < 0.05, **P < 0.01, ****P < 0.0001.

Young *Irp1^–/–^* mice develop erythrocytosis due to induction of the HIF2α/erythropoietin axis (6–8), and gradually recover after the age of 8–10 weeks, where HIF2α overexpression is mitigated (6). Prompted by evidence that HIF2α also controls insulin signaling in the liver via transcriptional induction of insulin receptor substrate 2 (IRS2) (13, 14), we analyzed metabolic functions in these animals. At the age of 5 weeks, *Irp1^–/–^* mice exhibited profound fasting hypoglycemia (Figure 1A), while the phenotype of heterozygous *Irp1^+/–^* animals was indistinguishable from WT controls (data not shown). Insulin tolerance testing (ITT) caused a sharper early drop in blood glucose levels of *Irp1^–/–^* mice (Figure 1B), indicating increased insulin sensitivity. Moreover, *Irp1^–/–^* mice could not initiate a gradual return of blood glucose levels to baseline within 45–60 minutes after an insulin injection; instead, they developed hypoglycemic seizures and had to be treated for recovery. In line with the severe fasting hypoglycemia, we observed 18% reduced survival of *Irp1^–/–^* pups at the age of 4 weeks (Figure 1C), which was more pronounced in male (24%) versus female (11%) animals (Supplemental Figure 1A; supplemental material available online with this article; https://doi.org/10.1172/jci.insight.183247DS1). Notably, blood glucose levels were normalized in older *Irp1^–/–^* mice (Figure 1D, control diet), but the age-dependent correction of hypoglycemia was abolished when animals were fed an iron-deficient diet (IDD) (Figure 1D). These data suggest that IRP1 deficiency has an impact on metabolism and are consistent with HIF2α involvement. However, while *Irp1^–/–^* mice exhibited rapid glucose clearance in an oral glucose tolerance test (GTT), hepatocyte-specific ablation of HIF2α only partially corrected this phenotype in *Irp1^–/–^* *Hif2α^Alb-Cre^* mice (Figure 1E).

We then analyzed the responses of *Irp1^–/–^* mice and WT littermates to a high-fat diet (HFD). Young (5–6 weeks old) male animals were fed an HFD or control diet for 10 weeks, and subsequently received a GTT. As expected, WT mice on an HFD developed hyperglycemia and glucose intolerance, whereas *Irp1^–/–^* mice maintained physiological blood glucose levels and glucose clearance despite HFD feeding (Figure 1F). The observed metabolic differences were independent of serum insulin levels (Supplemental Figure 1B) or weight gain (Figure 1G), which were similar in HFD-fed WT and mutant animals. No significant differences in blood pressure were noted among the experimental groups (Supplemental Figure 1, C and D). We noted that the hypoglycemic phenotype persisted in both male and female young *Irp1^–/–^* mice (data not shown). However, female *Irp1^–/–^* mice appeared less protected than males against HFD-induced hyperglycemia (Supplemental Figure 1E). Thus, we focused on male animals for mechanistic studies.

Pyruvate tolerance testing revealed that *Irp1^–/–^* mice exhibit a modest impairment in pyruvate-induced hepatic gluconeogenesis (Figure 1H). This was corroborated by decreased glucose levels in these animals after16 hours of fasting (Figure 1I), under conditions where glycogen stores are depleted and glucose supply largely depends on hepatic gluconeogenesis (15). Furthermore, *Irp1^–/–^* mice on an HFD exhibited lower blood glucose levels after 4 and 6 hours of fasting compared with WT controls, indicating reduced hepatic glycogen storage (Figure 1I). This hypoglycemic phenotype was also recapitulated in a longitudinal experiment, in which *Irp1^–/–^* mice and WT littermates were subjected to GTT at baseline and at 1 and 10 weeks after initiation of HFD intake. Unlike WT animals, which developed increased glucose intolerance with prolonged HFD feeding, the *Irp1^–/–^* mice exhibited minimal defects in glucose clearance mostly after 1 week of HFD intake but not at the endpoint (10 weeks), suggesting metabolic adaptation and reprogramming (Figure 1J). To gain mechanistic insights, we analyzed hepatic expression of genes involved in gluconeogenesis (*G6pc*, *Pck1*, *Fbp1*) in WT and *Irp1^–/–^*mice fed a control diet or an HFD. *G6pc* (Figure 1K) and *Pck1* (Figure 1L) were significantly upregulated in *Irp1^–/–^* versus WT animals on the HFD, and a similar trend appeared for *Fbp1* (Supplemental Figure 1F), despite the overall impaired gluconeogenic capacity. The expression of HIF2α-inducible *Irs2* (but not *Irs1*) tended to be higher in *Irp1^–/–^* mice on the HFD (Supplemental Figure 1G). This indicates potentially increased insulin sensitivity and is again consistent with a role of HIF2α. The induction of gluconeogenic genes despite increased insulin sensitivity suggests that *Irp1^–/–^* mice are in an energy-deprived state.

Metabolic cage analysis of WT and *Irp1^–/–^* mice on the HFD showed no significant differences among the genotypes on food or water intake, oxygen consumption, CO_2_ production, respiratory exchange rate, or heat, while *Irp1^–/–^* mice tended to exhibit slightly increased movement activities (Supplemental Figure 2).

### Irp1^–/–^ mice are protected against HFD-induced liver steatosis.

**Figure 2:**
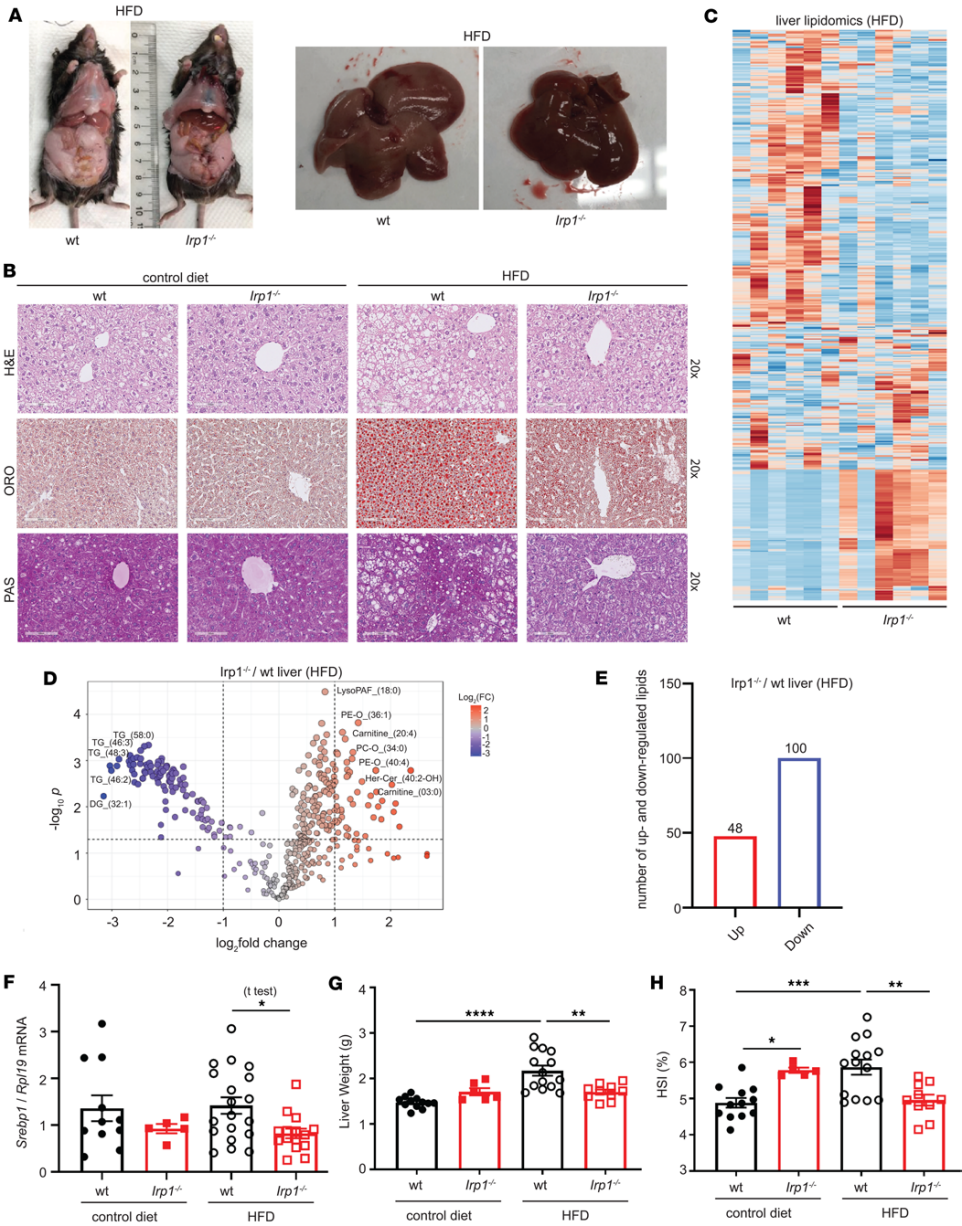
*Irp1^–/–^* mice are protected against high fat diet–induced liver steatosis. For 10 weeks, 5-week-old male *Irp1^–/–^* mice and WT littermates (*n* = 6–19 per experimental group) were provided a control or high-fat diet (HFD) for 10 weeks. At the endpoint, the mice were euthanized, and livers were dissected for histological and biochemical analysis. (**A**) Images of representative mice and dissected livers after euthanasia. (**B**) Histopathological analysis of liver sections by staining with H&E for tissue architecture (top), Oil Red O (ORO) for neutral fats and lipids (middle), and periodic acid–Schiff (PAS) for glycogen (bottom); original magnification, ×20. (**C**) Heatmap of liver lipidomic changes between WT and *Irp1^–/–^* mice on HFD (*n* = 6 mice from each genotype). (**D**) Volcano plot of differentially expressed lipids in the liver of *Irp1^–/–^* and WT mice on HFD; *P* value threshold was set to 0.05 and fold change to 2. (**E**) Number of up- and down-regulated lipids in the liver of *Irp1^–/–^* versus WT mice on HFD. (**F**) qPCR analysis of liver *Srebp1* mRNA expression. (**G**) Liver weight. (**H**) Hepatosomatic index (HSI). Quantitative data are presented as the mean ± SEM. Statistical analysis was performed with 2-way ANOVA with Tukey’s multiple-comparison test; comparisons between 2 groups were done with 2-tailed Student’s *t* test. *P < 0.05, **P < 0.01, ***P < 0.001, ****P < 0.0001.

After 10 weeks of HFD feeding, livers from WT mice became pale, indicative of steatosis; by contrast, livers from *Irp1^–/–^* littermates retained their physiological color, suggesting protection against steatosis (Figure 2A). Histopathological analysis using H&E or Oil Red O staining confirmed that liver sections from *Irp1^–/–^* mice had significantly reduced fat content, without large visible lipid droplets (Figure 2B), indicating that these animals were spared from HFD-induced hepatic steatosis.

The histological findings were corroborated by large-scale lipidomics profiling; the heatmap in Figure 2C reveals dramatic alterations in the liver lipidome of *Irp1^–/–^* mice. Quantified liver lipidomics data are provided in Supplemental Data Set 2. A total of 100 lipid species were upregulated and 48 downregulated in the liver of *Irp1^–/–^* versus WT mice (Figure 2, D and E). Upregulated lipids included phosphatidylethanolamine plasmalogens (PE-O_36:1 and PE-O_40:4), arachidonoyl-L-carnitine (carnitine_20:4), and phosphatidylcholine plasmalogens (PC-O_34:0 and PCO_31:0). Downregulated lipids included triacylglycerols (TG_58:0, TG_46:3, TG_48:3, and TG_46:2) and diacylglycerols (DG_32:1), which may indicate decreased hepatic lipogenesis in *Irp1^–/–^* mice. Supporting evidence is provided by the significantly reduced expression of *Srebp1* mRNA (Figure 2F) encoding a key transcriptional activator of de novo hepatic lipogenesis (16).

Consistent with these findings, HFD feeding increased liver weight and the hepatosomatic index (liver weight expressed as a percentage of total body weight) in WT mice only (Figure 2, G and H). In contrast, the hepatosomatic index decreased in HFD-fed *Irp1^–/–^* animals. Periodic acid–Schiff (PAS) staining suggested that livers from *Irp1^–/–^* mice on the HFD also had reduced glycogen content (Figure 2B), in line with the relative hypoglycemia that these animals developed after 4 and 6 hours of fasting (Figure 1I).

Serum biochemistry showed that an HFD increased total cholesterol in both WT and mutant mice (Supplemental Figure 3A), while triglyceride levels tended to be lower in *Irp1^–/–^* mice independent of diet (Supplemental Figure 3B). Despite elevated HDL cholesterol in both genotypes after HFD intake (Supplemental Figure 3C), non-HDL cholesterol levels appeared lower in *Irp1^–/–^* animals (Supplemental Figure 3D), and total cholesterol/HDL ratios were not affected (Supplemental Figure 3E). Values for serum iron, total iron-binding capacity (TIBC), transferrin saturation, and liver *Hamp* mRNA did not differ among all experimental groups (Supplemental Figure 3, F–I), whereas serum ferritin was elevated in *Irp1^–/–^* mice (Supplemental Figure 3J), possibly due to translational de-repression under IRP1 deficiency. Complete blood count showed a normal red blood cell number and even reduced hematocrit in *Irp1^–/–^* mice after the 10-week HFD feeding period (Supplemental Table 1), validating the correction of polycythemia with age (6). Along these lines, liver *Epo* mRNA and serum Epo levels were similar in all groups (Supplemental Figure 3, K and L). Nevertheless, splenomegaly persisted in the *Irp1^–/–^* animals (Supplemental Figure 3, M–O), indicating extramedullary hematopoiesis. This was not associated with altered splenic iron content (Supplemental Figure 3P).

### IRP1 deficiency causes mitochondrial dysfunction and a shift of energy metabolism from oxidative phosphorylation to glycolysis.

**Figure 3:**
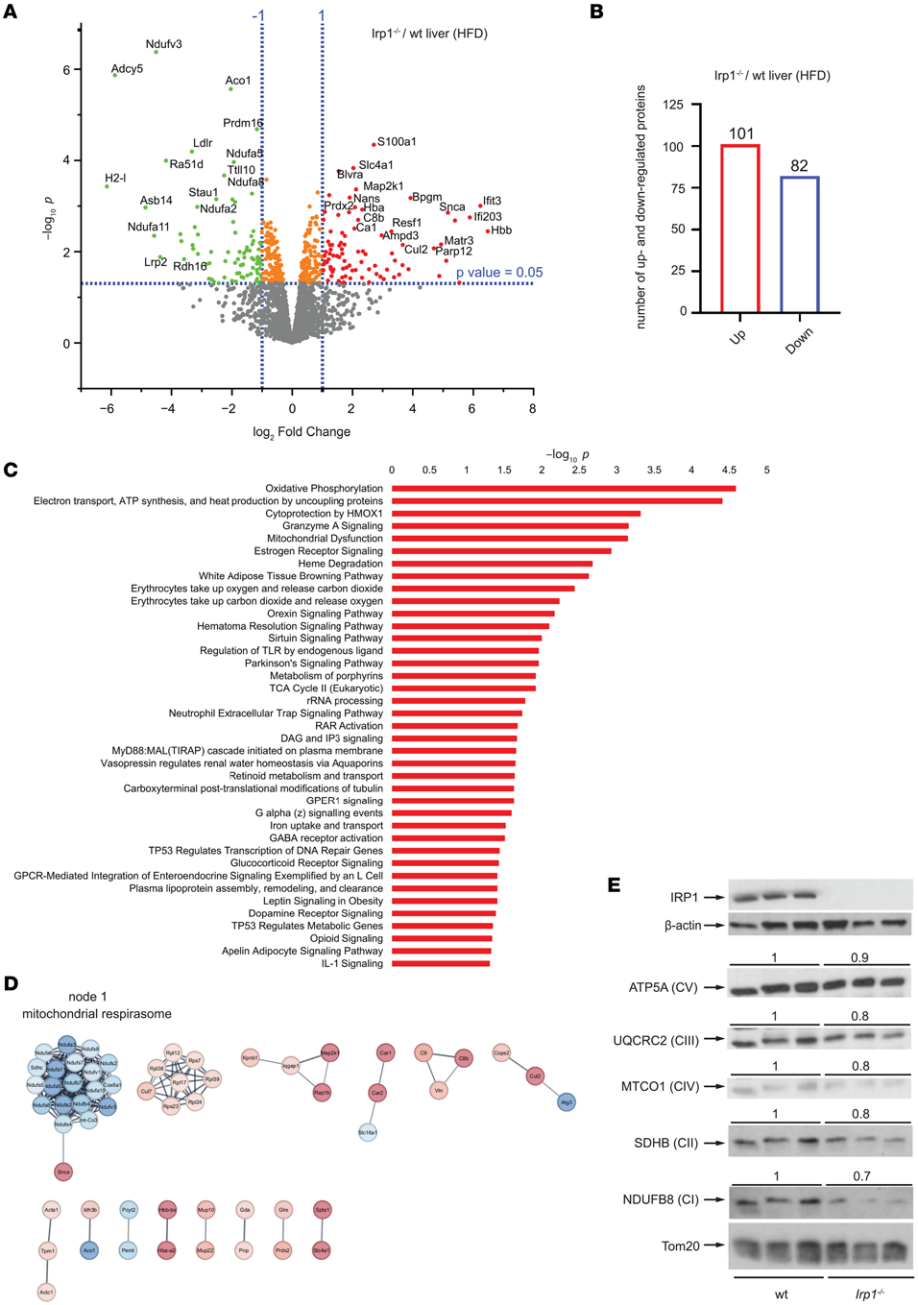
Altered mitochondrial proteome in the liver of *Irp1^–/–^* mice. For10 weeks, 5-week-old male *Irp1^–/–^* mice and WT littermates (*n* = 4 per experimental group) were provided a high-fat diet (HFD). At the endpoint, the mice were euthanized, and livers were dissected for proteomics analysis. (**A**) Volcano plot of differentially expressed proteins (*Irp1^–/–^* versus WT); *P* value threshold was set to 0.05 and fold change to 2. (**B**) Number of up- and down-regulated proteins in the liver of *Irp1^–/–^* versus WT mice. (**C**) Pathway enrichment analysis generated by the QIAGEN IPA software. (**D**) STRING networks based on all differentially expressed proteins show that the major enrichment in GO terms and KEGG pathways is linked to mitochondrial respirasome. (**E**) Western blot analysis of mitochondrial respiratory chain proteins corresponding to complexes I, II, III, IV, and V using whole liver extracts from 3 representative mice in each group. The membranes were also probed with antibodies against IRP1, β-actin (cytosolic loading control), and Tom20 (mitochondrial loading control). Blots were quantified by densitometry relative to Tom20, and average values are shown on top. Ratios of respiratory chain proteins to Tom20 from WT mice are set as 1.

An unbiased proteomics approach was utilized to elucidate pathways by which IRP1 deficiency affects metabolic functions. To this end, liver samples from WT and *Irp1^–/–^* mice on HFD (*n* = 4 from each group) were subjected to liquid chromatography–tandem mass spectrometry (LC-MS/MS) analysis. After filtering, 2,300 proteins on average were identified in each sample. Principal component analysis showed good separation between samples from the 2 genotypes (Supplemental Figure 4A), and Pearson correlation analysis showed that the replicates correlated well (Supplemental Figure 4B). Quantified liver proteomics data are provided in Supplemental Data Set 1. In *Irp1^–/–^* versus WT livers, 101 proteins were upregulated and 82 downregulated (Figure 3, A and B). Upregulated proteins included S100a1, Slc4a1, and Blvra. Prominent downregulated proteins were Nduf3, Adcy5, Prdm16, and IRP1 (Aco1), which served as a control. Enrichment analysis with IPA software (QIAGEN) identified mitochondrial pathways (oxidative phosphorylation, electron transport, ATP synthesis) as most affected by IRP1 deficiency in the liver of HFD-fed mice (Figure 3C). The top hits in Gene Ontology (GO) hierarchy analysis were catalytic activity (GO Molecular Function), small molecule metabolic process (GO Biological Process), and cytoplasm and mitochondrion (GO Cellular Component); the top hits in Kyoto Encyclopedia of Genes and Genomes (KEGG) and Reactome pathway analysis were metabolic pathways and metabolism, respectively (Supplemental Figure 5). These data uncovered metabolic reprograming in the liver of *Irp1^–/–^* mice that involved mitochondria. Mitochondrial dysfunction was also highlighted in STRING functional association networks (Figure 3D) and validated by Western blot analysis for electron transport chain complexes, showing reduced expression, especially of proteins representing complexes I and II, but also III and IV in the liver of *Irp1^–/–^* mice (Figure 3E). This was not accompanied by any significant changes in expression of markers of mitochondrial biogenesis (*Pgc1α* mRNA), fission (*Fis1* and *Drp1* mRNAs), or fusion (*Mfn1* and *Mfn2* mRNAs) (17) (Supplemental Figure 6, A–E).

**Figure 4:**
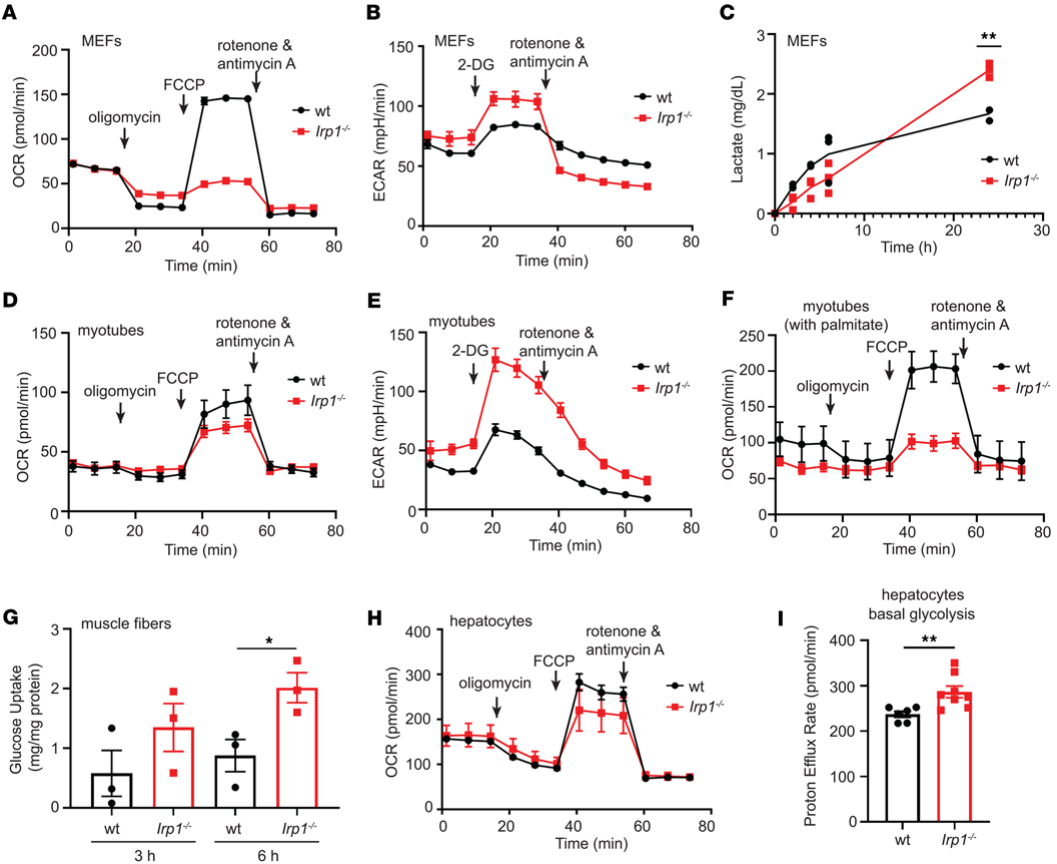
IRP1 deficiency causes mitochondrial dysfunction and a switch from oxidative energy metabolism to aerobic glycolysis. Mouse embryonic fibroblasts (MEFs), primary hepatocytes, and differentiated myotubes from *Irp1^–/–^* and WT mice were analyzed for mitochondrial function with the Seahorse assay. The time for addition of oligomycin, Carbonyl cyanide p-(trifluoromethoxy) phenylhydrazone, and rotenone/antimycin A or 2-deoxy-glucose (2-DG) is indicated by arrows. (**A**) Oxygen consumption rate (OCR) in *Irp1^–/–^* and WT MEFs. (**B**) Extracellular acidification rate (ECAR) in *Irp1^–/–^* and WT MEFs. (**C**) Lactate production by *Irp1^–/–^* and WT MEFs. (**D**) OCR in *Irp1^–/–^* and WT differentiated myotubes. (**E**) ECAR in *Irp1^–/–^* and WT differentiated myotubes. (**F**) OCR with palmitate as substrate in *Irp1^–/–^* and WT differentiated myotubes. (**G**) Glucose uptake assay in muscle fibers from *Irp1^–/–^* and WT mice. (**H**) OCR in *Irp1^–/–^* and WT primary hepatocytes. (**I**) Proton efflux rate indicating basal glycolysis in *Irp1^–/–^* and WT primary hepatocytes. Data in **G** and **I** are presented as mean ± SEM. Statistical analysis performed with 2-way ANOVA with Tukey’s multiple-comparison test; comparisons between 2 groups were done with 2-tailed Student’s *t* test. *P < 0.05, **P < 0.01.

Diminished expression of electron transport chain complexes has previously been reported in *Irp1^–/–^* murine embryonic fibroblasts (MEFs) (18). To assess functional implications, we analyzed mitochondrial oxygen consumption rates in *Irp1^–/–^* MEFs with the Seahorse assay. IRP1 deficiency significantly impaired basal, ATP-linked, and maximal respiration, as well as spare capacity (Figure 4A and Supplemental Figure 7, A–D). This was associated with increased glycolytic activity and lactate production (Figure 4, B and C); both basal and compensatory glycolysis were induced (Supplemental Figure 7, E and F). Similar results were obtained with differentiated primary skeletal muscle myotubes from *Irp1^–/–^* mice (Figure 4, D and E, and Supplemental Figure 7, G–L), which also exhibited defective mitochondrial respiration when palmitate was used as substrate (Figure 4F and Supplemental Figure 7, M–P). Along these lines, muscle fibers from *Irp1^–/–^* mice had increased glucose uptake capacity (Figure 4G). IRP1 deficiency did not affect muscle stem cell differentiation to myotubes (Supplemental Figure 8A) but increased proliferation rates of myoblasts (Supplemental Figure 8B) and MEFs (Supplemental Figure 8C). In line with the liver proteomics data, primary hepatocytes from *Irp1^–/–^* mice likewise exhibited impaired mitochondrial respiration (Figure 4H and Supplemental Figure 7, Q–T) and increased basal glycolysis (Figure 4I). The effects in hepatocytes appeared milder compared with MEFs and myotubes, and statistically significant differences among the genotypes were only noted for spare capacity (Supplemental Figure 7T) and basal (Figure 4I) but not compensatory glycolysis (not shown). Taken together, the above data suggest that IRP1 deficiency triggers mitochondrial dysfunction and a shift to aerobic glycolysis for energy production.

### IRP1 deficiency alters mitochondrial redox speciation of iron in the liver and skeletal muscles.

**Figure 5:**
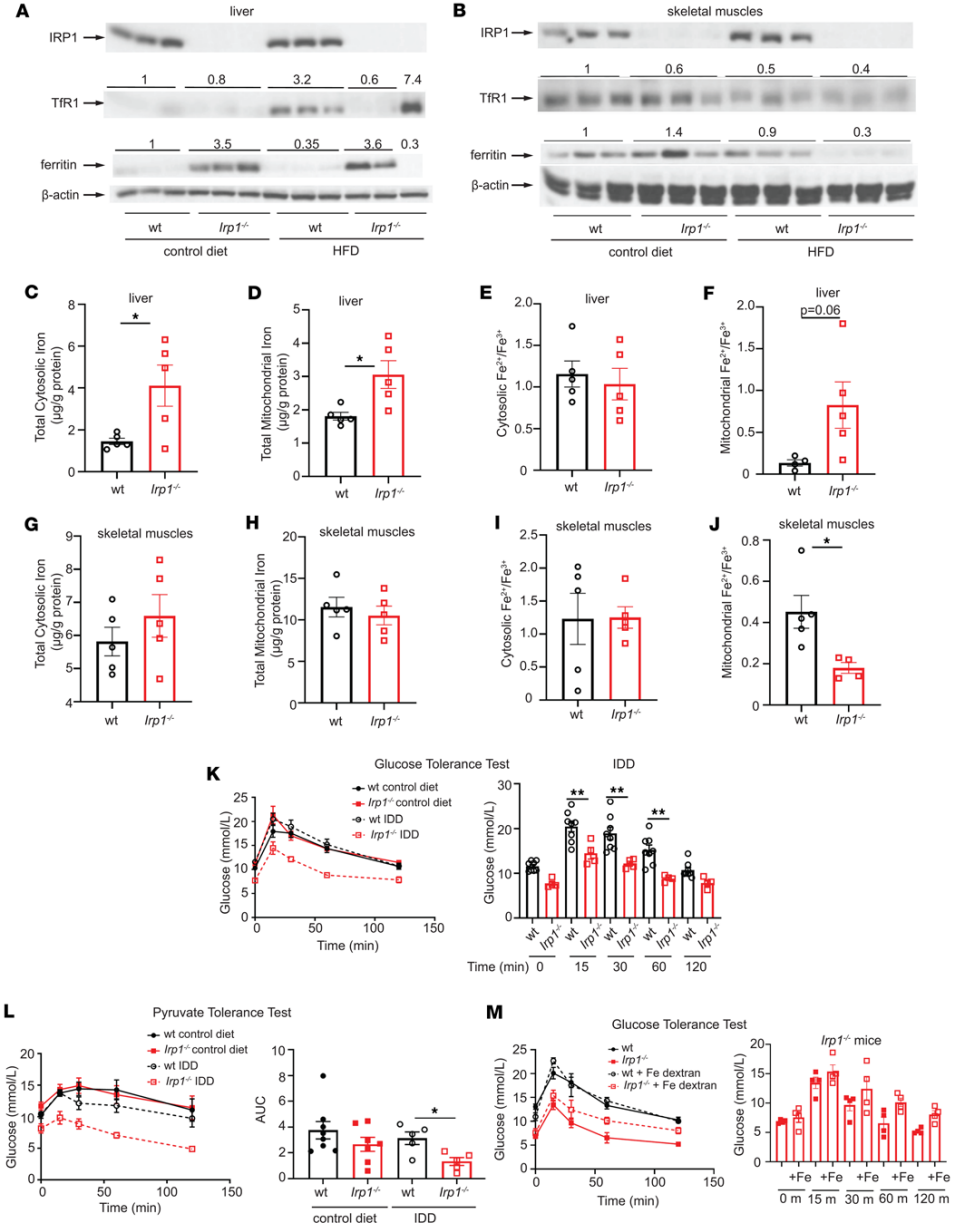
Evidence that mitochondrial dysfunction in IRP1-deficient cells is caused by impaired mitochondrial redox speciation of iron. (**A**–**J**) Male WT and *Irp1^–/–^* mice (5 weeks old; *n* = 5–8 per experimental group) received control diet or HFD for 10 weeks. Liver and quadriceps skeletal muscle tissue samples were isolated for biochemical studies at endpoint. Cytosolic and mitochondrial fractions were prepared and used for quantification of total iron and iron speciation analysis. (**A** and **B**) Western blot analysis of IRP1, TfR1, ferritin, and β-actin in liver (**A**) and skeletal muscles (**B**). (**C**–**F**) Total cytosolic iron (**C**), total mitochondrial iron (**D**), cytosolic Fe^2+^/Fe^3+^ ratios (**E**), and mitochondrial Fe^2+^/Fe^3+^ ratios (**F**) in liver. (**G**–**J**) Total cytosolic iron (**G**), total mitochondrial iron (**H**), cytosolic Fe^2+^/Fe^3+^ ratios (**I**), and mitochondrial Fe^2+^/Fe^3+^ ratios (**J**) in skeletal muscles. (**K**–**M**) For 10 weeks, 5-week-old male WT and *Irp1^–/–^* mice (*n* = 5–7 per experimental group) received control diet or IDD. (**K**) GTT after 5 hours’ fasting. Right: pairwise comparisons of data from WT and *Irp1^–/–^* mice on IDD at various time points. (**L**) Pyruvate tolerance test after 6 hours’ fasting. Right: AUC. (**M**) GTT in 5-week-old male *Irp1^–/–^* mice after 5 hours’ fasting. Right: pairwise comparisons of data from *Irp1^–/–^* mice with or without iron dextran injections at various time points. In **A** and **B**, Western blots for TfR1 and ferritin quantified by densitometry relative to β-actin; average values shown on top (due to experimental variability, quantification of “outlier” sample shown separately). TfR1/β-actin and ferritin/β-actin ratios from WT mice set as 1. In **C**–**J**, iron measurements normalized to protein content and expressed as mean ± SD. Data in **C**–**M** shown as mean ± SEM. Statistical analysis performed with 2-way ANOVA with Tukey’s multiple-comparison test; 2-tailed Student’s *t* test for comparisons between 2 groups. *P < 0.05, **P < 0.01.

In mouse (19) and rat (20) models of liver steatosis, HFD intake elicited a phenotype of low hepatic iron characterized by induction of transferrin receptor 1 (TfR1), the major iron uptake protein. In agreement with the earlier findings, we noted that WT mice on the HFD manifested increased TfR1 levels in the liver (Figure 5A). TfR1 expression was reduced, and ferritin was upregulated in the liver of *Irp1^–/–^* versus WT mice on the control diet, presumably due to IRP1 deficiency. These responses were preserved after HFD feeding; however, a small number (2 out of 9) of the analyzed livers from *Irp1^–/–^* animals exhibited high TfR1 and low ferritin levels (only 3 representative samples from each condition are shown in the Western blot).

On the other hand, HFD intake neither induced TfR1 nor suppressed ferritin in skeletal muscles of WT mice (Figure 5B), indicating relatively normal iron balance in this tissue. Ferritin was paradoxically suppressed in skeletal muscles of HFD-fed *Irp1^–/–^* mice and TfR1 levels appeared slightly reduced, possibly due to IRP1 deficiency. These data suggest that iron homeostasis in the liver and skeletal muscles is controlled by distinct tissue-specific pathways in response to an HFD, with or without IRP1 deficiency.

To shed light on these pathways and dissect the role of IRP1, we profiled ferrous (Fe^2+^) and ferric (Fe^3+^) iron content in the cytosolic and mitochondrial fractions from the liver and skeletal muscles of WT and *Irp1^–/–^* mice on an HFD, using cation exchange chromatography coupled with inductively coupled plasma mass spectrometry (SCX-ICP-MS). The Fe^2+^/Fe^3+^ ratio was calculated as an indicator of redox-active, labile iron pools. The purity of subcellular fractions was assessed by Western blotting for cytosolic IRP1 and mitochondrial Tom20 (Supplemental Figure 9, A and B).

Total (Fe^2+^ and Fe^3+^) iron levels were significantly higher in the liver cytosolic (~2.8-fold) and mitochondrial (~1.7-fold) fractions of *Irp1^–/–^* mice compared with WT controls (Figure 5, C and D). Separate measurements of Fe^2+^ and Fe^3+^ are shown in Supplemental Figure 9, C and D. Although cytosolic Fe^2+^/Fe^3+^ ratios were similar between genotypes (Figure 5E), the mitochondrial Fe^2+^/Fe^3+^ ratio was elevated by approximately 6-fold in *Irp1^–/–^* livers (Figure 5F), suggesting a relative accumulation of reduced, potentially unutilized, and redox-active Fe^2+^ in IRP1-deficient liver mitochondria.

In skeletal muscles, total cytosolic and mitochondrial iron (Figure 5, G and H), Fe^2+^ levels (Supplemental Figure 9E), Fe^3+^ (Supplemental Figure 9F) levels, and cytosolic Fe^2+^/Fe^3+^ ratios (Figure 5I) were comparable across genotypes. However, mitochondrial Fe^2+^/Fe^3+^ ratios were significantly decreased approximately 2.5-fold in *Irp1^–/–^* skeletal muscles (Figure 5J), indicating a relative oxidation of the mitochondrial iron pool in the absence of IRP1. Collectively, these iron speciation data suggest that IRP1 deficiency impairs mitochondrial iron homeostasis in both the liver and skeletal muscles under metabolic stress induced by HFD, while cytosolic iron buffering remains unaffected.

Contrary to data previously reported in MEFs (18), IRP1 deficiency (or HFD intake) did not significantly affect expression of the Fe-S cluster biogenesis proteins frataxin and IscU in the liver and skeletal muscles (Supplemental Figure 9, G and H). Additionally, it did not affect expression of the mitochondrial iron transporter mitoferrin-1 (Mfrn1; Supplemental Figure 9, G and H). Notably, *Irp1^–/–^* mice on an HFD had impaired mitochondrial aconitase activity in the heart (Supplemental Figure 9I). Considering that catalytic aconitase activity requires a 4Fe-4S cluster in the active site of the enzyme, these data suggest that HFD-induced metabolic stress causes functional inactivation of Fe-S clusters in IRP1-deficient mitochondria.

Our findings suggest that the metabolic phenotype of *Irp1^–/–^* mice on an HFD may be linked to defective mitochondrial iron and redox homeostasis. Based on this, we hypothesized that dietary iron restriction might elicit metabolic responses in these animals analogous to those induced by HFD. Indeed, adult *Irp1^–/–^* mice fed an IDD not only maintained fasting hypoglycemia (Figure 1D), but also exhibited improved glucose clearance (Figure 5K) and blunted gluconeogenesis (Figure 5L). Notably, injection with iron dextran did not affect glucose clearance in young (5 weeks old) WT mice but substantially impaired it in hypoglycemic *Irp1^–/–^* littermates (Figure 5M), underlying the critical role of iron in glucose metabolism. These data demonstrate that dietary iron deficiency mimics effects of metabolic stress imposed by an HFD on glucose homeostasis in the absence of IRP1. Moreover, they support the notion that disruption of mitochondrial iron and redox balance may be a common underlying factor contributing to fasting hypoglycemia in young *Irp1^–/–^* mice and to the metabolic responses of adult *Irp1^–/–^* mice to both HFD and IDD.

### Metabolic reprogramming in Irp1^–/–^ mice.

**Figure 6:**
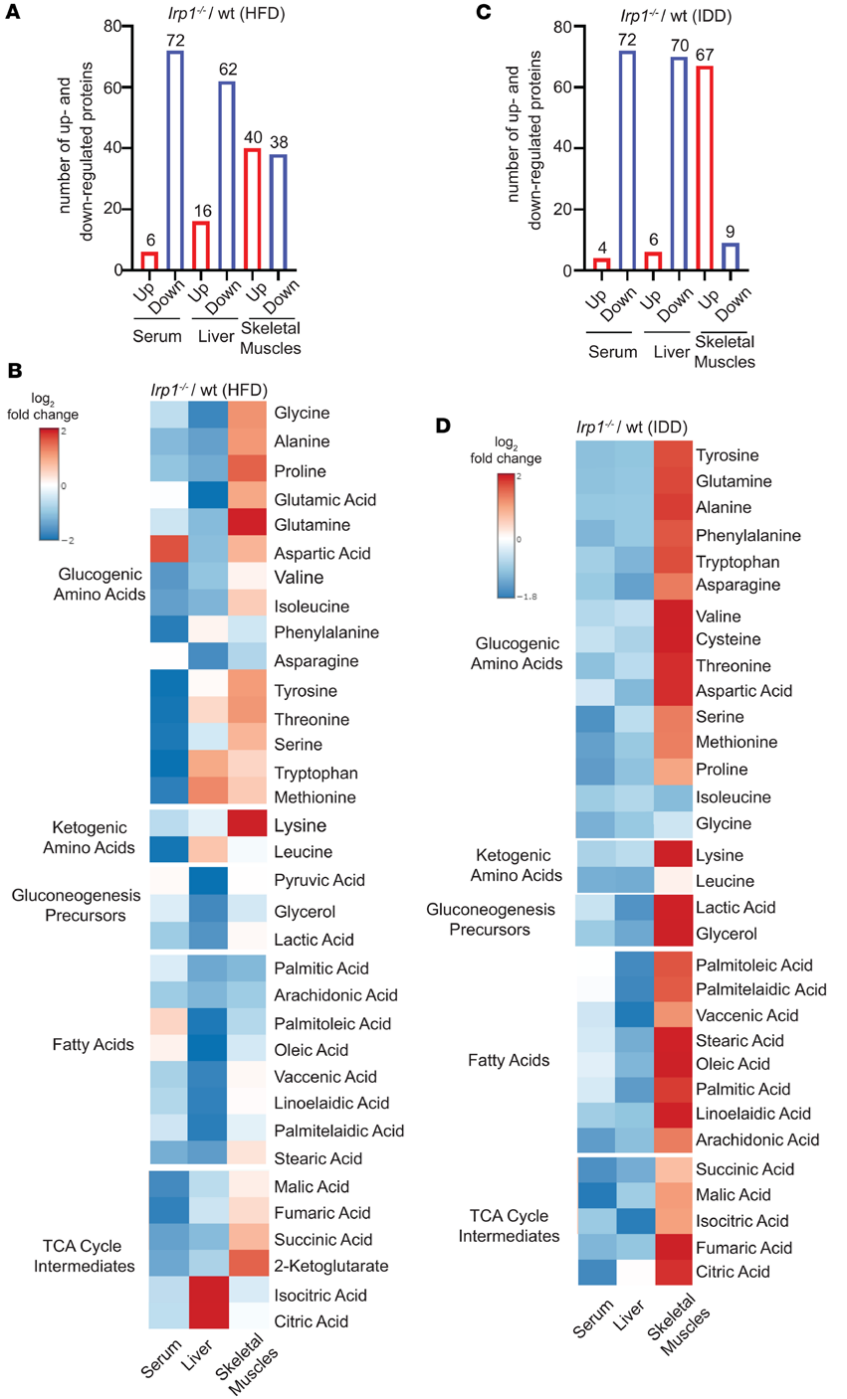
IRP1 deficiency triggers metabolic reprogramming in mice fed high-fat or iron-deficient diets. For 10 weeks, 5-week-old male *Irp1^–/–^* mice and WT littermates (*n* = 4–5 per experimental group) were provided a high-fat diet (HFD) or an iron-deficient diet (IDD). At the endpoint, the mice were euthanized, and serum, liver, and skeletal muscle samples were collected and processed for targeted metabolomics analysis. (**A** and **B**) Number of up- and down-regulated metabolites in serum, liver, and skeletal muscle of *Irp1^–/–^* versus WT on HFD (**A**); heatmap of detected glucogenic amino acids, ketogenic amino acids, gluconeogenesis precursors, fatty acids, and TCA cycle intermediates (**B**). (**C** and **D**) Number of up- and down-regulated metabolites in serum, liver, and skeletal muscle of *Irp1^–/–^* versus WT on IDD (**C**); heatmap of detected glucogenic amino acids, ketogenic amino acids, gluconeogenesis precursors, fatty acids, and TCA cycle intermediates (**D**). In **B** and **D**, log_2_ fold changes are indicated on the left.

Targeted metabolomics analysis in the serum, liver, and skeletal muscles was performed to uncover systemic metabolic alterations triggered by IRP1 deficiency after HFD or IDD intake. Normalized metabolomics data are provided in Supplemental Data Set 3. A total of 78 metabolites were detected in the serum, liver, and skeletal muscles of WT and *Irp1^–/–^* mice on an HFD; a complete heatmap is shown in Supplemental Figure 10A. Under these conditions, *Irp1^–/–^* mice exhibited lower levels of most serum and liver metabolites compared with WT controls (Figure 6A). We focused on changes in expression of specific metabolites such as glucogenic amino acids, ketogenic amino acids, gluconeogenesis precursors, fatty acids, and TCA cycle intermediates (Figure 6B). Among these, only aspartic acid was upregulated in serum, and citric and isocitric acid in the liver of *Irp1^–/–^* mice. By contrast, skeletal muscles from *Irp1^–/–^* mice on an HFD were enriched in glucogenic amino acids and TCA cycle intermediates, with the highest abundance of glutamine, proline, and 2-ketoglutarate; expression of ketogenic lysine was also high. Enrichment analysis identified the pathways most significantly affected by IRP1 deficiency and HFD: (a) tryptophan metabolism and starch and sucrose metabolism in serum (Supplemental Figure 10B); (b) glyoxylate and dicarboxylate metabolism, alanine aspartate and glutamate metabolism, TCA cycle, and unsaturated fatty acid biosynthesis in the liver (Supplemental Figure 10C); and (c) arginine and proline metabolism, pantothenate and CoA biosynthesis, and pyrimidine metabolism in skeletal muscles (Supplemental Figure 10D).

Under conditions of dietary iron restriction, the abundance of most analyzed metabolites was likewise lower in the serum and liver of *Irp1^–/–^* versus WT mice (Figure 6, C and D, and Supplemental Figure 11A). Notably, IDD intake resulted in a dramatic shift of metabolites to the skeletal muscles of these animals. Thus, 67 out of 76 detected metabolites were upregulated in *Irp1^–/–^* versus WT skeletal muscles, including glucogenic amino acids, gluconeogenesis precursors, fatty acids, and TCA cycle intermediates. Pathways most significantly affected by IRP1 and iron deficiency include the following: (a) lipoic acid metabolism; butanoate metabolism; and alanine, aspartate, and glutamate metabolism in serum (Supplemental Figure 11B); (b) starch and sucrose metabolism, as well as tryptophan metabolism in the liver (Supplemental Figure 11C); and (c) TCA cycle, glyoxylate and dicarboxylate metabolism, and biotin metabolism in skeletal muscles (Supplemental Figure 11D).

We also analyzed the impact of IRP1 deficiency under physiological conditions in mice fed the control diet. With few exceptions, metabolite levels were generally lower in *Irp1^–/–^* versus WT mice across the serum, liver, and skeletal muscles (Supplemental Figure 12, A and B). Enrichment analysis identified the following pathways as most affected: (a) glyoxylate and dicarboxylate metabolism; alanine, aspartate, and glutamate metabolism; and glycolysis/gluconeogenesis in serum (Supplemental Figure 12C); (b) histidine, metabolism, propanoate metabolism, pyruvate metabolism, TCA cycle, and glyoxylate and dicarboxylate metabolism in the liver (Supplemental Figure 12D); and (c) glycerophospholipid, fructose and mannose metabolism, inositol metabolism, glycerolipid metabolism, and glycolysis/gluconeogenesis in skeletal muscles (Supplemental Figure 12E). These data reveal extensive metabolic reprogramming in response to IRP1 deficiency, which is reflected in serum, liver, and skeletal muscle metabolites. HFD or IDD intake triggers a shift of metabolites from the liver and serum to skeletal muscles of *Irp1^–/–^* mice.

### IRP1 deficiency improves insulin sensitivity in hepatocytes and skeletal muscle cells.

**Figure 7:**
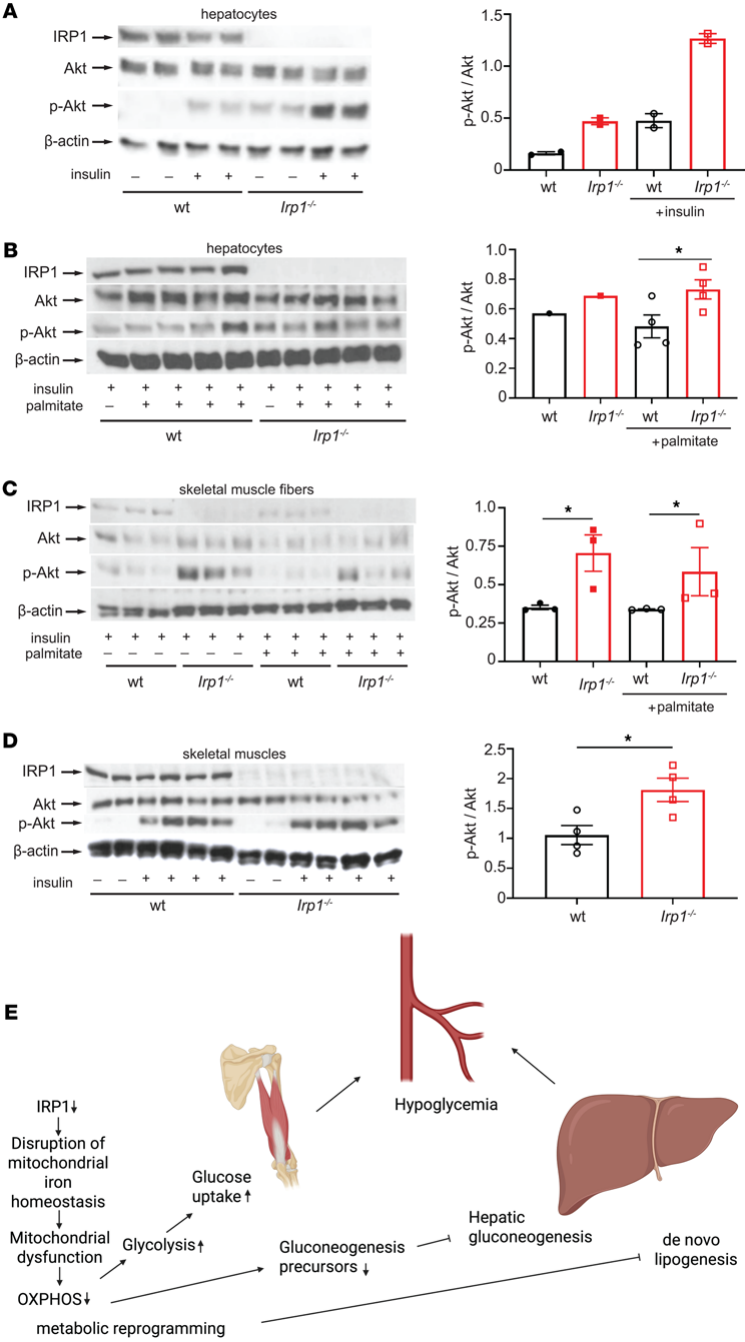
IRP1 deficiency stimulates insulin signaling. (**A**) Western blot analysis of phospho-Akt, Akt, IRP1, and β-actin in primary hepatocytes from *Irp1^–/–^* and WT mice either left untreated or previously treated with 10 nM insulin for 5 minutes. (**B**) Western blot analysis of phospho-Akt, Akt, IRP1, and β-actin in primary hepatocytes from *Irp1^–/–^* and WT mice pretreated for 24 hours with 0.4 mM palmitate (conjugated to BSA) or not, and then treated with 10 nM insulin for 5 minutes. (**C**) Western blot analysis of phospho-Akt, Akt, IRP1, and β-actin in skeletal muscle fibers from *Irp1^–/–^* and WT mice pretreated for 24 hours with 0.4 mM palmitate (conjugated to BSA) or not, and then treated with 50 nM insulin for 20 minutes. (**D**) Male *Irp1^–/–^* and WT mice on high-fat diet (HFD) for 10 weeks were injected with 4U/kg insulin or not. After 5 minutes, the mice were euthanized, and skeletal muscles were analyzed by Western blotting for expression of phospho-Akt, Akt, IRP1, and β-actin. (**E**) A model highlighting metabolic implications of IRP1 deficiency. In **A**–**D**, all Western blots show representative samples from each condition. Phospho-Akt to Akt ratios were quantified by densitometry and plotted on the right of each Western blot. Quantitative data are presented as mean ± SEM. Statistical analysis was performed with 2-way ANOVA with Tukey’s multiple-comparison test; comparisons between 2 groups with 2-tailed Student’s *t* test. *P < 0.05.

We explored whether the observed metabolic reprogramming in *Irp1^–/–^* mice is associated with altered insulin signaling. To this end, primary hepatocytes from WT and *Irp1^–/–^* mice remained untreated or treated with insulin, and downstream signaling was monitored by analyzing Akt phosphorylation as a surrogate marker (Figure 7A). We found p-Akt/Akt ratios were highly increased in insulin-treated *Irp1^–/–^* hepatocytes compared with insulin-treated WT hepatocytes (Figure 7A). Moreover, p-Akt/Akt ratios remained higher in *Irp1^–/–^* versus WT hepatocytes after treatment with palmitate, a fatty acid that causes insulin resistance (Figure 7B).

A similar experiment was performed with skeletal muscle fibers isolated from WT and *Irp1^–/–^* mice (Figure 7C). We found p-Akt/Akt ratios were increased in insulin-treated *Irp1^–/–^* versus WT muscle fibers and remained elevated after palmitate administration. To validate this finding in vivo, WT and *Irp1^–/–^* mice on an HFD were injected with insulin or not, and skeletal muscle tissue was analyzed by Western blotting. Insulin injection stimulated Akt phosphorylation in both genotypes (Figure 7D). However, p-Akt/Akt ratios were significantly higher in *Irp1^–/–^* versus WT skeletal muscles, again indicating increased insulin sensitivity. These data suggest that IRP1 deficiency increases insulin sensitivity and ameliorates fat-induced insulin resistance in the liver and skeletal muscles, in agreement with the metabolic phenotype of *Irp1^–/–^* mice.

## Discussion

*Irp1^–/–^* mice were initially considered to lack any discernible pathology (21). Nevertheless, further studies revealed that these animals develop polycythemia and pulmonary hypertension due to aberrant HIF2α-dependent transcriptional induction of erythropoietin in renal interstitial fibroblasts and hepatocytes, and of endothelin 1 in pulmonary endothelial cells, respectively (6–8). The mechanism involves translational de-repression of the IRE-containing HIF2α mRNA due to IRP1 deficiency. HIF2α and its homologue HIF1α are sensitive to oxygen- and iron-dependent posttranslational modification by prolyl-hydroxylases (PHD1, PHD2, or PHD3), which leads to their proteasomal degradation via the von Hippel-Lindau (VHL) E3 ubiquitin ligase complex (22). Modest HIF2α stabilization in the mouse liver (by acute deletion of *Phd3*) has been associated with improved insulin sensitivity and relative protection against type 2 diabetes following induction of *Irs2*, another HIF2α target gene (13, 14). Notably, in patients with Chuvash polycythemia, a condition characterized by VHL mutation that impairs HIF2α and HIF1α degradation, polycythemia correlates with low glycemia (23). Moreover, increased erythropoietic activity has been linked to low glycemia in the context of myeloproliferative neoplasms, hypoxia, or erythropoietin therapy (24, 25).

Consistent with these data, we observed that *Irp1^–/–^* mice exhibit more pronounced insulin responsiveness and glucose clearance compared with WT littermates, even after HFD feeding (Figure 1). These responses were likewise associated with a trend for *Irs2* induction in the liver, arguing for potential HIF2α involvement. This is further supported by the partial correction of rapid glucose disposal in *Irp1^–/–^* mice with hepatocyte-specific ablation of HIF2α versus *Irp1^–/–^* mice with intact HIF2α (Figure 1E). A modest HIF2α induction due to IRP1 deficiency could also contribute to suppressed *Srebp1*-induced lipogenesis and protection of *Irp1^–/–^* mice against liver steatosis (14) (Figure 2). Another argument supporting a role of HIF2α is provided by the correction of fasting hypoglycemia in older *Irp1^–/–^* mice, similar to polycythemia (6), and by the abrogation of this effect under HIF2α-stabilizing (26) dietary iron restriction (Figure 1D and Figure 5K). However, expression of gluconeogenic genes was not suppressed in *Irp1^–/–^* mice (Figure 1, K and L, and Supplemental Figure 1F), despite increased insulin sensitivity. This finding also contrasts with data in mouse models of modest or more severe hepatocyte-specific HIF2α stabilization due to acute ablation of *Phd3* (13, 14) or constitutive ablation of *Vhl* (27), respectively. Both approaches led to inhibition of gluconeogenic gene expression via either stimulation of insulin signaling (13, 14) or repression of glucagon signaling (27). Moreover, constitutive HIF2α upregulation in *Vhl*-deficient hepatocytes is known to cause severe steatosis in adult mice on a standard chow diet (28), while *Irp1^–/–^* mice were protected against liver steatosis even after HFD feeding (Figure 2).

Our initial data suggested a more complex phenotype for *Irp1^–/–^* mice that cannot be fully explained by molecular responses of modest HIF2α upregulation in hepatocytes, and may also be linked to energy deprivation and involve other tissues. In fact, hypoglycemia of *Irp1^–/–^* mice on a standard diet was severe and only comparable to models of hepatic HIF2α overexpression above a favorable metabolic threshold (14). We speculate that this accounts for the 18% lower survival rate that we noted for these animals at the age of 4 weeks. Embryonic lethality has been reported for mice with a βgeo gene trap construct inserted into the *Irp1* locus but not for *Irp1^–/–^* mice (29). The differences may be related to the genetic background of the animals, dietary factors, and possibly also the approximately 4.5 times lower number of mice genotyped in the previous study (29).

The liver proteomics analysis (Figure 3) provided further hints for altered energy metabolism in *Irp1^–/–^* mice. It uncovered potential mitochondrial dysfunction and changes in expression of proteins involved in oxidative phosphorylation and energy metabolism. We corroborated these data by Western blot analysis of respiratory chain proteins (Figure 3E) and by assessment of mitochondrial respiration in MEFs, primary hepatocytes, and differentiated myotubes from *Irp1^–/–^* mice (Figure 4 and Supplemental Figure 7). All these cell types manifested defective mitochondrial respiration and a switch to aerobic glycolysis for energy metabolism, also known as the Warburg effect in cancer cells (30), which was accompanied by increased proliferation rates of MEFs and myoblasts (Supplemental Figure 8, B and C). The phenotype was more pronounced in MEFs and differentiated myotubes. Skeletal muscle fibers from *Irp1^–/–^* mice exhibited increased glucose uptake, consistent with the high need for nutrients for energy production via less efficient aerobic glycolysis (30). Importantly, differentiated myotubes displayed aberrant oxygen consumption with palmitate as substrate, indicating defective β-oxidation (Figure 4F), which correlated with relative protection against insulin resistance (Figure 7C). These data support the conclusion from an earlier study that “dietary fat is less damaging to skeletal muscle metabolic function under conditions of constrained β-oxidation” (31).

We provide evidence that mitochondrial dysfunction is caused by disruption of iron redox balance in this organelle due to lack of IRP1 (Figure 5). Our findings offer an additional link between iron and intermediary metabolism (32). HFD feeding has been associated with responses to iron starvation in the liver, such as upregulation of TfR1 expression (19), possibly via IRP1 (20). These data are largely validated in Figure 5A. Even though we did not directly compare changes in mitochondrial iron content in response to an HFD, we speculate that increased supply of metabolically active iron to mitochondria is needed to cope with the metabolic stress imposed by long-term exposure to an HFD. Another limitation of our study is that we did not validate the mitochondrial dysfunction data from primary cell cultures using isolated tissue mitochondria. Conversely, we did not validate the iron speciation data from tissues using primary cell cultures.

The critical role of iron is also underlined by the observations that dietary iron restriction maintained hypoglycemia (Figure 1D), efficient glucose clearance (Figure 5K), and poor gluconeogenesis (Figure 5L) in *Irp1^–/–^* mice, and the glucose phenotype was partially reversed by iron dextran injection (Figure 5M). These findings are in line with previous studies showing that iron deficiency causes mitochondrial dysfunction and uncoupling of oxidative phosphorylation in the liver (33), impairs the gluconeogenic capacity of isolated hepatocytes (34), enhances glucose catabolism in skeletal muscles (35), and increases peripheral insulin responsiveness (36, 37). Moreover, they are consistent with the protective effects of iron deficiency and mitochondrial dysfunction against insulin resistance caused by an HFD in rats (38).

Our data suggest that not only iron content but also iron redox balance is critical for mitochondrial function. Notably, the loss of IRP1, a cytosolic protein, disrupted iron redox balance in mitochondria but not in the cytosol (Figure 5). In liver mitochondria, *Irp1^–/–^* mice exhibited an increase in the Fe^2+^/Fe^3+^ ratio, primarily driven by elevated Fe^2+^ concentrations (Figure 5F and Supplemental Figure 9, C and D). Excess Fe^2+^ may represent a metabolically inert iron pool that fails to enter bioenergetic pathways, potentially increasing redox vulnerability. In contrast, in skeletal muscle mitochondria from *Irp1^–/–^* mice, the Fe^2+^/Fe^3+^ ratio was lower due to decreased Fe^2+^ and elevated Fe^3+^ levels (Figure 5J and Supplemental Figure 9, E and F), pointing to oxidative sequestration and reduced availability of metabolically active Fe^2+^. In both cases, redox imbalance of iron causes mitochondrial dysfunction.

Our data highlight the important role of IRP1 in mitochondrial iron metabolism, especially under stress, and corroborate previous findings in diverse settings. For instance, *Fxn^Alb-Cre^* mice with hepatocyte-specific frataxin disruption exhibited mitochondrial dysfunction that was exacerbated upon crossing with *Irp1^–/–^* mice due to defective mitochondrial iron import (39). In another example, targeted hepatocyte-specific ablation of both IRP1 and IRP2 caused liver failure and death in mice due to mitochondrial iron deficiency and dysfunction (5); these data also imply a contribution of IRP2. Indeed, MEFs from either *Irp1^–/–^* or *Irp2^–/–^* mice had compromised mitochondrial Fe-S cluster biogenesis due to repressed frataxin and IscU expression (18), while IRP2 deficiency triggered a switch from oxidative phosphorylation to aerobic glycolysis in MEFs (40). Along these lines, proteomics analysis revealed reduced expression of several mitochondrial electron transport chain proteins in bone marrow–derived macrophages lacking both IRP1 and IRP2 (41). However, contrary to the metabolic phenotype of *Irp1^–/–^* mice reported herein, *Irp2^–/–^* animals exhibit glucose intolerance and develop diabetes (11). Thus, the systemic metabolic functions of IRP1 and IRP2 are distinct. Dissecting the mechanism by which IRP1 modulates mitochondrial iron metabolism awaits further investigation. We speculate that this may involve posttranscriptional regulation of known and/or yet unidentified downstream mRNA targets. Alternatively, IRP1 may control mitochondrial iron traffic via protein-protein interactions; a recent study identified IRP1 as an interacting partner of MEMO1, a protein linked to mitochondrial iron supply (42).

Our metabolomics analysis uncovered extensive metabolic reprogramming in the liver and skeletal muscles of *Irp1^–/–^* mice, presumably due to mitochondrial dysfunction and inefficient energy production. These responses were exacerbated under conditions of high energetic needs following metabolic stress (HFD) or dietary iron restriction (Figure 6 and Supplemental Figures 10 and 11), and are reminiscent of metabolic changes reported in *Tfr1^mu/mu^* mice bearing skeletal muscle–specific disruption of TfR1 (43). In this animal model, TfR1 deficiency in skeletal muscles caused severe iron deficiency, incapacitating mitochondrial energy production that led to growth arrest and a muted attempt to switch to fatty acid β-oxidation, consuming fat stores. Even though hepatic gluconeogenesis was stimulated, amino acid substrates became limiting, and lethal hypoglycemia developed shortly after birth.

In *Irp1^–/–^* mice on an IDD or HFD, amino acid substrates likewise became limiting for gluconeogenesis, which might also have been impaired by energy deprivation. Paradoxically, insulin signaling was stimulated in these animals rather than suppressed (Figure 7). This very likely contributed to IDD-induced hypoglycemia or protection against HFD-induced hyperglycemia, and increased glucose disposal by skeletal muscles. The protection against HFD-induced hyperglycemia was more prominent in male mice (Figure 1F and Supplemental Figure 1E). The molecular basis for the apparent sexual dimorphism is not clear and will be addressed in follow-up studies.

On a final note, while *Irp1^–/–^* mice were initially considered to lack any apparent phenotype (21), we and others demonstrated that these animals develop polycythemia due to translational de-repression of renal HIF2α mRNA and subsequent transcriptional induction of its downstream target erythropoietin (6–8). The physiological relevance of our findings was subsequently validated by a meta-analysis of genome-wide association studies involving 684,122 individuals from Iceland and the United Kingdom, which identified the IRP1-encoding *ACO1* gene as a major homeostatic regulator of hemoglobin concentration (44). We expect that the metabolic phenotype of *Irp1^–/–^* mice reported herein will spark analogous validation studies.

In conclusion, our data uncover a potentially novel function of IRP1 as a metabolic switch (Figure 7E) and provide evidence that this involves its iron regulatory activity. Moreover, our data suggest that targeting IRP1 may offer therapeutic benefits against hyperglycemia, insulin resistance, and metabolic dysfunction–associated steatotic liver disease, the hepatic manifestation of metabolic syndrome (45).

## Methods

### Sex as a biological variable.

Early experiments were performed using both male and female mice. Because the metabolic phenotypes of IRP1 deficiency were stronger in males, only male animals were used for subsequent mechanistic studies.

### Animals.

*Irp1^–/–^* mice on C57BL/6 background (29) were provided by M.W. Hentze (European Molecular Biology Laboratory). *Irp1^+/–^* mice were generated by breeding *Irp1^–/–^* with congenic *Irp1^+/+^* (WT) mice. Breeding pairs from these animals were used to generate *Irp1^+/+^* (WT) and *Irp1^–/–^* littermates that were used throughout this study. *Irp1^–/–^Hif2α^Alb-Cre^* and *Hif2α^Alb-Cre^* mice were as previously described (6). All animals were housed in Makrolon cages (up to 5 mice per cage, 12-hour light/12-hour dark cycle: 7:00 am to 7:00 pm; 22°C ± 1°C, 60% ± 5% humidity) according to institutional guidelines. Where indicated, the mice were fed an HFD (43% fat; ssniff Spezialdiäten GmbH, S9552-E034) or its equivalent control diet (12% fat; ssniff Spezialdiäten GmbH, S9552-E033). In other experiments, the mice were fed an IDD (TD.80396 [Envigo/Inotiv] containing 2–6 ppm iron) or its equivalent control diet (TD.89173, [Envigo/Inotiv] 18% protein, 2918 containing 200 ppm iron).

### Cell culture.

MEFs were obtained from WT and *Irp1^–/–^* mouse embryos according to standard procedures. Briefly, embryos were excised from the uterus at 10–12 days of gestation. The head, appendages, and organs were removed from the embryo. Embryos were minced in PBS and added to a 50 mL centrifuge tube with 0.1% collagenase (MilliporeSigma) solution in DMEM (Invitrogen). The cells were filtered through a silk filter, spun at 300*g* for 10 minutes, resuspended in culture media and plated on a 10 cm dish in a CO_2_ incubator at 37°C. The cells were cultured in DMEM supplemented with 10% heat-inactivated FBS (Wisent), nonessential amino acids, 100 U/mL penicillin, and 100 μg/mL streptomycin, and immortalized using a pBABE-neo large T antigen cDNA (Addgene plasmid 1780). Primary hepatocytes were prepared from adult mice using a 2-step collagenase perfusion technique and cultured as described (46). Primary myoblasts were prepared from 4- to 6-week-old mice as described (47). Differentiation to myotubes was in DMEM supplemented with 5% horse serum for 5 days. Muscle fibers were isolated from soleus muscle (48) and cultured in DMEM supplemented with 10% FBS and antibiotics.

### Glucose, pyruvate, and insulin tolerance tests.

*Irp1^–/–^* mice and WT littermates were fasted for 4–16 hours before the experiments. The animals were intraperitoneally injected with 1 g/kg glucose for the GTT, 2 g/kg sodium pyruvate for the pyruvate tolerance test, or 0.5 U/kg insulin (Humulin, DIN 00586714) for the insulin tolerance test. For oral GTT, the mice were given 2 g/kg glucose by oral gavage. Where indicated, mice were intraperitoneally injected with iron dextran (Sigma-Aldrich) at 15 mg/kg, 3 times with 2-hour intervals; the last injection was performed 24 hours before the GTT. Blood glucose levels were measured at 0, 15, 30, 60, and 120 minutes using the OneTouch Verio Flex blood glucose meter.

### Glucose uptake assay.

Isolated muscle fibers were starved in glucose-deficient DMEM for 45 minutes before the assay. Subsequently, the media were replaced with DMEM containing 4.5 g/L glucose and supplemented with 1% FBS and antibiotics, and the muscle fibers were incubated for 6 hours. Culture supernatants (100 μL) were collected at 0, 3, and 6 hours, and glucose was measured by using glucose oxidase liquid reagents (Pointe Scientific, G7521120). Glucose uptake was calculated as the difference of glucose amount collected at the zero time point and the specific time points. Data were normalized to total protein.

### Seahorse experiments.

Mitochondrial respiration and glycolysis were analyzed in MEFs, primary hepatocytes, or differentiated myotubes by the Seahorse assay (49). Details are provided in the Supplemental Methods.

### Lactate assay.

Cells were cultured in DMEM supplemented with 1% fetal FBS and antibiotics (100 U/mL penicillin, 100 μg/mL streptomycin). Old media were replaced with fresh, and aliquots (100 μL) were collected at 3, 6, and 24 hours. Lactate was measured using a lactate oxidase reagent kit (Pointe Scientific, L7596-50). Data were normalized to total protein.

### Isolation of cytosolic and mitochondrial fractions.

Liver and skeletal muscles from *Irp1^–/–^* mice and WT littermates were processed to separate mitochondrial and cytosolic fractions by using a mitochondria isolation kit (Thermo Fisher Scientific, 89801).

### Iron redox speciation analysis.

For redox speciation analysis of Fe^2+^ and Fe^3+^, the method outlined previously (50) was significantly modified to reduce the overall analysis time (including analysis and column cleaning) and to optimize detection for inductively coupled plasma kinetic energy discrimination mass spectrometry. Details are provided in the Supplemental Methods.

### Quantitative real-time reverse-transcription PCR.

Total RNA was extracted from tissues by using the RNeasy kit (QIAGEN). Purity was assessed by 260/280 nm absorbance ratios, and quality was monitored by agarose gel electrophoresis. cDNA was synthesized from 1 μg RNA by using the OneScript Plus cDNA Synthesis Kit (Applied Biological Materials). Quantitative real-time reverse-transcription PCR was performed in a 7500 Fast Real Time PCR System (Applied Biosystems) with gene-specific primers provided in the Reagents and Resources section of the supplemental materials. Primer pairs were validated by dissociation curve analysis and demonstrated amplification efficiency between 90% and 110%. SYBR Green (Bioline) and primers were used to amplify products under the following cycling conditions: initial denaturation 95°C for 10 minutes, 40 cycles of 95°C for 5 seconds, 58°C for 30 seconds, 72°C for 10 seconds, and the final cycle melt analysis between 58°C and 95°C. Relative mRNA expression was calculated by the 2^–*ΔΔ*Ct^ method (51). Data were normalized to murine ribosomal protein L19 (*Rpl19*).

### Western blotting.

Western blot analysis was performed as described earlier (52). Antibodies and dilutions are provided in the Reagents and Resources section of the supplemental materials. Immunoreactive bands were quantified by densitometry with ImageJ (NIH) software.

### Histology.

Liver specimens from 3 mice per experimental group were fixed in 10% buffered formalin and embedded in paraffin. Samples were cut at 4 μm, placed on SuperFrost/Plus slides (Thermo Fisher Scientific), and dried overnight at 37°C. De-paraffinized slides were used for H&E or PAS staining.

### Lipidomics, proteomics, metabolomics studies, and other assays.

The experimental outline and details are provided in the Supplemental Methods.

### Statistics.

Quantitative data are expressed as mean ± SEM. Statistical analysis was performed using GraphPad Prism (v10.6.1). Multiple groups were analyzed by 2-way ANOVA except for 1E, which was 1-way, with Tukey’s post hoc comparisons test. Comparisons between 2 independent groups were done with an unpaired 2-tailed Student’s *t* test. A *P* value of less than 0.05 was considered statistically significant.

### Study approval.

All experimental procedures were approved by the IACUC of McGill University (protocol 4966).

### Data availability.

The mass spectrometry proteomics data have been deposited to the ProteomeXchange Consortium via the PRIDE partner repository (dataset identifier PXD051897). The lipidomics raw data have been deposited to the MassIVE repository (project accession MassIVE MSV000100449). The metabolomics raw data have been deposited to Mendeley Data (https://data.mendeley.com/datasets/tjwvzfk4wg/2). Quantified liver proteomics and lipidomics data and normalized metabolomics data are provided in supplemental spreadsheets. The Supporting Data Values file is also provided.

## Author contributions

WG contributed to the investigation, formal analysis, methodology, and writing of the original draft. NW and TZ contributed to the investigation. CF was responsible for formal analysis, methodology, and project administration. AK, BJ, BM, CHB, DMB, HZ, JS, KS, VDS, and VV were responsible for methodology. EB and VR contributed to methodology and formal analysis. ZL contributed to the formal analysis. OI was responsible for methodology and conceptualization. GS contributed to the methodology, resources, and conceptualization. KP was responsible for conceptualization, supervision, funding acquisition, and review and editing of the manuscript.

## Funding support

- Canadian Institutes of Health Research (PJT-186193).
- Deutsche Forschungsgemeinschaft (VE 1249/1-1) to BM and VV.

## Supplementary Material

Supplemental data

Supplemental data set 1

Supplemental data set 2

Supplemental data set 3

Unedited blot and gel images

Supporting data values

## Version 1 01/06/2026

In-Press Preview

## Version 2 02/23/2026

Electronic publication

## References

[1] Galy B (2024). Mechanisms controlling cellular and systemic iron homeostasis. Nat Rev Mol Cell Biol.

[2] Wilkinson N, Pantopoulos K (2014). The IRP/IRE system in vivo: insights from mouse models. Front Pharmacol.

[3] Smith SR (2006). Complete loss of iron regulatory proteins 1 and 2 prevents viability of murine zygotes beyond the blastocyst stage of embryonic development. Blood Cells Mol Dis.

[4] Galy B (2008). Iron regulatory proteins are essential for intestinal function and control key iron absorption molecules in the duodenum. Cell Metab.

[5] Galy B (2010). Iron regulatory proteins secure mitochondrial iron sufficiency and function. Cell Metab.

[6] Wilkinson N, Pantopoulos K (2013). IRP1 regulates erythropoiesis and systemic iron homeostasis by controlling HIF2α mRNA translation. Blood.

[7] Anderson SA (2013). The IRP1-HIF-2α axis coordinates iron and oxygen sensing with erythropoiesis and iron absorption. Cell Metab.

[8] Ghosh MC (2013). Deletion of iron regulatory protein 1 causes polycythemia and pulmonary hypertension in mice through translational derepression of HIF2α. Cell Metab.

[9] Cooperman SS (2005). Microcytic anemia, erythropoietic protoporphyria, and neurodegeneration in mice with targeted deletion of iron-regulatory protein 2. Blood.

[10] Galy B (2005). Altered body iron distribution and microcytosis in mice deficient in iron regulatory protein 2 (IRP2). Blood.

[11] Santos M (2020). Irp2 regulates insulin production through iron-mediated Cdkal1-catalyzed tRNA modification. Nat Commun.

[12] Beltran-Sanchez H (2013). Prevalence and trends of metabolic syndrome in the adult U.S. population, 1999-2010. J Am Coll Cardiol.

[13] Wei K (2013). A liver Hif-2α-Irs2 pathway sensitizes hepatic insulin signaling and is modulated by Vegf inhibition. Nat Med.

[14] Taniguchi CM (2013). Cross-talk between hypoxia and insulin signaling through Phd3 regulates hepatic glucose and lipid metabolism and ameliorates diabetes. Nat Med.

[15] Geisler CE (2016). Hepatic adaptations to maintain metabolic homeostasis in response to fasting and refeeding in mice. Nutr Metab (Lond).

[16] Wakil SJ, Abu-Elheiga LA (2009). Fatty acid metabolism: target for metabolic syndrome. J Lipid Res.

[17] Zhao T (2024). MnO_2_ nanoparticles trigger hepatic lipotoxicity and mitophagy via mtROS-dependent Hsf1^Ser326^ phosphorylation. Free Radic Biol Med.

[18] Li H (2018). Iron regulatory protein deficiency compromises mitochondrial function in murine embryonic fibroblasts. Sci Rep.

[19] Padda RS (2015). A high-fat diet modulates iron metabolism but does not promote liver fibrosis in hemochromatotic Hjv–/– mice. Am J Physiol Gastrointest Liver Physiol.

[20] Dongiovanni P (2015). High fat diet subverts hepatocellular iron uptake determining dysmetabolic iron overload. PLoS One.

[21] Meyron-Holtz EG (2004). Genetic ablations of iron regulatory proteins 1 and 2 reveal why iron regulatory protein 2 dominates iron homeostasis. EMBO J.

[22] Lee P (2020). Cellular adaptation to hypoxia through hypoxia inducible factors and beyond. Nat Rev Mol Cell Biol.

[23] McClain DA (2013). Decreased serum glucose and glycosylated hemoglobin levels in patients with Chuvash polycythemia: a role for HIF in glucose metabolism. J Mol Med (Berl).

[24] Rao TN (2019). JAK2-mutant hematopoietic cells display metabolic alterations that can be targeted to treat myeloproliferative neoplasms. Blood.

[25] Scherer T (2025). A direct effect of the hematocrit on blood glucose: evidence from hypoxia- and erythropoietin-treated mice. Sci Adv.

[26] Schwartz AJ (2019). Hepatic hepcidin/intestinal HIF-2α axis maintains iron absorption during iron deficiency and overload. J Clin Invest.

[27] Ramakrishnan SK (2016). HIF2α Is an essential molecular brake for postprandial hepatic glucagon response independent of insulin signaling. Cell Metab.

[28] Rankin EB (2009). Hypoxia-inducible factor 2 regulates hepatic lipid metabolism. Mol Cell Biol.

[29] Galy B (2005). Generation of conditional alleles of the murine Iron Regulatory Protein (IRP)-1 and -2 genes. Genesis.

[30] Vander Heiden MG (2009). Understanding the Warburg effect: the metabolic requirements of cell proliferation. Science.

[31] Koves TR (2008). Mitochondrial overload and incomplete fatty acid oxidation contribute to skeletal muscle insulin resistance. Cell Metab.

[32] Fillebeen C (2020). Regulatory Connections between Iron and Glucose Metabolism. Int J Mol Sci.

[33] Masini A (1994). Dietary iron deficiency in the rat. I. Abnormalities in energy metabolism of the hepatic tissue. Biochim Biophys Acta.

[34] Klempa KL (1989). Iron deficiency decreases gluconeogenesis in isolated rat hepatocytes. J Appl Physiol (1985).

[35] Henderson SA (1986). Glucose turnover and oxidation are increased in the iron-deficient anemic rat. Am J Physiol.

[36] Farrell PA (1988). Increased insulin sensitivity in iron-deficient rats. J Nutr.

[37] Borel MJ (1993). Hepatic glucose production and insulin sensitivity and responsiveness in iron-deficient anemic rats. Am J Physiol.

[38] Han DH (2011). Deficiency of the mitochondrial electron transport chain in muscle does not cause insulin resistance. PLoS One.

[39] Martelli A (2015). Iron regulatory protein 1 sustains mitochondrial iron loading and function in frataxin deficiency. Cell Metab.

[40] Li H (2019). Iron regulatory protein 2 modulates the switch from aerobic glycolysis to oxidative phosphorylation in mouse embryonic fibroblasts. Proc Natl Acad Sci U S A.

[41] Sanchez M (2011). Iron regulatory protein-1 and -2: transcriptome-wide definition of binding mRNAs and shaping of the cellular proteome by iron regulatory proteins. Blood.

[42] Dolgova N (2024). MEMO1 binds iron and modulates iron homeostasis in cancer cells. Elife.

[43] Barrientos T (2015). Metabolic catastrophe in mice lacking transferrin receptor in muscle. EBioMedicine.

[44] Oskarsson GR (2020). Predicted loss and gain of function mutations in ACO1 are associated with erythropoiesis. Commun Biol.

[45] Targher G (2024). MASLD: a systemic metabolic disorder with cardiovascular and malignant complications. Gut.

[46] Fillebeen C (2018). Hepcidin-mediated hypoferremic response to acute inflammation requires a threshold of Bmp6/Hjv/Smad signaling. Blood.

[47] Lazure F, Farouni R, Sahinyan K, Blackburn DM, Hernandez-Corchado A, Perron G (2023). Transcriptional reprogramming of skeletal muscle stem cells by the niche environment. Nat Commun.

[48] Sahinyan K (2022). Application of ATAC-Seq for genome-wide analysis of the chromatin state at single myofiber resolution. Elife.

[49] Divakaruni AS, Jastroch M (2022). A practical guide for the analysis, standardization and interpretation of oxygen consumption measurements. Nat Metab.

[50] Solovyev N (2017). Redox speciation of iron, manganese, and copper in cerebrospinal fluid by strong cation exchange chromatography - sector field inductively coupled plasma mass spectrometry. Anal Chim Acta.

[51] Livak KJ, Schmittgen TD (2001). Analysis of relative gene expression data using real-time quantitative PCR and the 2(-Delta Delta C(T)) Method. Methods.

[52] Katsarou A (2021). Tissue-specific regulation of ferroportin in wild-type and hjv-/- mice following dietary iron manipulations. Hepatol Commun.

