# Supplementary material for: IRP1 deficiency alters mitochondrial metabolism and protects against metabolic syndrome pathologies
Source: JCI Insight. 2026 Jan 6;11(4):e183247. doi: 10.1172/jci.insight.183247 (PMC12956002; doi:10.1172/jci.insight.183247)
Supplement: Supplemental data [file jciinsight-11-183247-s111.pdf]

Supplemental Methods

Seahorse experiments

MEFs, primary hepatocytes, or differentiated myotubes from *Irp1*$^{-/-}$ mice and wild type littermates were seeded onto a Seahorse XF96 Cell Culture Microplate at a density of $2x10^4$/well or $1x10^4$/well and stored overnight at 37°C in a humidified CO_2 incubator. XFe96 sensor cartridge probes were immersed in the calibrant and stored overnight at 37°C in a non-CO_2 incubator. For cell media preparation, 8 mM glucose, 3 mM glutamine, and 1 mM pyruvate were added to Agilent XF base medium, and the pH was adjusted to 7.4. Each well was washed twice with supplemented base media, and the plate was incubated for 1 hour in a 37°C non-CO_2 incubator. For mitochondria stress assays, 1 µM oligomycin, 1.5 µM carbonyl cyanide-4-(trifluoromethoxy)phenylhydrazone (FCCP), and 1 µM rotenone/antimycin were loaded into designated drug loading ports. For the fatty acid oxidation assay, cells were incubated in DMEM supplemented with 0.5 mM glucose, 1 mM GlutaMAX, 0.5 mM carnitine, and 1% fetal bovine serum 24 hours before the assay. The assay media contained 111 mM NaCl, 4.7 mM KCl, 1.25 mM $CaCl_2$, 2 mM $MgSO_4$, 1.2 mM NaH_2PO_4, 2.5 mM glucose, 0.5 mM carnitine, and 5 mM HEPES. 2.5 µM oligomycin, 1.6 µM FCCP, and 2 µM rotenone/antimycin were loaded into each well. Prior to assay start, 30 µl of 1 mM palmitate-BSA (Cayman Chemical 29558) was added to each well. For the glycolytic rate assay, 0.5 µM rotenone/antimycin and 50 mM 2-deoxy-D-glucose (2-DG) were loaded into the drug loading ports. The cartridge and cell plate were loaded into a Seahorse XFe96 Analyzer for analysis. Oxygen consumption data were analyzed as described (1).

Iron speciation analysis

The separation of the redox species Fe^{2+} and Fe^{3+} was conducted using a NexSAR® PEEK HPLC gradient system, which featured an autosampler programmed for a 50 µl sample volume, a column oven maintained at 30 °C, and a Dionex IonPac™ CS5A RFIC analytical cationic column (4 x 250 mm). For the detection of iron in the chromatograms, the column was directly connected to the NexIon® ICP mass spectrometer (PerkinElmer, Rodgau-Jügesheim, Germany), which operated in KED mode and was equipped with platinum cones, a glass concentric nebulizer, and a cyclone spray chamber (PerkinElmer, Shelton, CT, USA).

The HPLC conditions were as follows: Eluent A consisted of 50 mM ammonium citrate and 7.0 mM PDCA at pH 4.2, while Eluent B contained 30 mM EDTA and 250 mM NH_4Ac at pH 6. Unlike the method in (2), a gradient elution was implemented to expedite column cleaning after each run and to ensure reproducibility. Gradient elution was programmed as follows:
0-2 min: 0% B, flow rate: 0.5 ml/min, 2-4 min: : 0% B, flow rate: 0.5 → 0.8 ml/min, 4-6 min: 0 → 50% B, flow rate: 0.8 → 0.85 ml/min, 6-7.5 min: 50 → 100% B, flow rate: 0.85 ml/min, 7.5-9.5 min: 100% B, flow rate: 0.85 ml/min, 9.5-12.5 min: 0% B, flow rate: 0.85 ml/min, 12.5-13 min: 0% B, flow rate: 0.85 → 0.5 ml/min.

The operating conditions for the NexIon® ICP mass spectrometer were: RF power was set to 1,200 W, with a plasma gas flow of 16 l/min, an auxiliary gas flow of 1.05 l/min, and a nebulizer gas flow of 0.98 l/min (optimized daily). Helium was utilized for KED mode at a flow rate of 1 ml/min. The isotopes ^{56}Fe and ^{57}Fe were monitored during the analysis. Clarity software was used for comprehensive instrument control of both the NexSAR® PEEK HPLC gradient system and the NexIon® ICP mass spectrometer, as well as for the evaluation of iron chromatograms and peak area calculations. The Fe^{2+}/Fe^{3+} ratios were determined based on the respective peak areas following normalization to protein content of the subcellular extract.

Lipidomics

The protein-precipitation liquid extraction protocol described previously (3). Briefly, to each sample (~25 mg of liver tissue) we added 400 µl of chloroform, along with a single 5 mm stainless steel ball bearing. The samples were then homogenised using a Bioprep 24-1004 homogenizer (Allsheng, Hangzhou, China) run at speed; 4.5 m/s, time; 30 s for 2 cycles. Following this, 250 µl of chloroform, 250 µl of methanol and 100 µl of the LIPID+CARNITINE internal standard (1-10 µM in methanol), and 400 µl of acetone was added to each sample. The samples were vortexed thoroughly. The samples were then centrifuged for 10 min at ~20,000 g to pellet any insoluble material. The supernatant was pipetted into separate 2 ml screw cap amber-glass auto-sampler vials (Agilent Technologies, Cheadle, United Kingdom). The extracts were dried down to dryness using a Concentrator Plus system (Eppendorf, Stevenage, United Kingdom) run for 60 min at 60°C.

Lipidomics LC-MS acquisition and data analysis

For lipidomics LC-MS analysis, the samples were reconstituted in 100 µl of 2:1:1 (propan-2-ol, acetonitrile and water, respectively) then thoroughly vortexed. The reconstituted sample was transferred into a 250 µl low-volume vial insert inside a 2 ml amber glass auto-sample vial ready for liquid chromatography with mass spectrometry detection (LC-MS) lipidomics analysis. Full chromatographic separation of intact lipids was achieved using Waters Acquity H-Class HPLC System (Waters, Hertfordshire, United Kingdom) with the injection of 10 µl onto a Waters Acquity Premier UPLC® CSH C18 column; 1.7 µm, I.D. 2.1 mm X 50 mm, maintained at 55ºC. Mobile phase A was 6:4, acetonitrile and water with 10 mM ammonium formate. Mobile phase B was 9:1, propan-2-ol and acetonitrile with 10 mM ammonium formate. The flow was maintained at 500 µl/min through the following gradient: 0.00 minutes_40% mobile phase B; 1.5 minutes_40% mobile phase B; 8.00 minutes_99% mobile phase B; 10.00 minutes_99% mobile phase B; 10.10 minutes_40% mobile phase B; 12.00 minutes_40% mobile phase B. The sample injection needle was washed using 9:1, propan-2-ol and acetonitrile [strong wash] and 2:1:1 (propan-2-ol, acetonitrile and water) [weak wash]. The mass spectrometer used was the Thermo Scientific Q-Exactive Orbitrap with a heated electrospray ionisation source (Thermo Fisher Scientific, Hemel Hempstead, United Kingdom). The mass spectrometer was calibrated immediately before sample analysis using positive and negative ionisation calibration solution (recommended by Thermo Scientific). Additionally, the mass spectrometer scan rate was set at 4 *Hz*, giving a resolution of 35,000 (at 200 *m/z*) with a full-scan range of *m/z* 120 to 1,800 with continuous switching between positive and negative mode. The data processing involved the integration of the extracted ion chromatogram peaks for each target lipid species (circa 1,600 species) and the stable isotope labelled internal standards at their expected retention time. The area ratio response of the target lipid to the corresponding internal standard were converted into µM for the fluid samples or nmole results normalised to the amount of tissue extracted. All results were subjected to blank correction and comprehensive quality checking before further statistical analysis. Data analysis was performed by using the MetaboAnalyst 6.0 software. Data were rescaled to set the reference values of lipids obtained from wild type mouse samples and analyzed by MetaboAnalyst 6.0 to plot the heatmap.

Proteomics

Proteomics analysis was carried out at the Proteomics Center of the Lady Davis Institute. Liver tissue samples were lysed in a buffer containing 5% sodium dodecyl sulfate (SDS), 100 mM TRIS pH 7.8. Samples were subsequently heated to 99°C for 10 minutes and subjected to probe-based sonication using a Thermo Sonic Dismembrator at 25 % amplitude for 3 cycles x 5 seconds. The remaining debris was pelleted by centrifugation at 20,000 x g for 5 minutes. An aliquot of the supernatant was diluted to <1% SDS and used for estimation of protein concentration by bicinchoninic acid assay (BCA). Lysates were clarified by centrifugation at 14,000 x g for 5 minutes and transferred into a new reaction tube. Disulfide bonds were reduced by the addition of tris(2-carboxyethyl)phosphine (TCEP) to a final concentration of 20 mM and incubated at 60°C for 30 minutes. Free cysteines were alkylated using iodoacetamide at a final concentration of 30 mM and subsequent incubation at 37°C for 30 minutes in the dark. An equivalent of 200 µg of total protein was used for proteolytic digestion using suspension trapping (S-TRAP). Proteins were acidified by adding phosphoric acid to a final concentration of 1.3% v/v. Samples were then diluted 6-fold in STRAP loading buffer (9:1 methanol:water in 100 mM TRIS, pH 7.8) and loaded onto an S-TRAP Mini cartridge (Protifi LLC, Huntington NY) prior to centrifugation at 2000 x g for 2 minutes. Samples were washed three times with 350 µL of STRAP loading buffer, and proteolytically digested using trypsin (Sigma) at a 1:10 enzyme to substrate ratio for 16 hours at 37°C. Peptides were sequentially eluted in 50 mM ammonium bicarbonate, 0.1% formic acid in water, and 50% acetonitrile. Peptide containing samples underwent solid phase extraction using Oasis HLB, 30 mg, 1CC cartridges (Waters). Peptide samples were dried and reconstituted in 0.1% trifluoro acetic acid (TFA) prior to analysis by mass spectrometry.

Proteomics LC-MS/MS acquisition

Samples were analyzed by data dependent acquisition (DDA) using an Easy-nLC 1200 online coupled to a Q Exactive Plus (both Thermo Fisher Scientific). Samples were first loaded onto a pre-column (Acclaim PepMap 100 C18, 3 µm particle size, 75 µm inner diameter x 2 cm length) in 0.1% formic acid (buffer A). Peptides were then separated using a 100-min binary gradient ranging from 3-40% B (84% acetonitrile, 0.1% formic acid) on the analytical column (Acclaim PepMap 100 C18, 2 µm particle size, 75 µm inner diameter x 25 cm length) at 300 nL/min. MS spectra were acquired from m/z 350-1,500 at a

resolution of 70,000, with an automatic gain control (AGC) target of 1 x 106 ions and a maximum injection time of 50 ms. The 15 most intense ions (charge states +2 to +4) were isolated with a window of m/z 1.2, an AGC target of 2 x 104 and a maximum injection time of 64 ms and fragmented using a normalized higher-energy collisional dissociation (HCD) energy of 28. MS/MS spectra were acquired at a resolution of 17,500 and the dynamic exclusion was set to 40 s. DDA MS raw data was processed with Proteome Discoverer 2.5 (Thermo Scientific) and searched using Sequest HT (4) against the canonical mouse SwissProt FASTA database downloaded from Uniprot. The enzyme specificity was set to trypsin with a maximum of 2 missed cleavages. Carbamidomethylation of cysteine was set as static modification and methionine oxidation as variable modification. The precursor ion mass tolerance was set to 10 ppm, and the product ion mass tolerance was set to 0.02 Da. The percolator node was used and the data were filtered using a false discovery rate (FDR) cut-off of 1% at both the peptide and protein level. The Minora feature detector node of Proteome Discoverer was used for precursor-based label free quantitation.

Analysis of proteomics data

Proteins quantified by at least 1 protein unique peptide were further filtered based on a minimum of >50% valid values in at least one of the two sample groups. Remaining missing values were imputed by low abundance sampling within Proteome Discoverer 2.5. LFQ abundances were scaled (normalized) based on the total amount of quantified peptides, and abundance ratios were calculated as the ratio of grouped protein abundances. Statistical significance was determined by background-adjusted t-tests and adjusted for false discovery rate (FDR) using the Benjamini-Hochberg method within Proteome Discoverer 2.5. Regulation was defined by having an adjusted p value of less than 0.05 and an expression ratio cut-off of 2-fold. Dimensional reduction by principal component analysis and hierarchical clustering were conducted using the normalized protein and phosphopeptide LFQ abundances in Instant Clue (http://www.instantclue.uni-koeln.de/). Functional enrichment analysis of protein-protein interaction networks was performed using STRINGDB analysis with the StringApp for visualization within Cytoscape (v. 3.9.1). Functional annotation was performed in the gProfiler tool (5).

Targeted metabolomics

Metabolomics profiling of serum, liver and quadriceps skeletal muscle samples was carried out at the Metabolomics Innovation Resource (MIR) of the Goodman Cancer Institute (GCI) at McGill University. The analysis was targeted to citric acid cycle intermediates, glycolytic intermediates, free or total fatty acids, and amino acids and derivatives. For quantification of circulating metabolites, 25 µl serum was mixed with 500 µl 80% methanol and centrifuged; the supernatant was collected and prepared for GC-MS analysis. For quantification of tissue metabolites, 10 mg liver or skeletal muscle tissue was pulverized and mixed with 1 ml 80% methanol. The mixture was processed using a bead beater for 2 min at 30Hz for two rounds at 4°C. After centrifugation, the supernatant was collected and prepared for GC-MS. 1 µl 750 ng/µl of myristic acid-d27 standard (dissolved in pyridine) was added to each sample. The samples were dried overnight in a refrigerated speedvac. Dried pellets were dissolved in 30 ml of pyridine containing 10 mg/ml methoxyamine-HCl using a sonicator and vortex. Samples were incubated for 30 min at 70°C before transferring to GC-MS injection vials containing 70 µl MTBSTFA. The vials were centrifuge at 10,000g for 10 min. Samples were incubated at 70°C for 1 h and then analyzed by GC-MS.

Metabolomics GC-MS acquisition and data analysis

Samples were randomized and analyzed by an Agilent 5975C Series GC/MSD with the Triple-Axis Detector GC-MS systems using both selective ion monitoring (SIM) and full scan mode. Metabolite data were collected by the Chemstation software and normalized to total protein abundance. Data were analyzed by MetaboAnalyst 6.0 to generate KEGG-based pathway enrichment analysis. Data were rescaled to set the reference values of metabolites obtained from wild type mouse samples and analyzed by MetaboAnalyst.ca to plot the heatmap.

Metabolic cage experiment

To continuously and simultaneously monitor food and water intake, a group of male *Irp1*$^{+/-}$ mice and wild type littermates (n=4-7) previously maintained on high fat diet (HFD) for 10 weeks was transferred to metabolic cages (TSE Phenomaster, TSE systems, Chesterfield, MO) in the facilities of the McGill University Health Center (MUHC). The animals continued feeding the HFD. Each cage was equipped with Aspen chip bedding, a food hopper, and a water bottle integrated into a monitoring system embedded in

the cage lid. Physical activity was recorded in real time using the BXYZ beam break activity monitoring system. Mice were allowed to acclimate to the metabolic cages for 7 days under standard housing conditions. Metabolic measurements were performed during a period of 4 days using TSE Phenomaster (TSE systems, Chesterfield, MO). Environmental conditions were tightly controlled, with ambient temperature maintained at 22 °C and a 12-hour light–dark cycle (lights on at 7:00 a.m. and off at7:00 p.m.) to synchronize circadian activity patterns.

Complete blood count measurements and serum biochemistry

Blood from mice was collected with cardiac puncture. Complete blood count (CBC) values were acquired by using 12 µl of whole blood per sample and the Scil Vet-ABC hematology analyzer. Serum was separated by centrifugation and used to determine iron concentration and Tf saturation by a Roche Hitachi 917 Chemistry Analyzer.

Measurement of splenic iron

Splenic iron content was quantified by using a colorimetric ferrozine-based assay (6).

Measurement of blood pressure

Systolic and diastolic blood pressure were measured using the MC4000 Multi-Channel Blood Pressure Analysis System (Hatteras Instruments). The measurements were repeated for five consecutive days, and the values from the last two days were used for analysis.

Aconitase assay

Heart tissues from *Irp1*$^{-/-}$ mice and wild type littermates were processed to isolate mitochondrial fractions by using a mitochondria isolation kit (Thermo Fisher). Mitochondrial aconitase activity was measured by a colorimetric assay using an aconitase assay kit (Sigma). Data were normalized to total mitochondrial protein levels.

Reagents and Resources

REAGENT or RESOURCE	SOURCE	IDENTIFIER
Antibodies		
Rabbit anti-IRP1 (2000X)	Home Made (7)	NA
Mouse anti-TfR1 (1000X)	Invitrogen	Cat# 13-6800, RRID: AB_2533029
Rabbit anti-FTH1 (1000X)	Cell Signaling	Cat# 4393, RRID: AB_11217441
Rabbit anti-Akt (1000X)	Cell Signaling	Cat# 9272, RRID: AB_329827
Rabbit anti-phospho-Akt (Ser473) (1000X)	Cell Signaling	Cat# 4060, RRID: AB_2315049
Mouse anti-OXPHOS (2000X)	Abcam	Cat# 110413, RRID: AB_2629281
Mouse anti-FXN (1000X)	Thermo Fisher	Cat# PA5-95829, RRID: AB_2807631
Mouse anti-ISCU (1000X)	Thermo Fisher	Cat# 14812-1-AP, RRID: AB_2280362
Rabbit anti-Mitoferrin1 (1000X)	Thermo Fisher	Cat# 26469-1-AP, RRID: AB_2880527
Rabbit anti-Tom20 (1000X)	Cell Signaling	Cat# 42406, RRID: AB_2687663
Rabbit anti-actin (1000X)	Sigma-Aldrich	Cat# A2066, RRID: AB_476693
Chemicals, Peptides, and Recombinant Proteins		
Collagenase D	Roche	Cat# 11088858001
Bovine Serum Albumin	BioShop	Cat# 9048-46-8
Trypsin	Wisent	Cat# 325-043-EL
Fetal Bovine Serum	Wisent	Cat# 080150
Penicillin-Streptomycin	Wisent	Cat# 450-201-EL

Dulbecco's Modified Eagle's Medium	Wisent	Cat# 319-005-CL
Dulbecco's Modified Eagle's Medium, no glucose	Wisent	Cat# 319-061-CL
Dulbecco's Modified Eagle's Medium, no glucose, no phenol red	Gibco	Cat# A14430-01
XF Base Medium Minimal DMEM	Agilent	Cat# 103334-100
Geneticin Selective Antibiotic (G418 Sulfate)	Wisent	Cat# 451-130-QL
Leibovitz's L-15 Medium	Gibco	Cat# 11415064
William's E Medium	Wisent	Cat# 301-018-CL
Horse Serum	Wisent	Cat# 065-150
Ham's F-10 Nutrient Mix	Gibco	Cat# 11550043
Human FGF-Basic Recombinant Protein	Gibco	Cat# PHG0261
HBSS	Wisent	Cat# 311-506-CL
Percoll	Cytiva	Cat# 17089101
Glutamine	Wisent	Cat# 609-065-EL
Phosphate-Buffered Saline	Wisent	Cat# 311-010-CL
2-Mercaptoethanol	Sigma	Cat# M3148
TEMED	BioShop	Cat# 110-18-9
4-(2-hydroxyethyl)-1-piperazineethanesulfonic acid)	BioShop	Cat# 7365-45-9
Ethylenediaminetetraacetic Acid	BioShop	Cat# 6381-92-6
Methoxyamine hydrochloride	Sigma	Cat# 593-56-6
N-tert-Butyldimethylsilyl-N-methyltrifluoroacetamide (MTBSTFA)	Sigma	Cat# 77626
$CaCl_2$	BioShop	Cat# 10035-04-8
NaOH	BioShop	Cat# 1310-73-2
Glucose	BioShop	Cat# 50-99-7
Pyruvate	BioShop	Cat# 113-24-6
Humulin (insulin)	Lily	Cat# 00586714
Iron Dextran	Sigma	Cat# D8517
GlutaMAX	Gibco	Cat# 35050061
Carnitine	Sigma	Cat# C0158
cOmplete Mini EDTA-free Protease Inhibitor Cocktail	Roche	Cat# 11836170001
Halt Phosphatase Inhibitor Cocktail	Thermo Fisher	Cat# 78420
KCl	Sigma	Cat# P9541
NaCl	BioShop	Cat# 7647-14-5
$CaCl_2$	BDH	Cat# B10070
$MgSO_4$	Sigma	Cat# M2773
NaH_2PO_4	BioShop	Cat# 10049-21-5
2-DG	Sigma	Cat# 154-17-6
Rotenone	Sigma	Cat# R8875
Antimycin	Sigma	Cat# A8674
Palmitate-BSA	Cayman Chemical	Cat# 29558
Tween-20	BioShop	Cat# 9005-64-5
Recombinant DNA		
pBABE-neo largeTcDNA	Addgene	Cat# 1780

Critical Commercial Assays		
DC Protein Assay	Bio-Rad	Cat# 5000111
BCA Protein Assay	Pierce	Cat# 23225
Glucose (Oxidase) Liquid Reagents	Pointe Scientific	Cat# G7521120
Lactate (Liquid) Reagent Set	Pointe Scientific	Cat# L7596-50
Mito Stress Kit	Agilent	Cat# 103015-100
Mitochondria Isolation Kit for Tissue	Thermo Fisher	Cat# 89801
Mouse Insulin ELISA Kit (Colorimetric)	Novus	Cat# NBP2-62853
Mouse Erythropoietin/EPO ELISA Kit - Quantikine	R&D Systems	Cat# MEP00B
Aconitase Assay Kit	Sigma	Cat# MAK337
RNeasy Kit	Qiagen	Cat# 74106
OneScript Plus cDNA Synthesis Kit	Applied Biological Materials	Cat# G236
Experimental Models: Organisms/Strains		
Mouse *Irp1-/-*	Maintained in this lab (originally (8))	NA
Oligonucleotides		
Fis1<br>Forward: 5'-AGCTGGAACGCCTGATTGAT-3'<br>Reverse: 5'-TGGAGACAGCCAGTCCAATG-3'		NA
Drp1<br>Forward: 5'-ATGCCTGTGGGCTAATGAAC-3'<br>Reverse: 5'-CCTGCAGATGGGACATTTTT-3'		NA
Mfn1<br>Forward: 5'-CCAAGCCCAACATCTTCATTGAT-3'<br>Reverse: 5'-AGCTTCCGACGGACTTACAA-3'		NA
Mfn2<br>Forward: 5'-GTCCTGGACGTCAAAGGGTA-3'<br>Reverse: 5'-GCAGAACTTTGTCCCAGAGC-3'		NA
G6pc		NA

Forward: 5’-TCTGTCCCGGATCTACCTTG-3’<br>Reverse: 5’-GTAGAATCCAAGCGCGAAAC-3’		
Irs1<br>Forward: 5’-CCAGCCTGGCTATTTAGCTG-3’<br>Reverse: 5’-CCCAACTCAACTCCACCACT		NA
Irs2<br>Forward: 5’-GCCTGGGGATAATGGTGACTA-3’<br>Reverse: 5’-TCCATGAGACTTAGCCGCTTC-3’		NA
Irp1<br>delR: 5’-TATACGAAGTTATACGTACCTGCAG-3’<br>WTS: 5’-ATGCAGTTACGTAGCCTCTAGGAG-3’<br>WTR: 5’-TAAGTCATCACAGCTCATGATCAGTC-3’		NA
Pck1<br>Forward: 5’-AGCCTTTGGTCAACAACTGG-3’<br>Reverse: 5’-TGCCTTCGGGGTTAGTTATC-3’		NA
Fbp1<br>Forward: 5’-TGCTGAAGTCGTCCTACGCTAC-3’<br>Reverse: 5’-TTCCGATGGACACAAGGCAGTC-3’		NA
Pgc1α<br>Forward: 5’-GTCAACAGCAAAAGCCACAA-3’<br>Reverse: 5’-TCTGGGGTCAGAGGAAGAGA-3’		NA
Rpl19<br>Forward: 5’-AGGCATATGGGCATAGGGAAGAG-3’		NA

Reverse: 5'-TTGACCTTCAGGTACAGGCTGTG-3'		
Srebp1<br>Forward: 5'-CCAGAGGGTGAGCCTGACAA-3'<br>Reverse: 5'-AGCCTCTGCAATTTCCAGATCT-3'		NA
Hamp1<br>Forward: 5'-AAGCAGGGCAGACATTGCGAT-3'<br>Reverse: 5'-CAGGATGTGGCTCTAGGCTATGT-3'		NA
Epo<br>Forward: 5'-AATGGAGGTGGAAGAACAGGCCAT-3'<br>Reverse: 5'-CGAAGCAGTGAAGTGAGGCTACGTA-3'		NA
Software and Algorithms		
GraphPad Prism 10.2.2	GraphPad Software	https://www.graphpad.com/
ImageJ 1.53k	National Institutes of Health	https://imagej.net/ij/download.html
Proteome Discoverer 2.5	Thermo Fisher	https://www.thermofisher.com/ca/en/home/industrial/mass-spectrometry/liquid-chromatography-mass-spectrometry-lc-ms/lc-ms-software/multi-omics-data-analysis/proteome-discoverer-software.html
Sequest HT	Eng, J. K., Ashley McCormack, A. L. and Yates, J. R. (4)	https://proteomicsresource.washington.edu/protocols06/sequest.php
gProfiler tool	Reimand, J., Arak, T., Adler, P., Kolberg, L. et al (5)	https://biit.cs.ut.ee/gprofiler/gost
MetaboAnalyst 6.0	Wishart Research Group	https://www.metaboanalyst.ca/MetaboAnalyst/
Chemstation LTS 01.11	Agilent	https://www.agilent.com/en/product/software-informatics/analytical-software-suite/chromatography-data-systems/openlab-chemstation

Composition of the high fat diet from sniff

Ingredient<br>Product No.	U<br>%	HF 43 kcal%<br>mixed Protein<br>S9552-E034
Casein	%	3.000
Poultry protein	%	8.500
Soy protein isolate	%	4.900
Wheat gluten, Vital	%	2.300
Egg yolk	%	1.600
Egg white	%	1.600
Whey powder, 71 % Lactose	%	2.900
L-Cystine	%	0.300
Corn starch	%	18.130
Maltodextrin	%	8.000
Sucrose	%	14.200
Fructose	%	3.800
Cellulose powder	%	5.000
Vitamin premixture	%	1.000
Mineral premixture + additions	%	4.300
Choline Cl (50 %)	%	0.290
L-Carnitine	%	0.050
Cholesterol	%	0.120
Butylated hydroxytoluene	%	0.010
Beef tallow	%	7.300
Pork lard	%	9.120
Butter fat, dehydrated	%	2.740
Corn oil	%	0.600
Canola oil	%	0.240

Supplemental figures

A

littermates at 4 weeks

IRP1 genotype	+/+	+/–	–/–
females	115	232	102
ratio	1	2.01	0.89
males	132	257	100
ratio	1	1.95	0.76
expected ratio	1	2	1

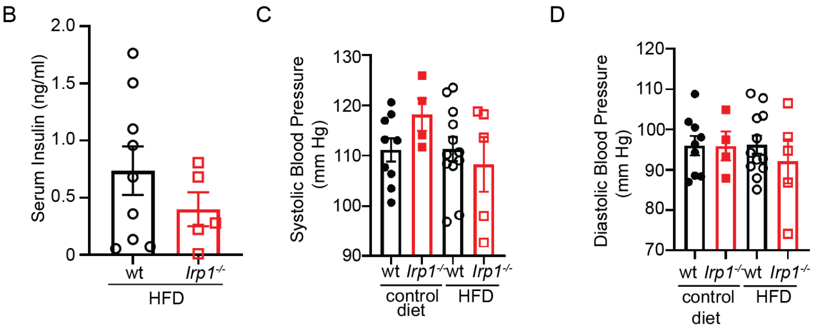

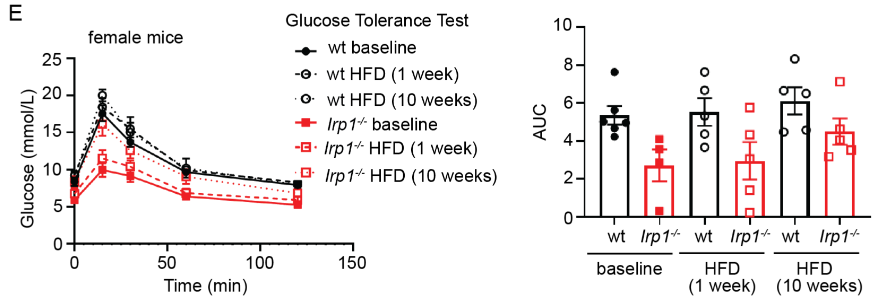

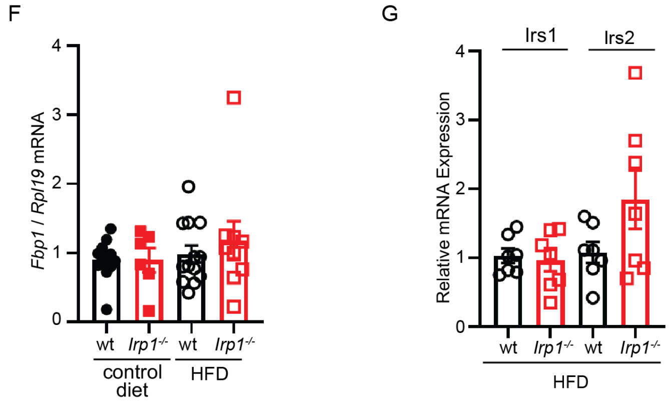

Fig. S1. (A) Sex-specific genotyping of all male and female *Irp1*$^{+/+}$, *Irp1*$^{+/-}$ and *Irp1*$^{-/-}$ littermates generated throughout this study. (B) Serum insulin levels of mice described in Fig. 1F (at t=0). (C) Systolic and (D) diastolic pressure of the mice described in Fig. 2 before sacrifice. (E) Glucose tolerance test (GTT) after 5 h fasting in female *Irp1*$^{-/-}$ and wild type littermate mice fed a control or a high-fat diet (HFD) for 10 weeks immediately after weaning. (F-G) qPCR analysis of liver *Fbp1* (G), *Irs1* (H) and *Irs2* (H) mRNA expression, as in mice described in Fig.1I-J. Quantitative data are presented as the mean±SEM. Statistical analysis was performed by ANOVA with Tukey's multiple comparisons test or with the Student's t test; no significant differences were found.

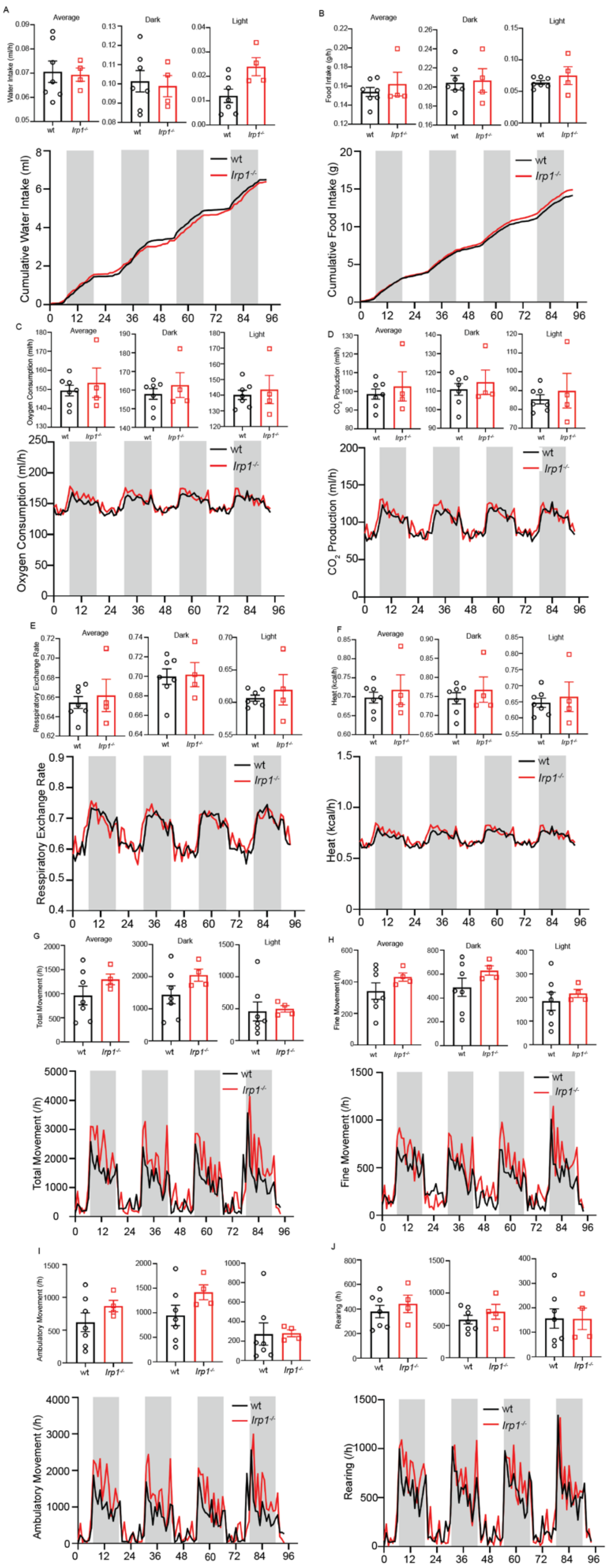

Fig. S2. Metabolic phenotyping of *Irp1*-/- and wild type mice. Male *Irp1*-/- mice and wild type littermates (n=4-7 per genotype) were fed immediately after weaning a high fat diet (HFD) for 10 weeks and transferred to metabolic cages, continuing the HFD. Following a 7-day acclimatization period, metabolic measurements were performed for 4 days. (A) Water intake. (B) Food intake. (C) Oxygen consumption. (D) CO_2 production. (E) Respiratory exchange rate. (F) Heat. (G) Total movement. (H) Fine movement. (I) Ambulatory movement. (J) Rearing. Cumulative data are shown in graphs. Quantitative average data, as well as average data during the dark and light periods, are presented as the mean±SEM.

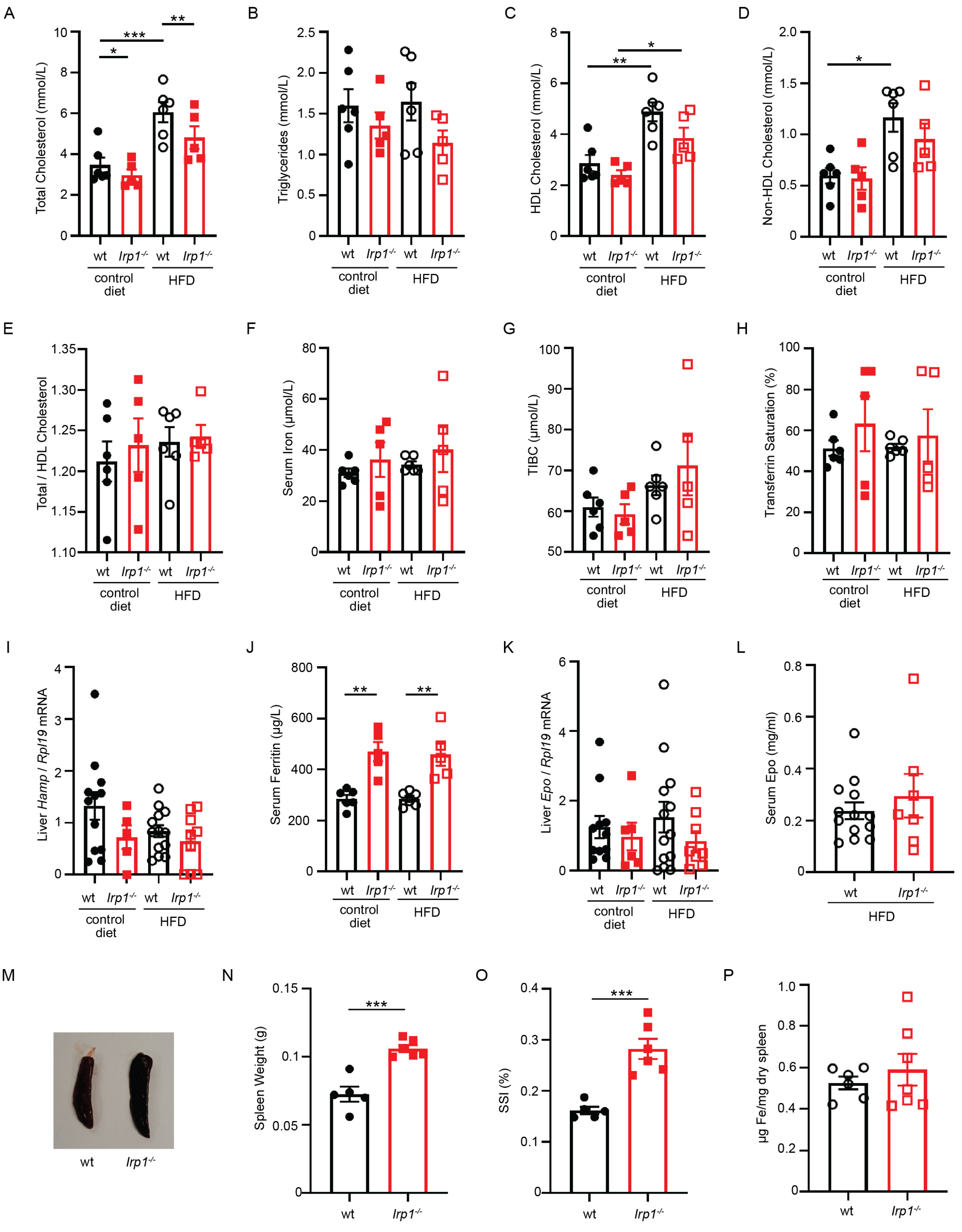

Fig. S3. Serum biochemistry, liver gene expression and splenosomatic index in *Irp1*$^{-/-}$ and wild type mice in response to dietary manipulations. Experimental outline is described in Fig. 2. (A) Total cholesterol. (B) Triglycerides. (C) HDL cholesterol. (D) Non-HDL cholesterol. (E) Total/HDL cholesterol ratio. (F) Serum

iron. (G) Total iron-binding capacity (TIBC). (H) Transferrin saturation. (I) qPCR analysis of liver *Hamp* mRNA. (J) Serum ferritin; (K) qPCR analysis of liver *Epo* mRNA. (L) Serum Epo. (M) Spleen from representative *Irp1*$^{-/-}$ and wild type. (N) Spleen weights. (O) Splenosomatic index (SSI). (P) Splenic iron content. Quantitative data are presented as the mean±SEM. Statistical analysis was performed by ANOVA with Tukey's multiple comparisons test; comparisons between two groups were done with the Student's t test. Significant differences are indicated by * ($p<0.05$), ** ($p<0.01$), or *** ($p<0.001$), respectively.

A

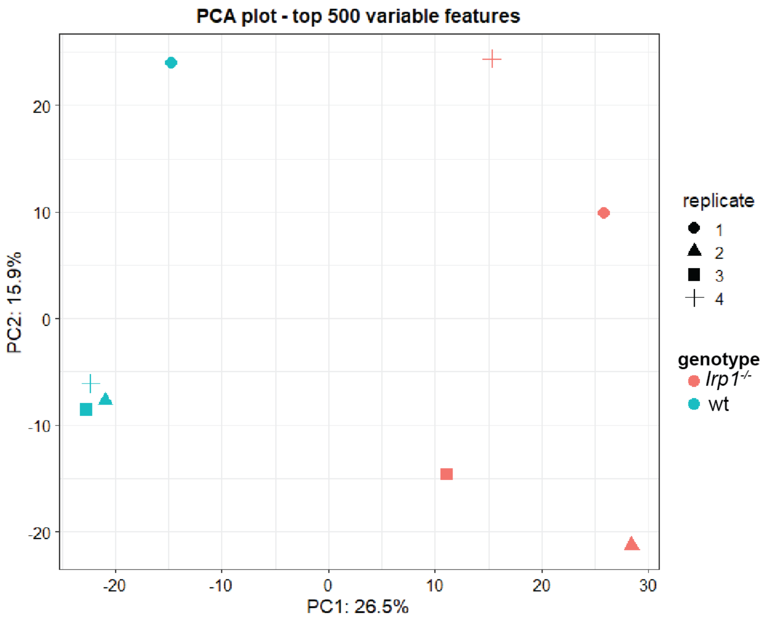

B

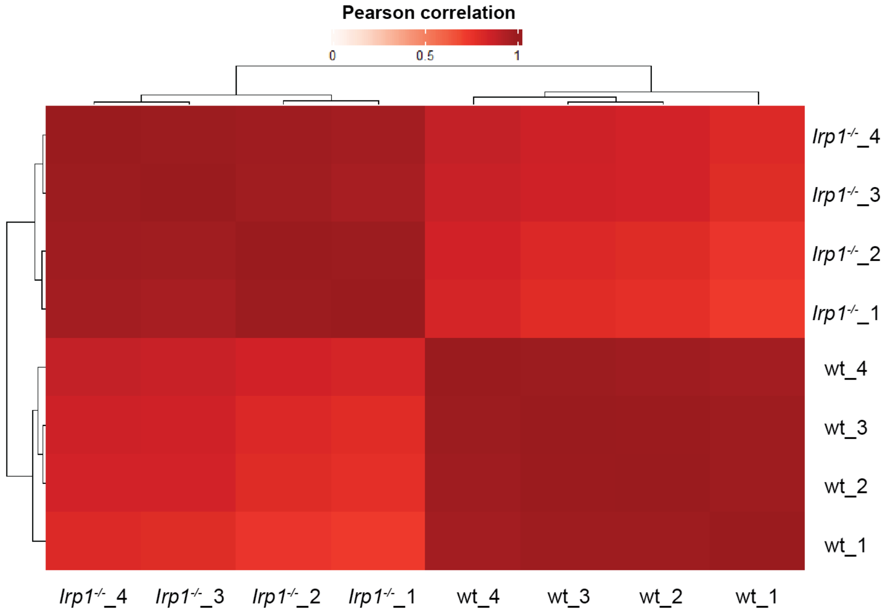

Fig. S4. Quality control of liver proteomics data from *Irp1*$^{-/-}$ and wild type mice described in Fig. 3. (A) Principal component analysis (PCA) and (B) Pearson correlation analysis.

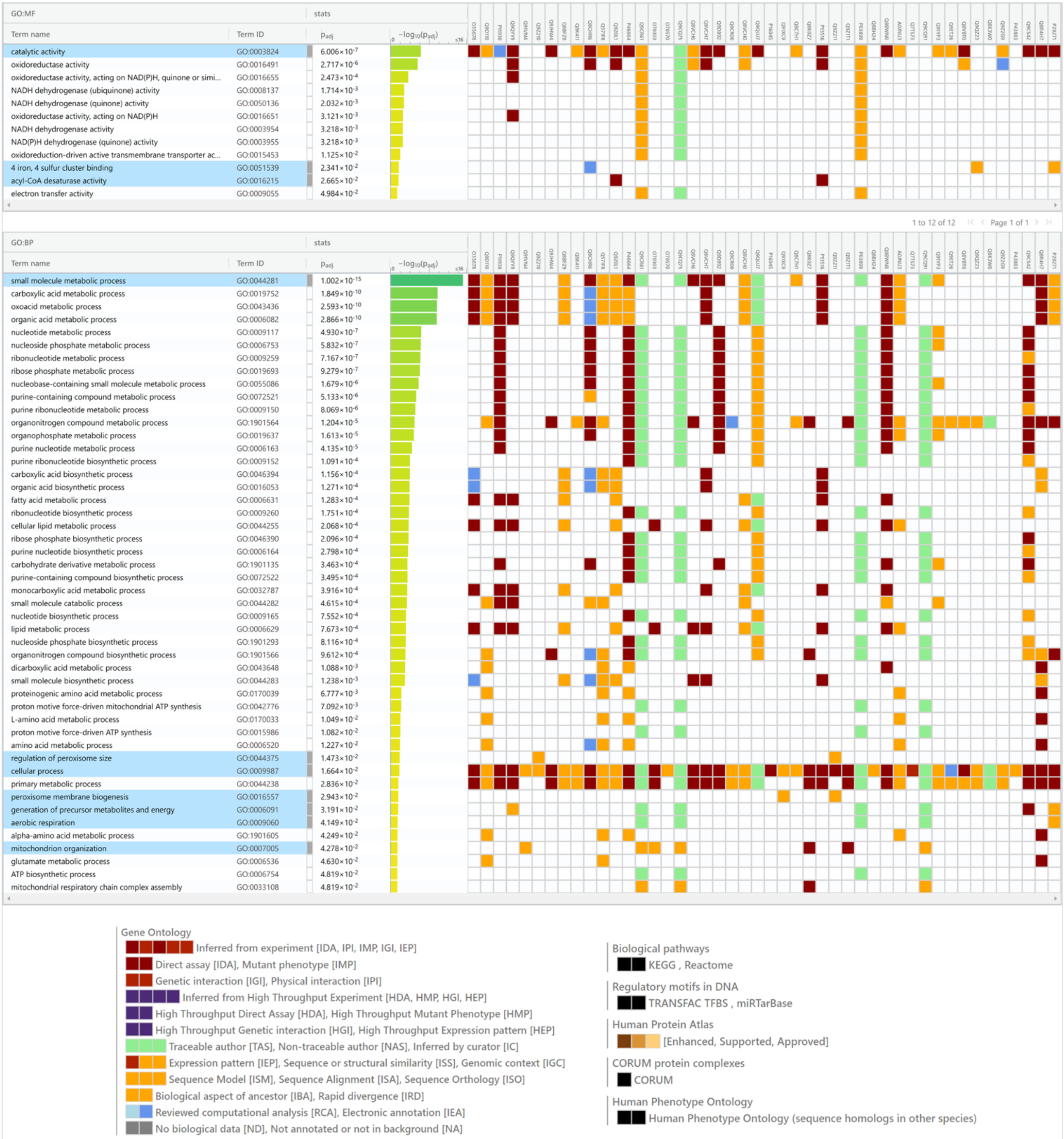

Fig. S5. Enrichment analysis of liver proteomics data from *Irp1*$^{-/-}$ and wild type mice described in Fig. 3. (A) Gene Ontology Molecular Function (GO:MF) and Gene Ontology Biological Process (GO:BP).

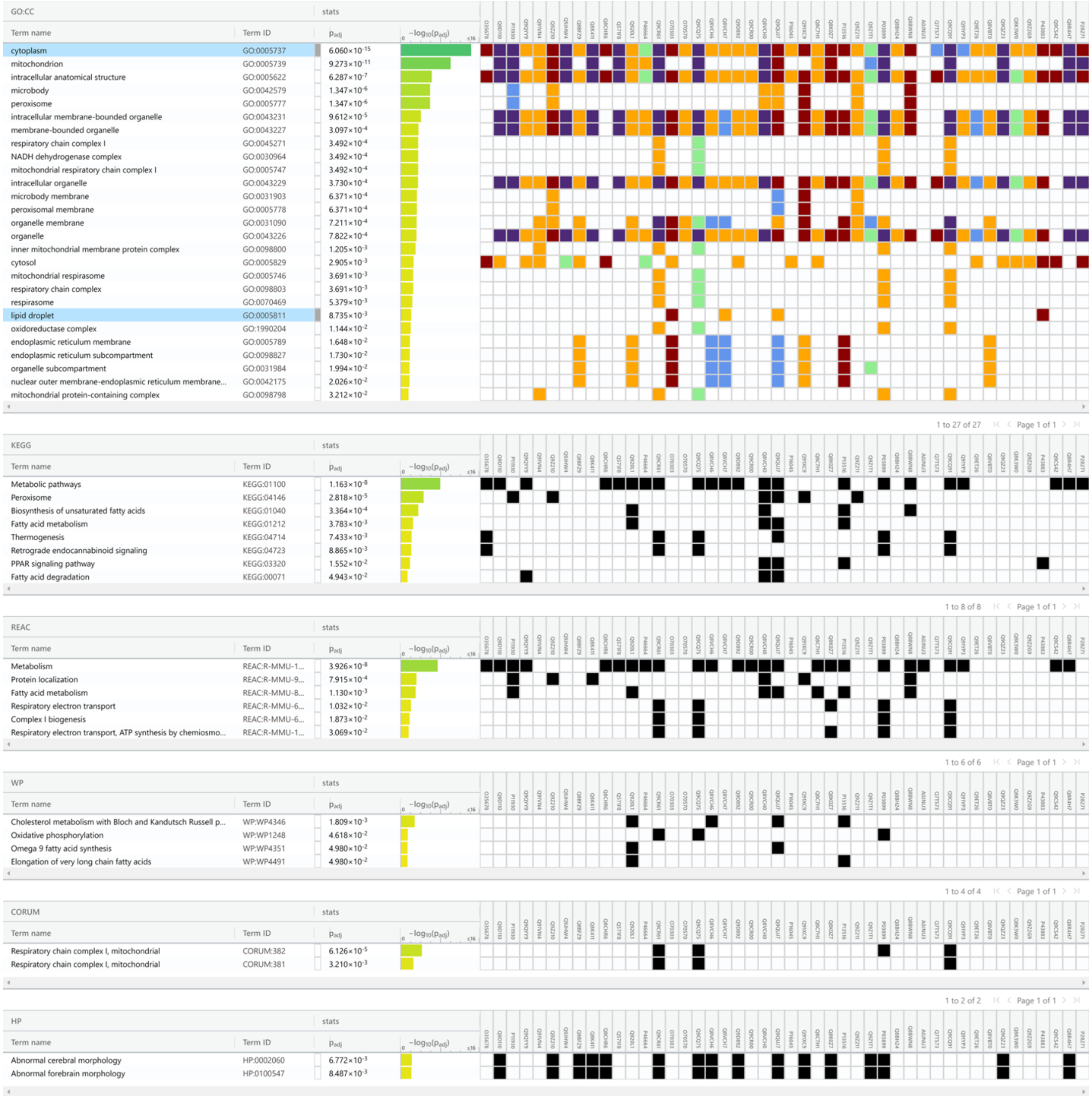

Fig. S5 (continued). (B) Gene Ontology Cellular Component (GO:CC), Kyoto Encyclopedia of Genes and Genomes (KEGG), Reactome (REAC), WikiPathways (WP), Corum, and Human Phenotype Ontology (HP). Functional annotation was performed by using the gProfiler tool.

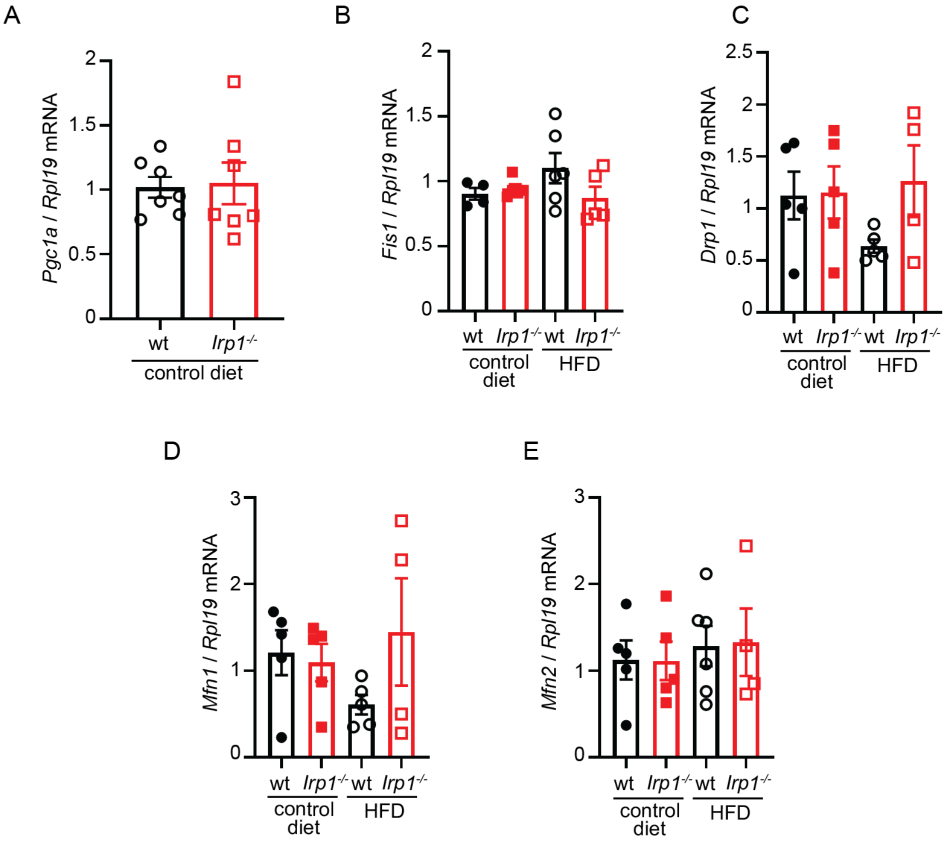

Fig. S6. IRP1 deficiency does not affect expression of genes involved in mitochondrial biogenesis, fission, or fusion. RNA was prepared from livers of mice described in Fig. 3 and used for qPCR analysis of: (A) *Pgc1a* mRNA; (B) *Fis1* mRNA; (C) *Drp1* mRNA; (D) *Mfn1* mRNA; and (E) *Mfn2* mRNA. Quantitative data are presented as the mean±SEM. Statistical analysis was performed by ANOVA with Tukey's multiple comparisons test or with the Student's t test; no significant differences were found.

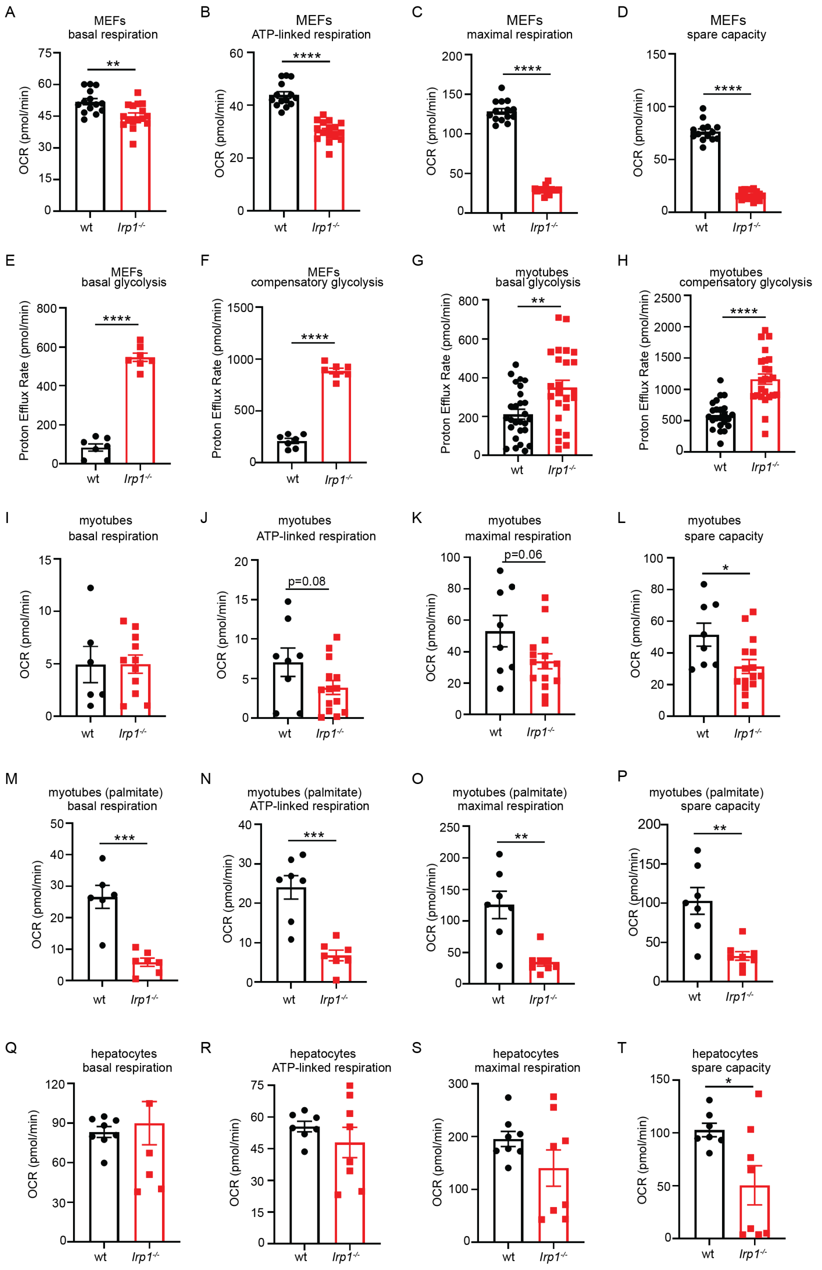

Fig. S7. Quantification of mitochondrial respiration and glycolysis in *Irp1*$^{-/-}$ and wild type mouse embryonic fibroblasts (MEFs), primary differentiated myotubes and primary hepatocytes. Original Seahorse data are described in Fig. 4. (A) Basal respiration (MEFs). (B) ATP-linked respiration (MEFs). (C) Maximal respiration (MEFs). (D) Spare capacity (MEFs). (E) Basal glycolysis (MEFs). (F) Compensatory glycolysis (MEFs). (G) Basal glycolysis (myotubes). (H) Compensatory glycolysis (myotubes). (I) Basal respiration (myotubes). (J) ATP-linked respiration (myotubes). (K) Maximal respiration (myotubes). (L) Spare capacity (myotubes). (M) Basal respiration with palmitate (myotubes). (N) ATP-linked respiration with palmitate (myotubes). (O) Maximal respiration with palmitate (myotubes). (P) Spare capacity with palmitate (myotubes). (Q) Basal respiration (hepatocytes). (R) ATP-linked respiration (hepatocytes). (S) Maximal respiration (hepatocytes). (T) Spare capacity (hepatocytes). Data are presented as the mean ± SEM. Statistical analysis was performed with the Student's t test. Significant differences are indicated by * ($p<0.05$), ** ($p<0.01$), *** ($p<0.001$), or **** ($p<0.0001$), respectively.

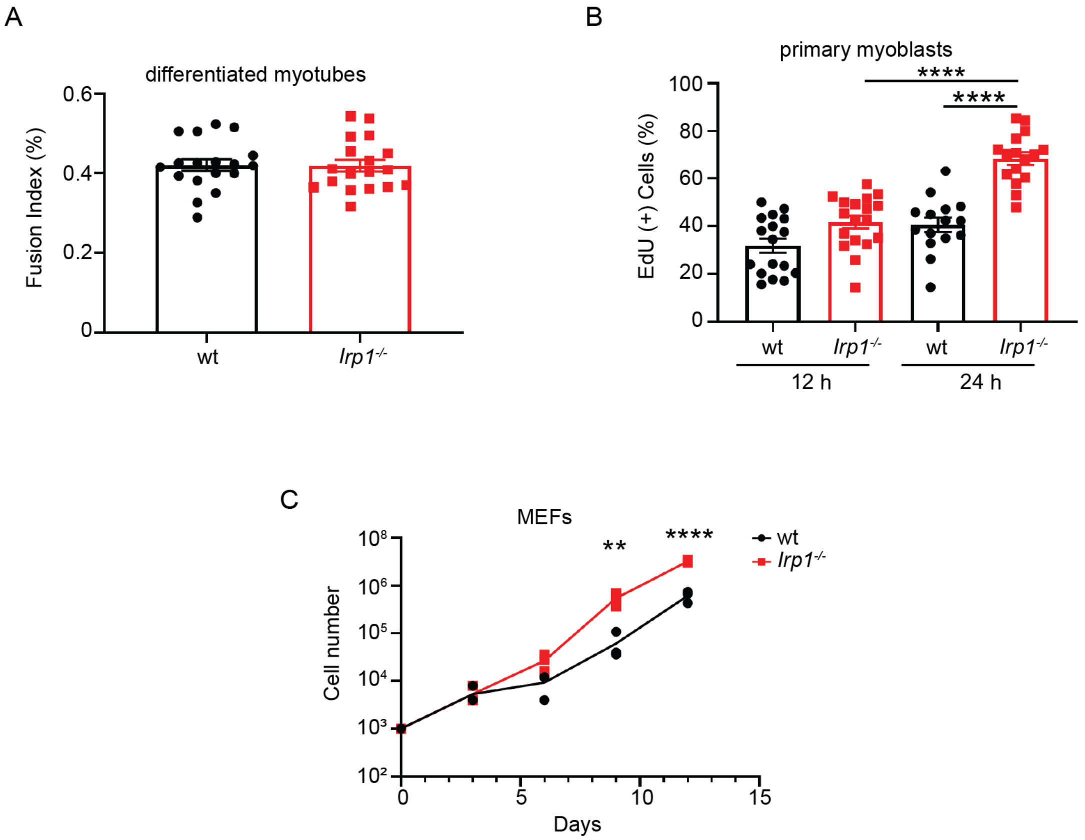

Fig. S8. IRP1 deficiency increases cell proliferation. (A) Fusion index in differentiated myotubes from *Irp1$^{-/-}$* and wild type mice. Fusion index was calculated as the percentage of myonuclei of the total number of nuclei per field of view. (B) EdU proliferation assay with primary myoblasts grown for 12 h or 24 h. (C) Proliferation assay with MEFs grown for 12 days. Data are presented as the mean ± SEM. Statistical analysis was performed by ANOVA with Tukey's multiple comparisons test. Significant differences are indicated by ** ($p<0.01$) or **** ($p<0.0001$).

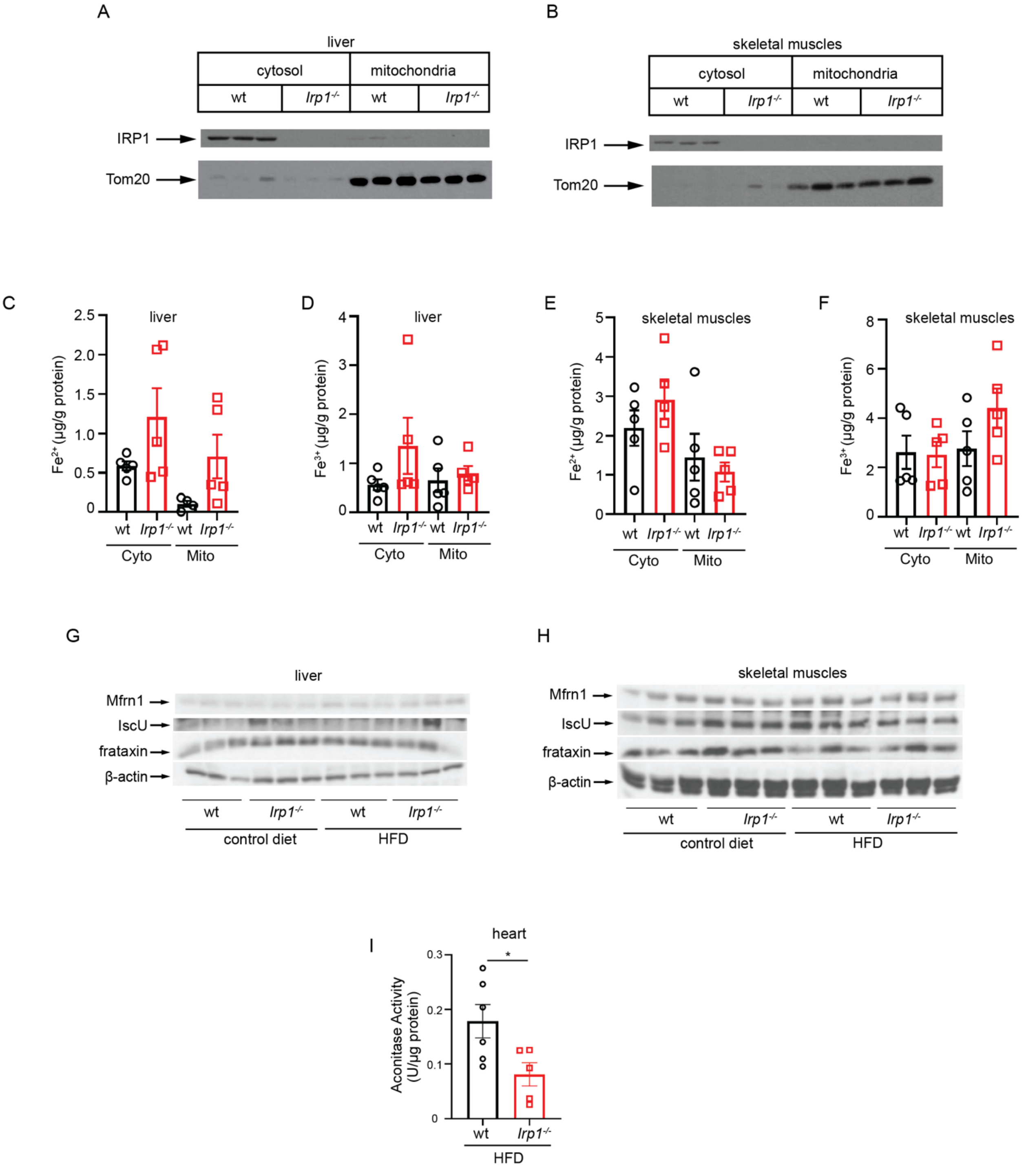

Fig. S9. Subcellular iron speciation analysis and Western blot analysis of IscU and frataxin in the liver and skeletal muscles. Liver and skeletal muscles from *Irp1*$^{-/-}$ and wild type mice described in Fig. 5A-F were used for subcellular fractionation, iron speciation analysis or Western blotting. (A-B) Western blot analysis of IRP1 (cytosolic marker) and Tom20 (mitochondrial marker) in cytosolic and mitochondrial fractions of liver (A) and skeletal muscles (B). (C-D) Quantification of liver cytosolic and mitochondrial Fe^{2+} (C) and Fe^{3+} (D). (E-F) Quantification of skeletal muscle cytosolic and mitochondrial Fe^{2+} (E) and Fe^{3+} (F). (G) Western blot analysis of IscU, frataxin and β-actin in whole liver extracts. (H) Western blot analysis of IscU, frataxin and β-actin in whole skeletal muscle extracts. (I) Aconitase enzymatic assay in mitochondrial heart extracts

from *Irp1*$^{-/-}$ and wild type mice on HFD. Quantitative data are presented as the mean±SEM. Statistical analysis was performed by ANOVA with Tukey's multiple comparisons test; comparisons between two groups were done with the Student's t test. Significant differences are indicated by * ($p<0.05$).

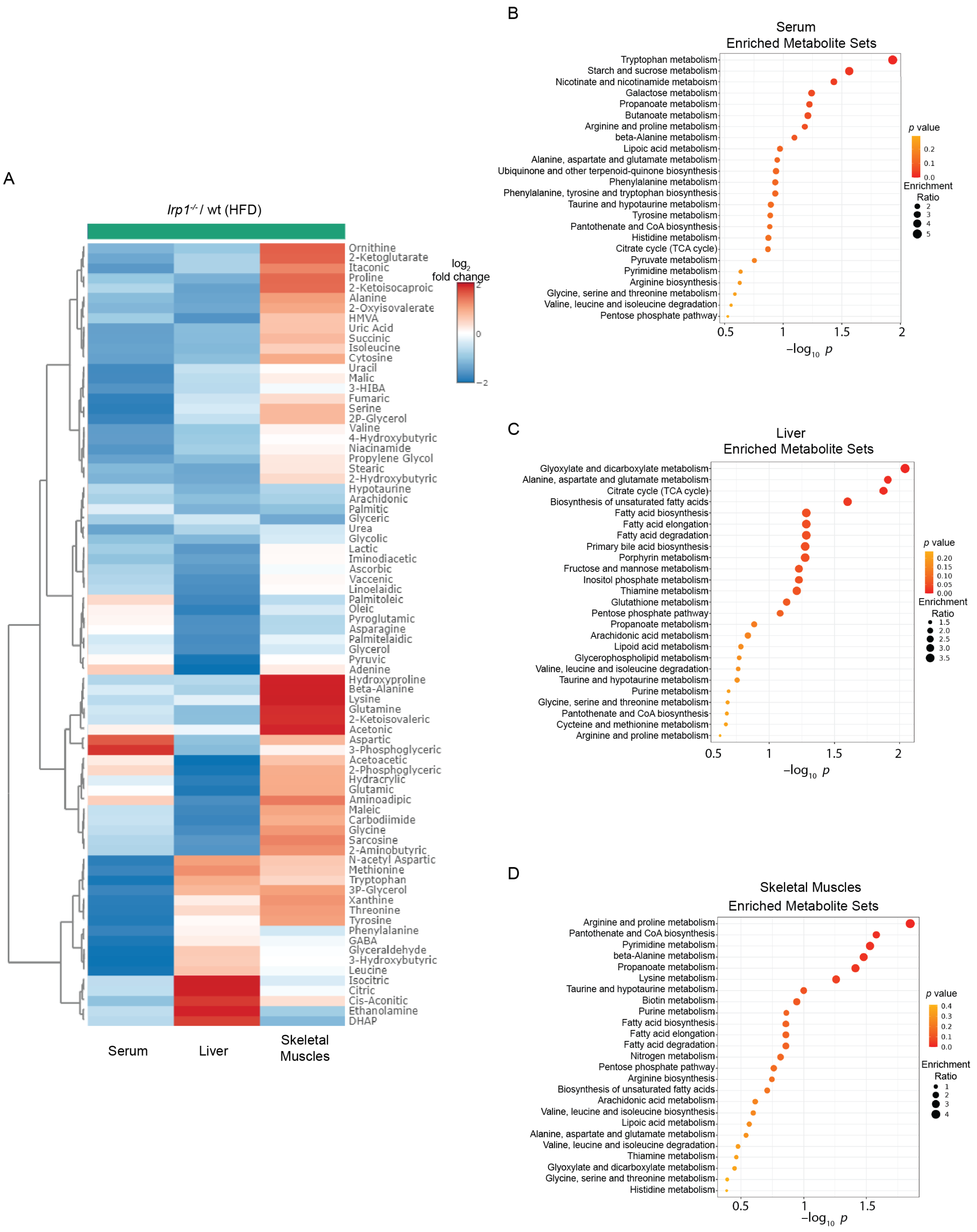

Fig. S10. IRP1 deficiency triggers metabolic reprogramming in mice fed a high-fat diet (HFD). *Irp1*$^{-/-}$ and wild type mice described in Fig. 6 were used for metabolomics analysis in serum, liver and skeletal muscles. (A) Heatmap of all detected metabolites (*Irp1*$^{-/-}$ vs wild type). (B) Pathway enrichment analysis for serum metabolites. (C) Pathway enrichment analysis for liver metabolites. (D) Pathway enrichment analysis for skeletal muscle metabolites. In (A), $\log_2$ fold changes are indicated on the right. In (B-D), p values and enrichment ratios are indicated on the right.

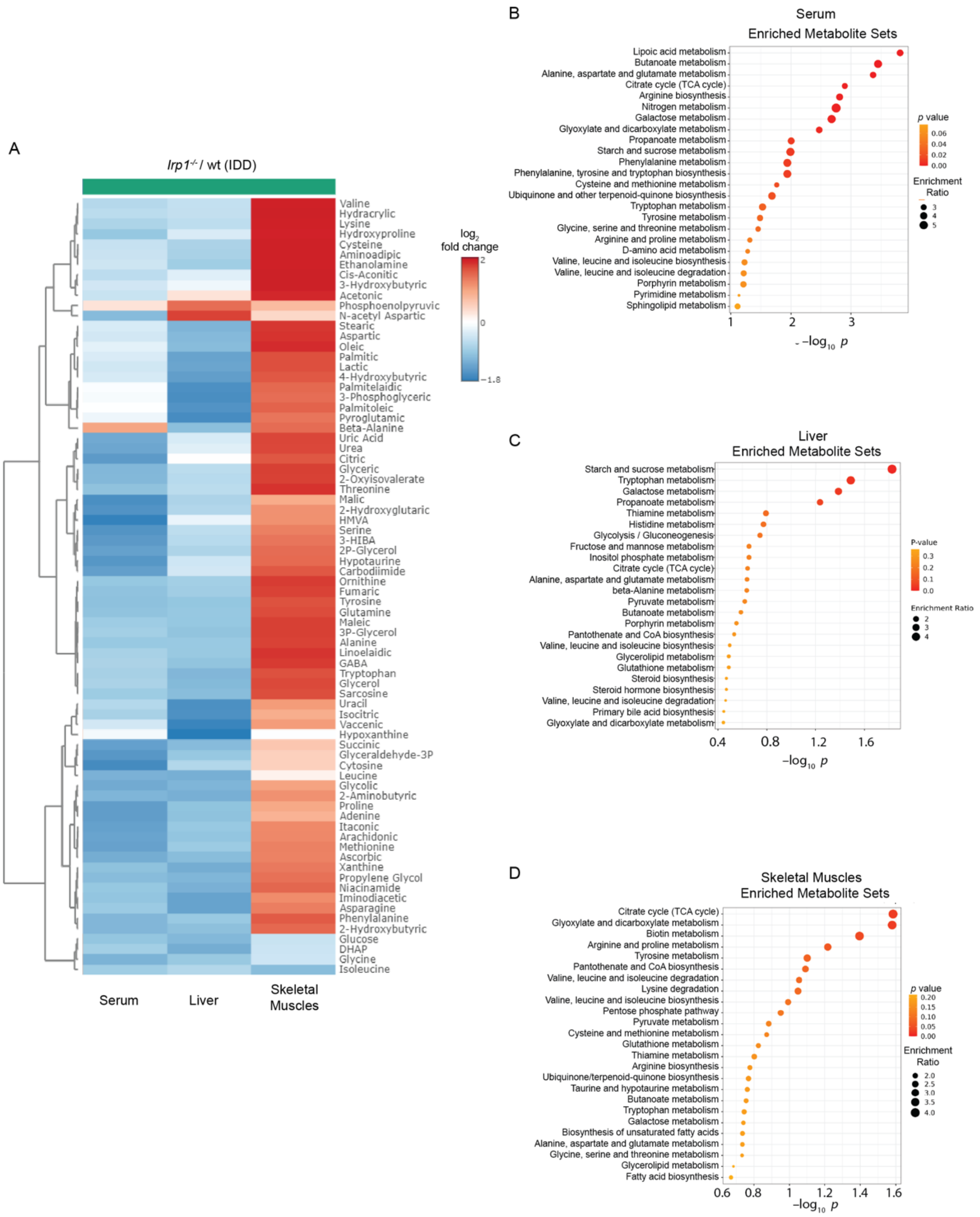

Fig. S11. IRP1 deficiency triggers metabolic reprogramming in mice fed an iron-deficient diet (IDD). *Irp1*$^{-/-}$ and wild type mice described in Fig. 6 were used for metabolomics analysis in serum, liver and skeletal muscles. (A) Heatmap of all detected metabolites (*Irp1*$^{-/-}$ vs wild type). (B) Pathway enrichment analysis for serum metabolites. (C) Pathway enrichment analysis for liver metabolites. (D) Pathway enrichment analysis for skeletal muscle metabolites. In (A), $\log_2$ fold changes are indicated on the right. In (B-D), p values and enrichment ratios are indicated on the right.

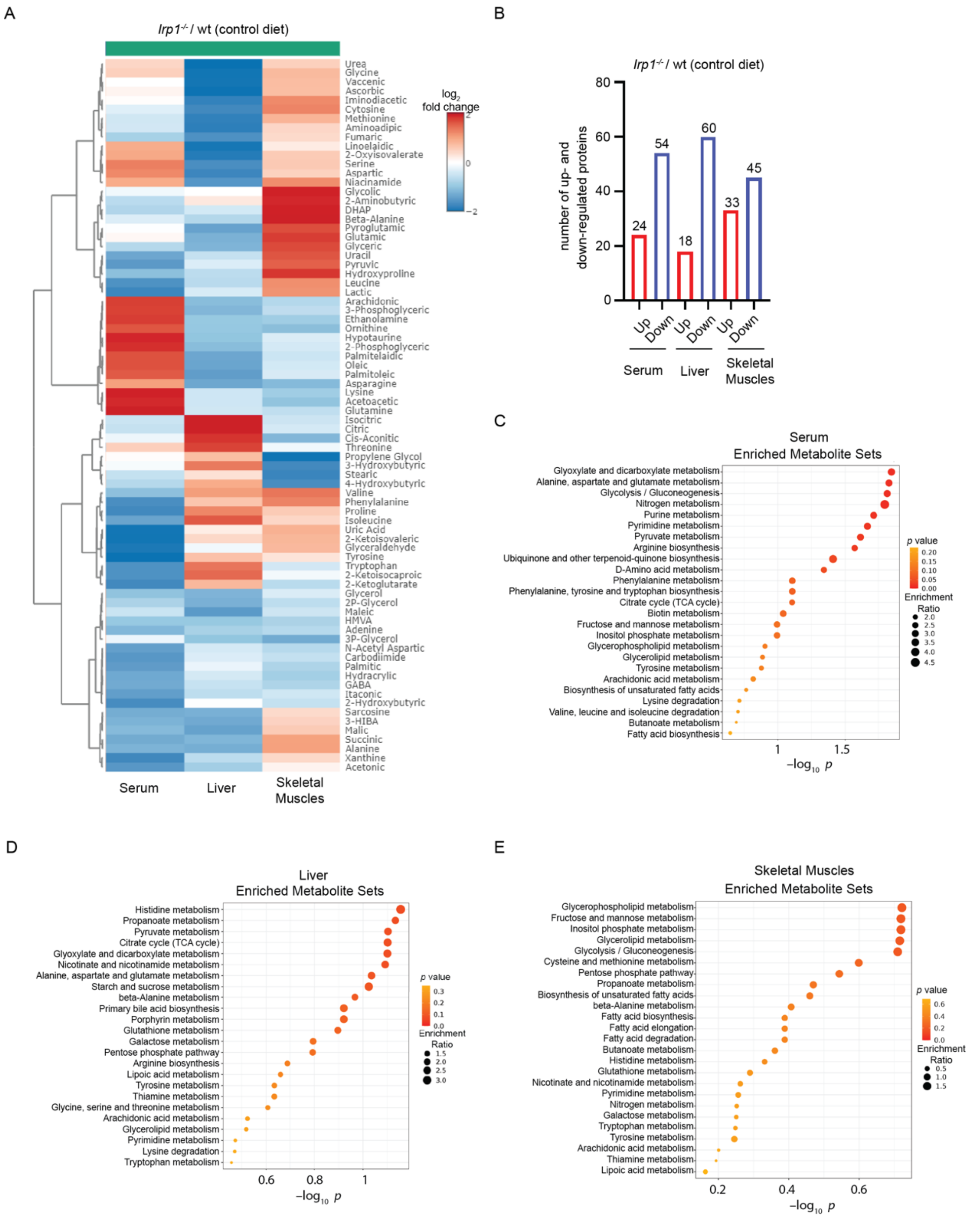

Fig. S12. IRP1 deficiency triggers metabolic reprogramming in mice on a standard diet. 5-weeks old male *Irp1*$^{-/-}$ mice and wild type littermates (n=4-5 per experimental group) were provided a standard diet (Teklad Global 18% protein 2918) for 10 weeks. At the endpoint, the mice were euthanized, and serum, liver and skeletal muscle samples were collected and processed for targeted metabolomics analysis. (A) Heatmap of all detected metabolites (*Irp1*$^{-/-}$ vs wild type). (B) Number of up- and down-regulated metabolites (*Irp1*$^{-/-}$ vs wild type). (C) Pathway enrichment analysis for serum metabolites. (D) Pathway enrichment analysis for liver metabolites. (E) Pathway enrichment analysis for skeletal muscle metabolites. In (C-E), p values and enrichment ratios are indicated on the right.

Table S1. Complete blood count (CBC) of male *Irp1*$^{-/-}$ mice and wild type littermates after HFD feeding for 10 weeks. Values are followed by 95% confident interval.

	wild type	*Irp1*$^{-/-}$	p-value	sample size
WBC ($10^3/mm^3$)	5.57 (3.11, 8.03)	3.04 (1.09, 4.98)	0.144	6
LYM ($10^3/mm^3$)	3.48 (2.43, 4.53)	2.73 (0.76, 4.71)	0.495	3-5
MON ($10^3/mm^3$)	0.38 (0.27, 0.49)	0.30 (0.07, 0.53)	0.512	3-5
GRA ($10^3/mm^3$)	2.72 (1.61, 3.83)	1.87 (1.74, 2.00)	0.303	3-5
LYM%	54.32 (46.35, 62.29)	52.33 (31.88, 72.78)	0.839	3-5
MON%	6.56 (5.48, 7.64)	6.83 (5.56, 8.11)	0.765	3-5
GRA%	39.12 (30.22, 48.02)	40.83 (19.15, 62.52)	0.871	3-5
EOS%	2.58 (1.80, 3.36)	2.53 (1.65, 3.41)	0.943	6
RBC ($10^6/mm^3$)	8.98 (8.54, 9.42)	8.33 (7.89, 8.76)	0.067	6
HGB (g/dL)	14.22 (13.34, 15.10)	12.88 (12.14, 13.61)	*0.045	6
HCT%	41.62 (39.55, 43.69)	37.65 (35.64, 39.66)	*0.023	6
MCV (μm^3)	46.5 (46.06, 46.94)	45.17 (44.23, 46.10)	*0.030	6
MCH (pg)	15.82 (15.57, 16.07)	15.47 (15.20, 15.73)	0.084	6
MCHC (g/dL)	34.14 (33.56, 34.71)	34.20 (33.64, 34.76)	0.876	6
RDW%	14.62 (14.16, 15.07)	14.32 (13.74, 14.90)	0.440	6
PLT ($10^3/mm^3$)	662 (537, 787)	750 (636, 864)	0.332	6
MPV (μm^3)	6.57 (5.93, 7.20)	6.67 (6.34, 6.99)	0.790	6

Supplemental references

1. Divakaruni AS, Jastroch M. A practical guide for the analysis, standardization and interpretation of oxygen consumption measurements. Nat Metab. 2022;4(8):978-94.
2. Solovyev N, Vinceti M, Grill P, Mandrioli J, Michalke B. Redox speciation of iron, manganese, and copper in cerebrospinal fluid by strong cation exchange chromatography - sector field inductively coupled plasma mass spectrometry. Anal Chim Acta. 2017;973:25-33.
3. Jenkins B, Ronis M, Koulman A. LC-MS Lipidomics: Exploiting a Simple High-Throughput Method for the Comprehensive Extraction of Lipids in a Ruminant Fat Dose-Response Study. Metabolites. 2020;10(7).
4. Eng JK, McCormack AL, Yates JR. An approach to correlate tandem mass spectral data of peptides with amino acid sequences in a protein database. J Am Soc Mass Spectrom. 1994;5(11):976-89.
5. Reimand J, Arak T, Adler P, Kolberg L, Reisberg S, Peterson H, et al. g:Profiler-a web server for functional interpretation of gene lists (2016 update). Nucleic Acids Res. 2016;44(W1):W83-9.
6. Daba A, Gkouvatsos K, Sebastiani G, Pantopoulos K. Differences in activation of mouse hepcidin by dietary iron and parenterally administered iron dextran: compartmentalization is critical for iron sensing. J Mol Med (Berl). 2013;91(1):95-102.
7. Fillebeen C, Chahine D, Caltagirone A, Segal P, Pantopoulos K. A phosphomimetic mutation at Ser-138 renders iron regulatory protein 1 sensitive to iron-dependent degradation. Mol Cell Biol. 2003;23(19):6973-81.
8. Galy B, Ferring D, Hentze MW. Generation of conditional alleles of the murineiron regulatory protein (IRP)-1 and -2 genes. genesis. 2005;43(4):181-8.
