# Supplementary material for: IRP1 deficiency alters mitochondrial metabolism and protects against metabolic syndrome pathologies
Source: JCI Insight. 2026 Jan 6;11(4):e183247. doi: 10.1172/jci.insight.183247 (PMC12956002; doi:10.1172/jci.insight.183247)

Fig 3E

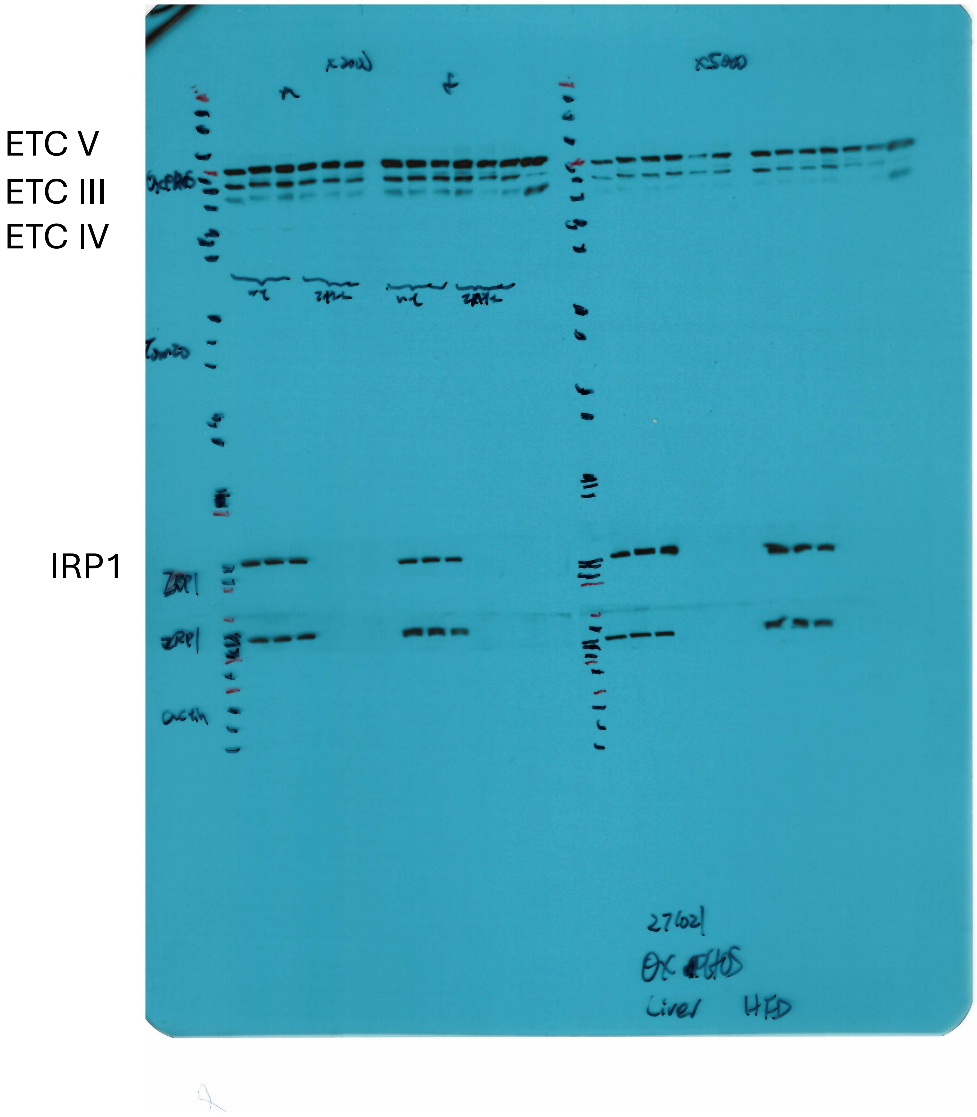

Fig 3E

ETC II

ETC I

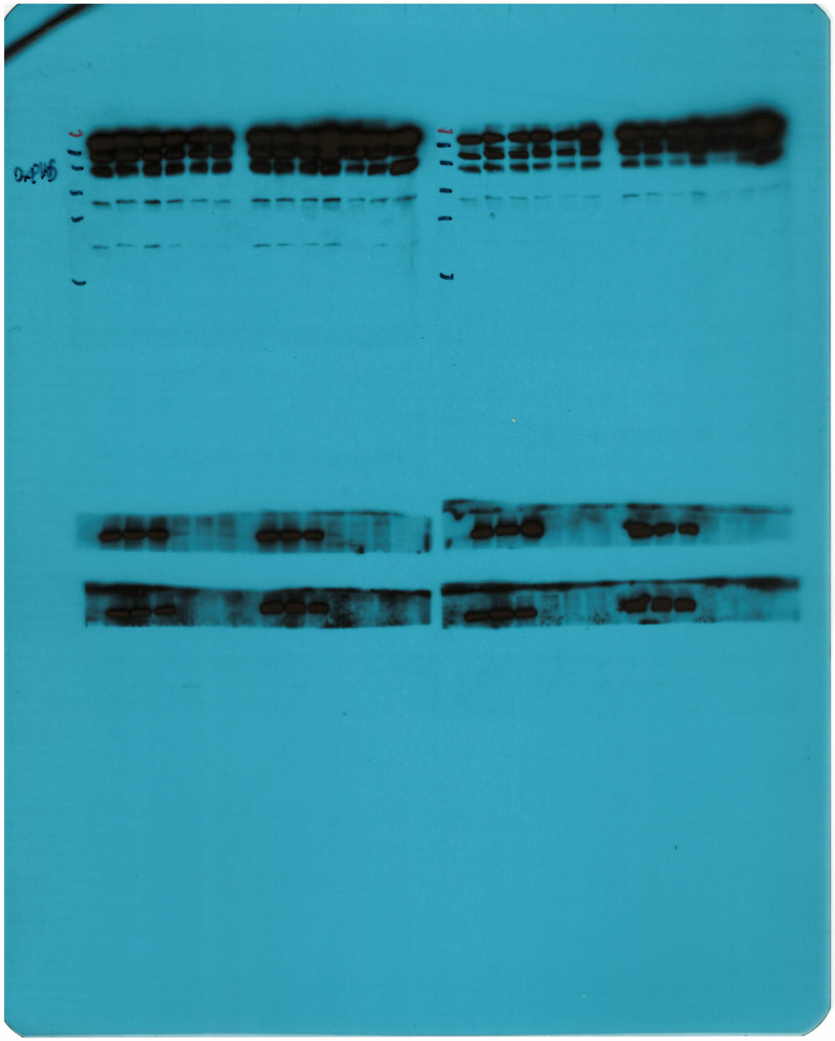

Fig 3E

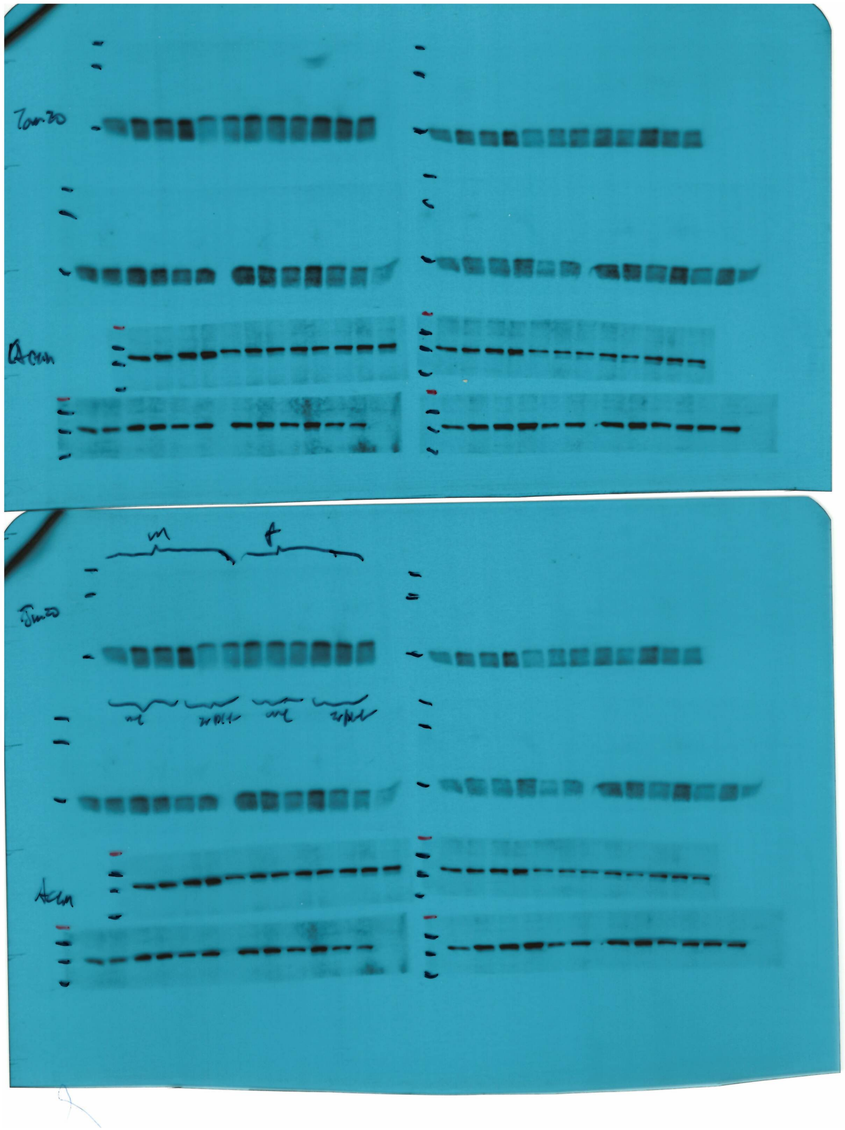

Tomm20

Actin

Fig 5A

IRP1

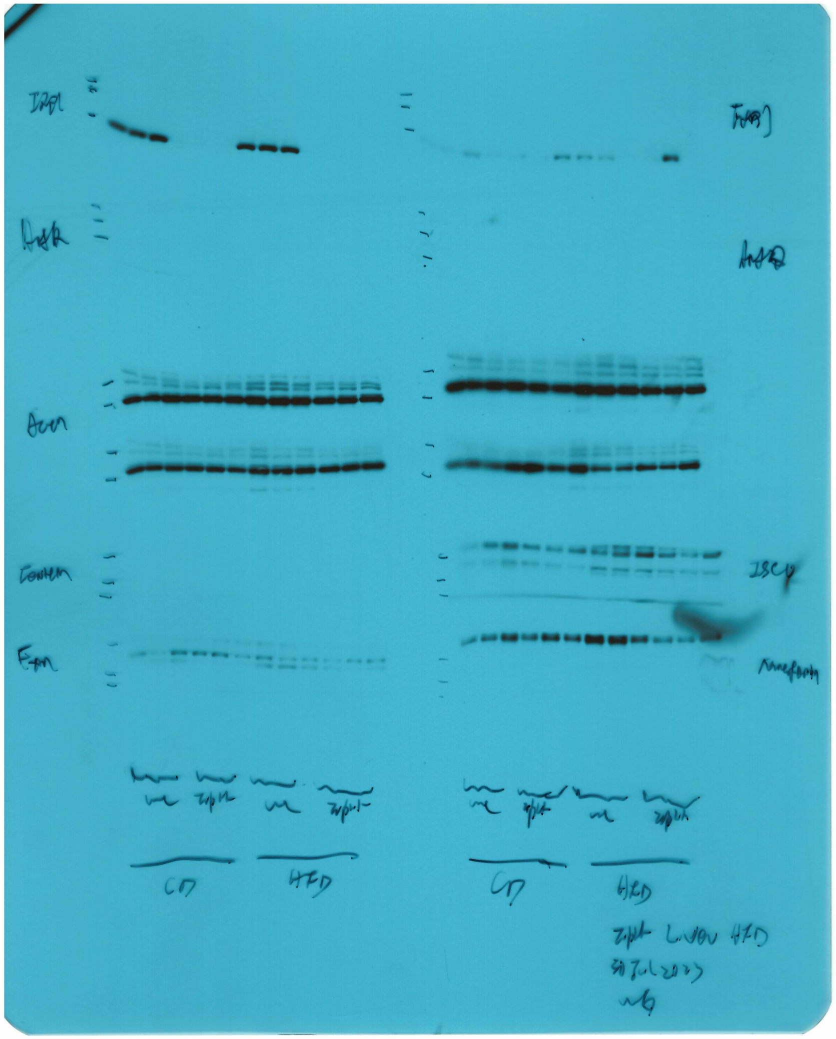

Fig 5A

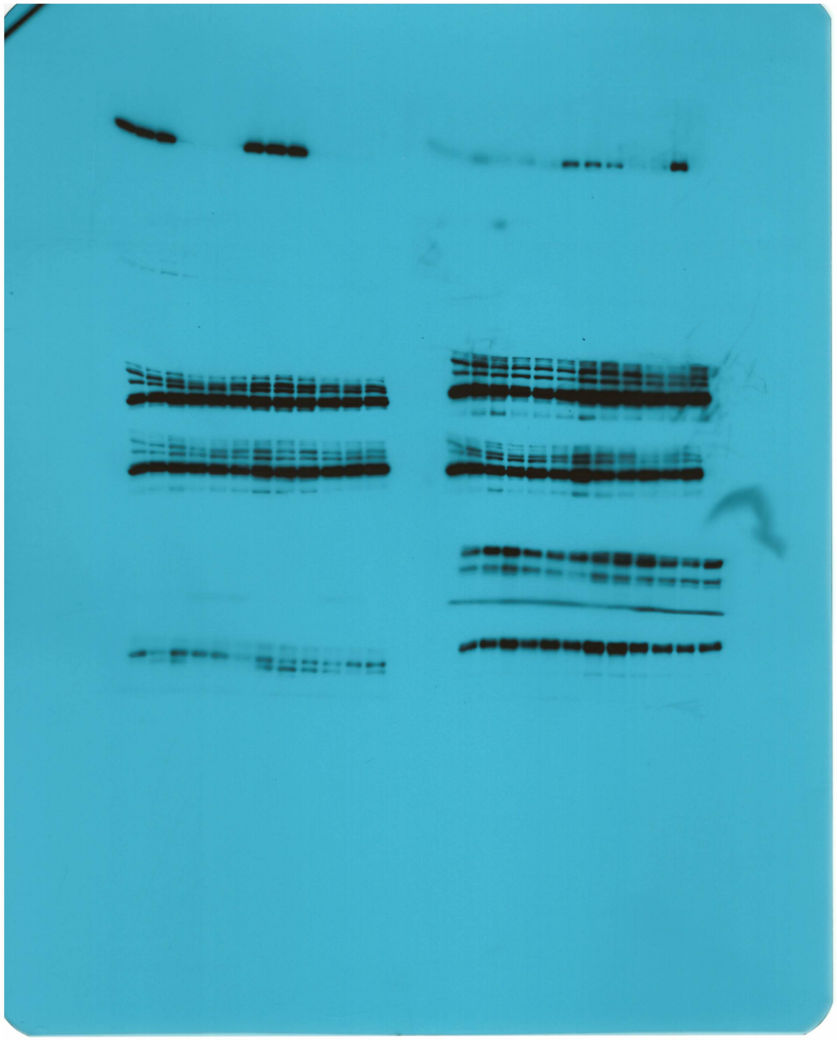

TFR1

Fig 5A

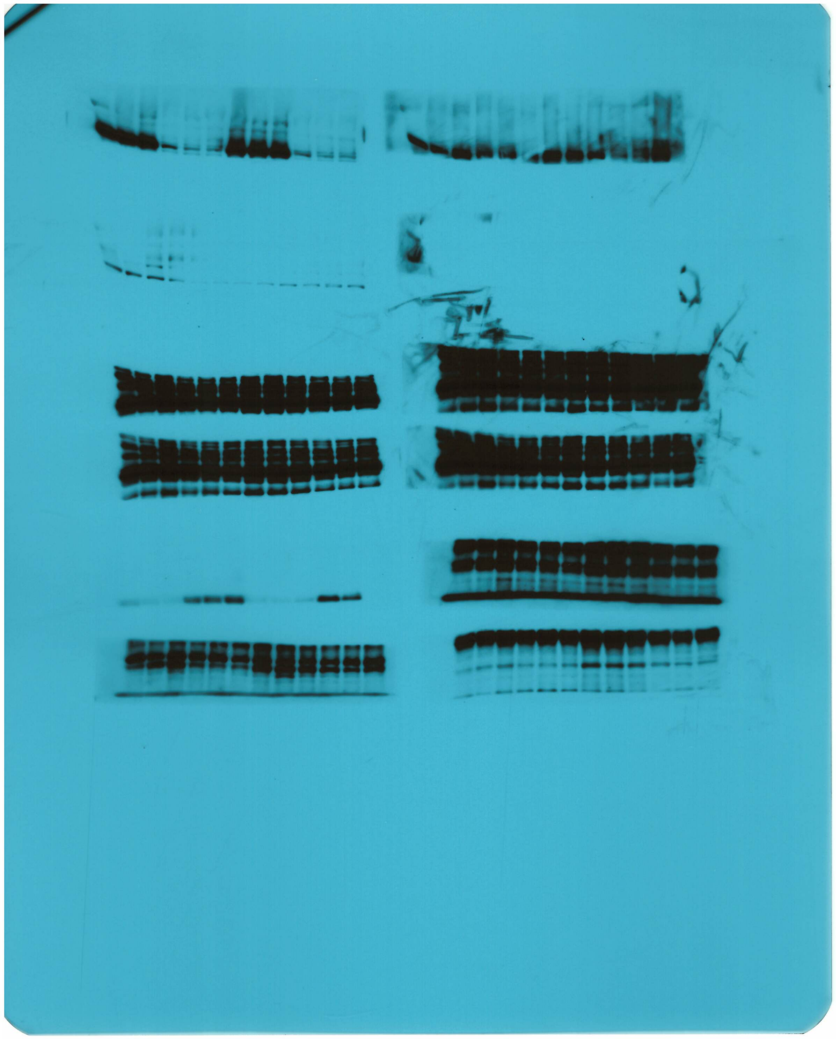

Ferritin

Fig 5A

Actin

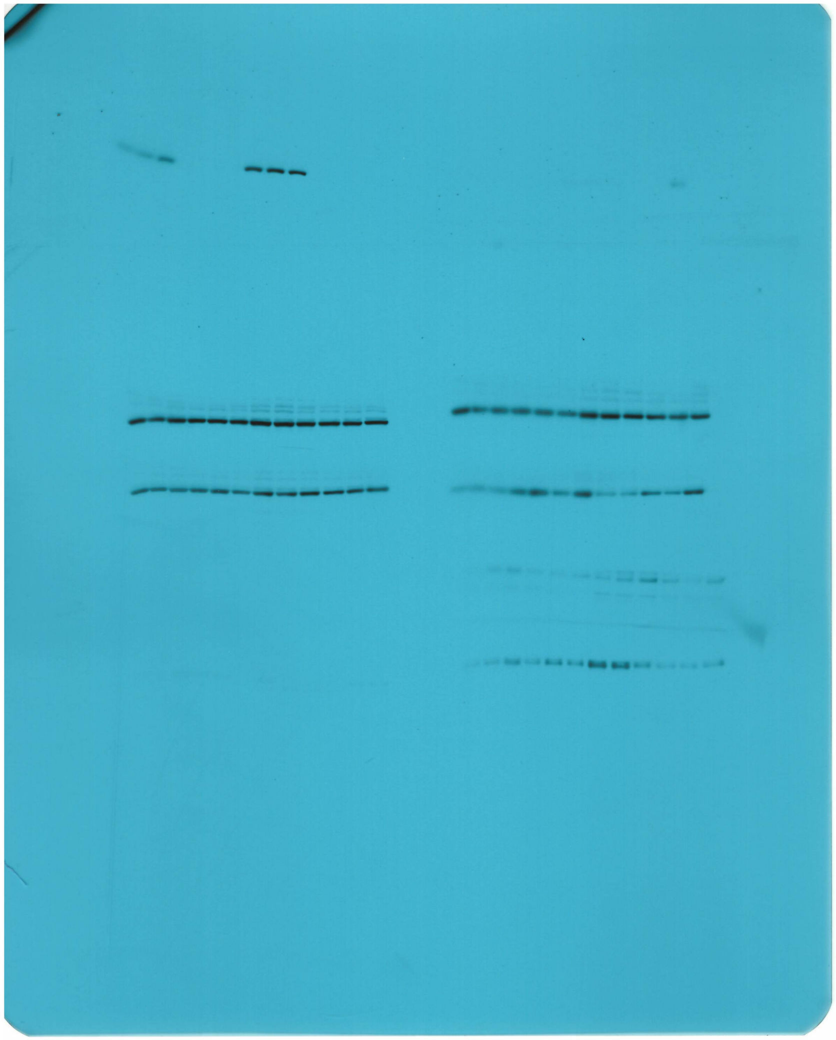

Fig 5B

IRP1

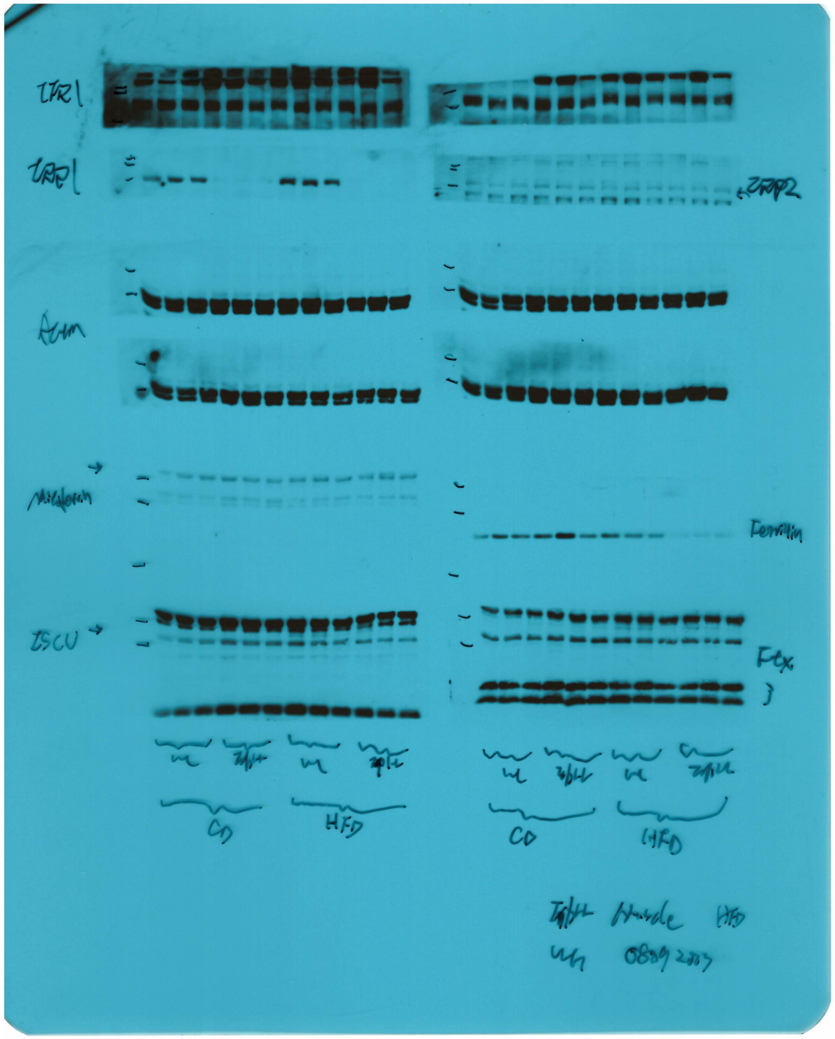

Ferritin

Fig 5B

Actin

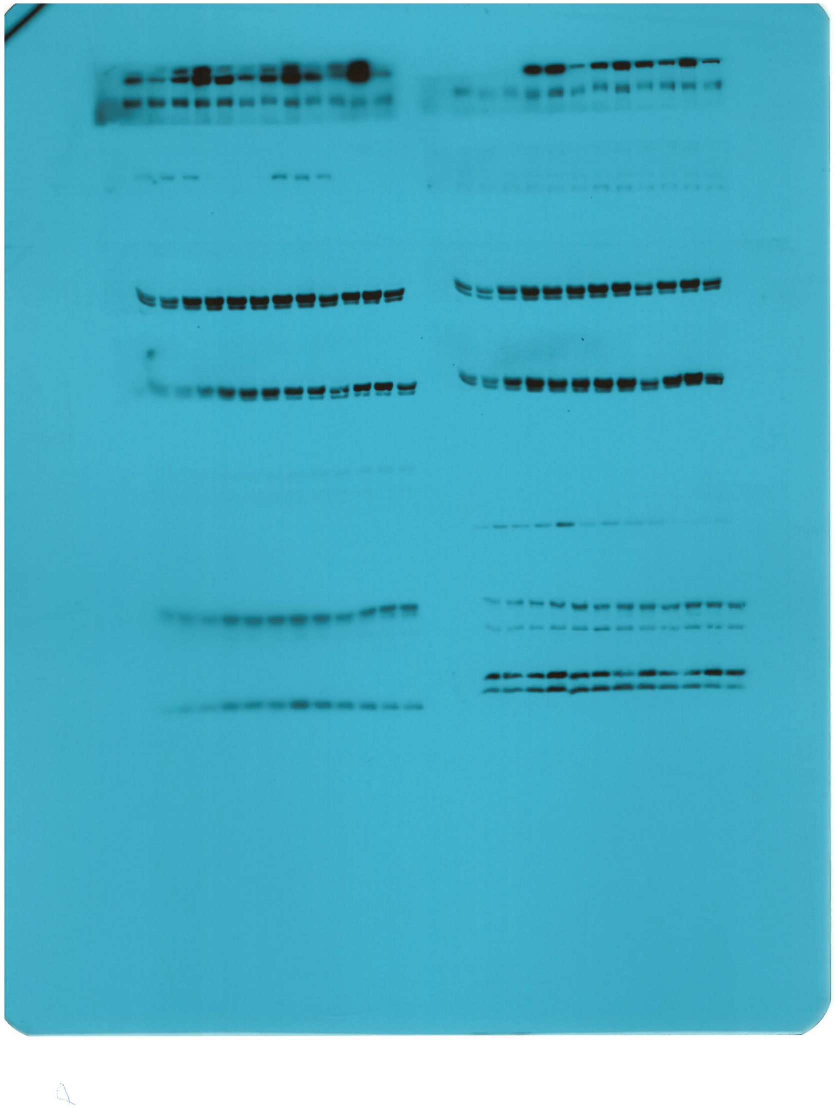

Fig 5B

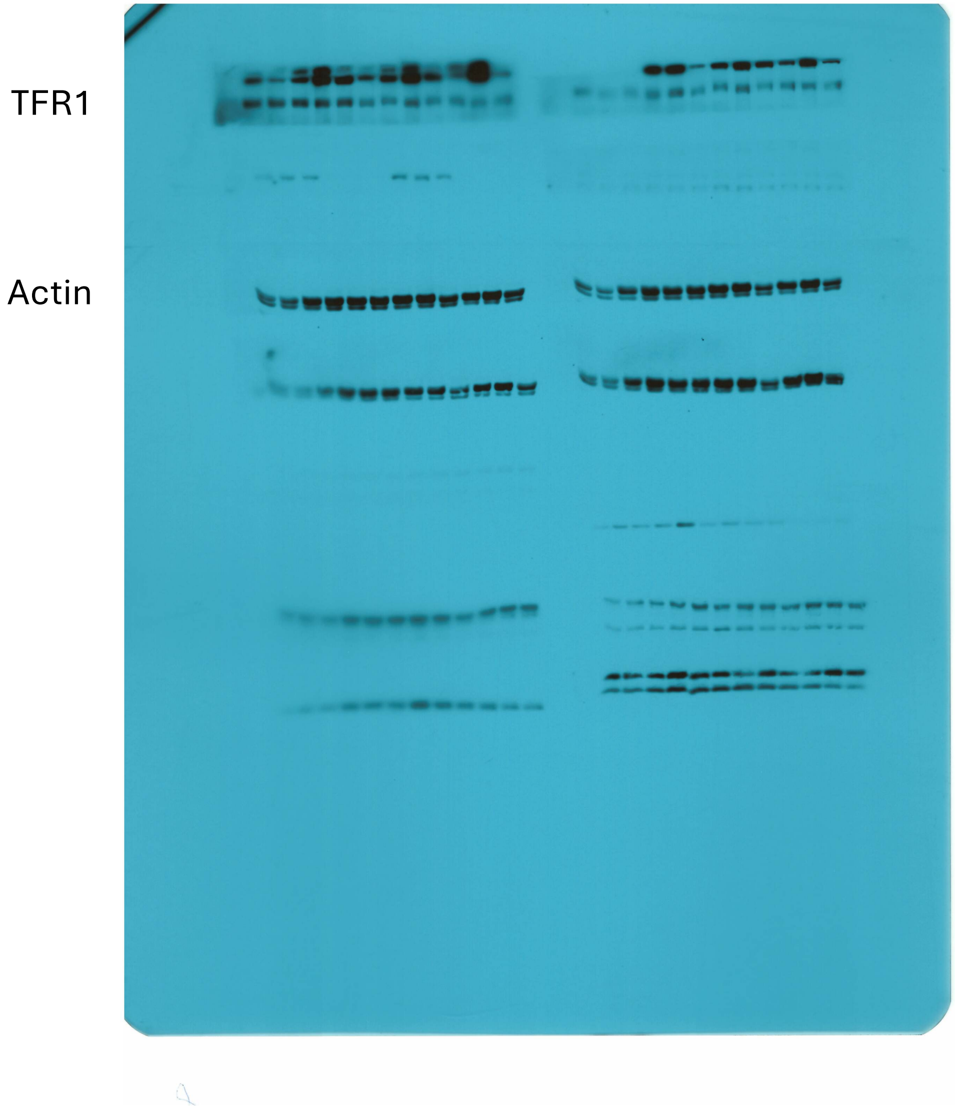

Fig 7A

pAKT

IRP1

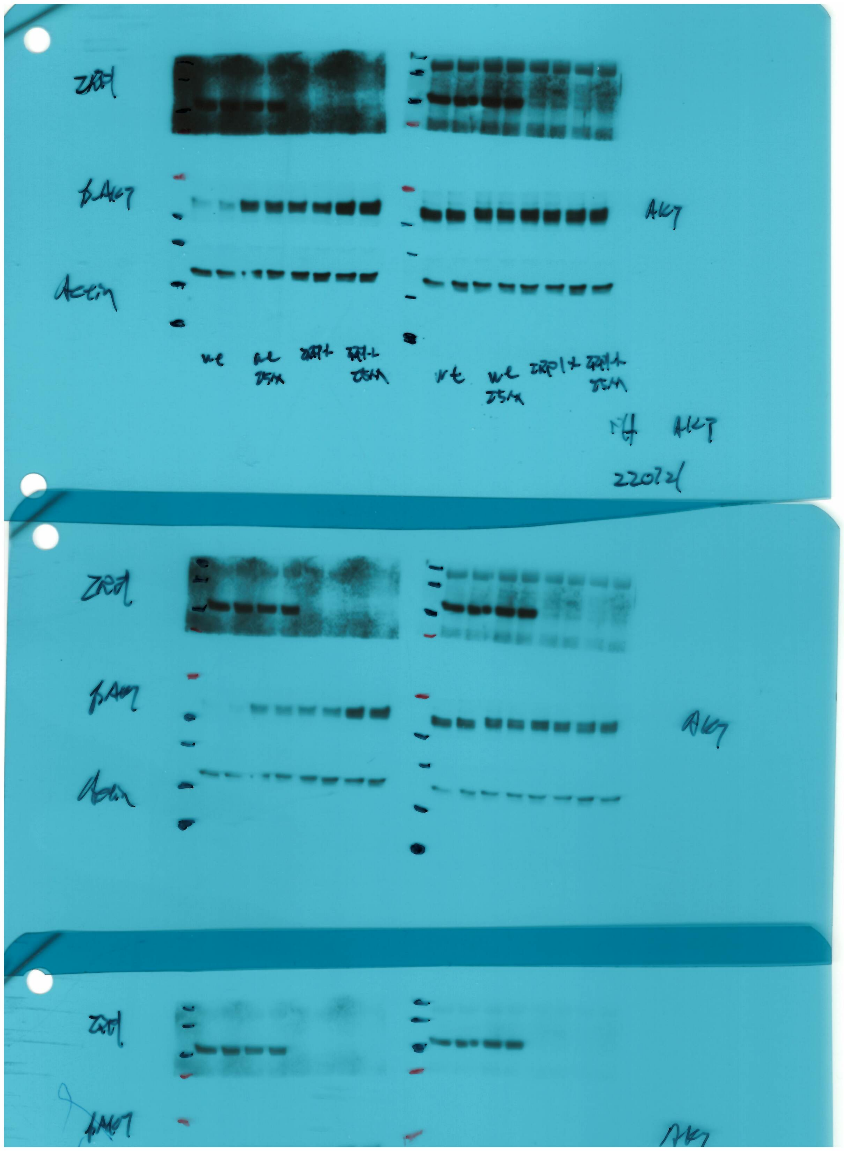

Actin

AKT

Fig 7B

AKT

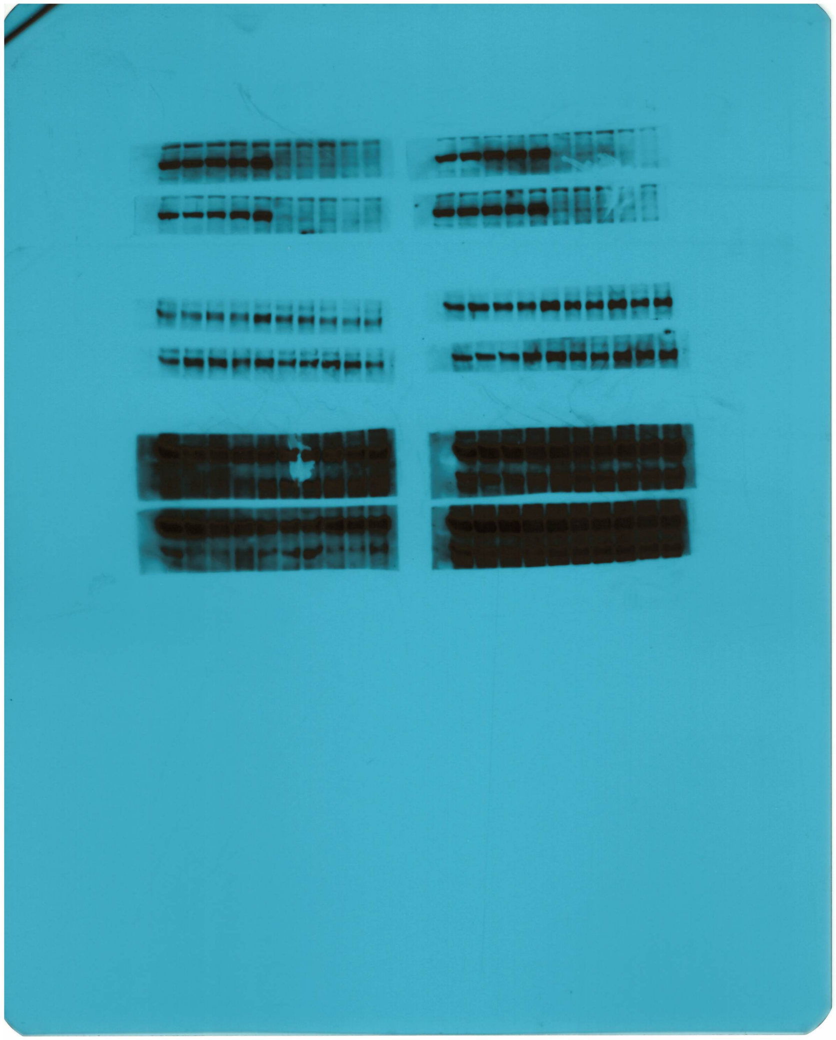

Fig 7B

IRP1

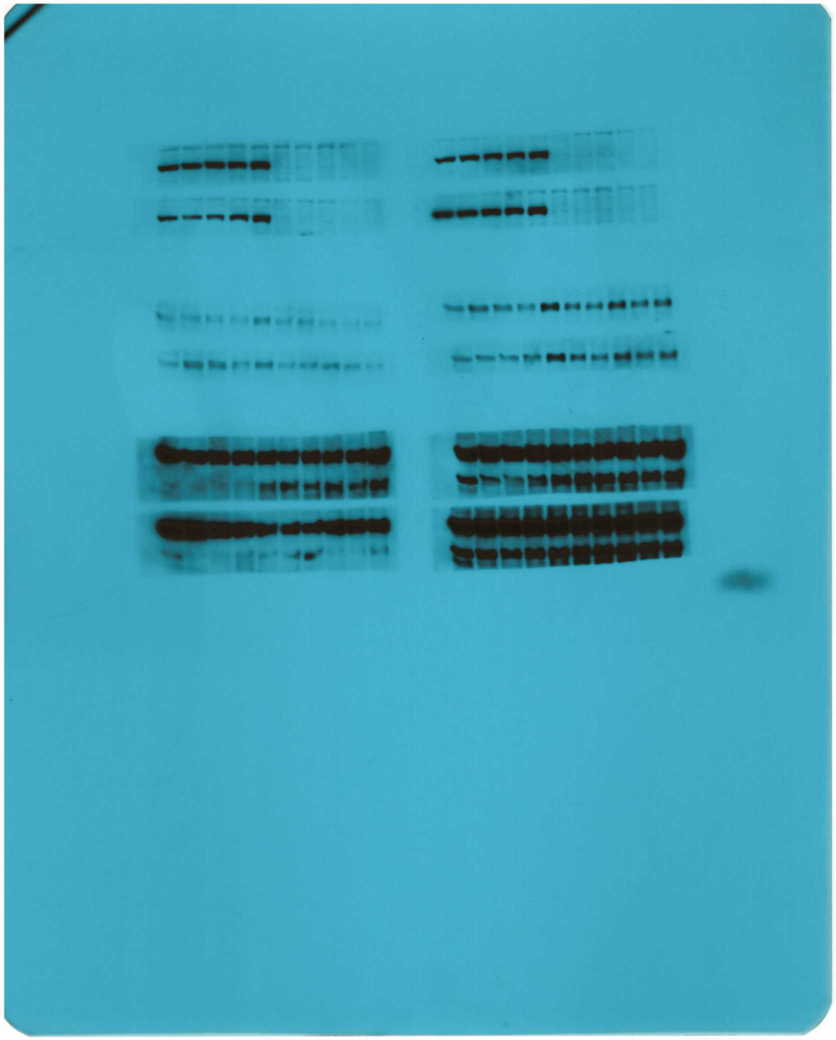

pAKT

Fig 7B

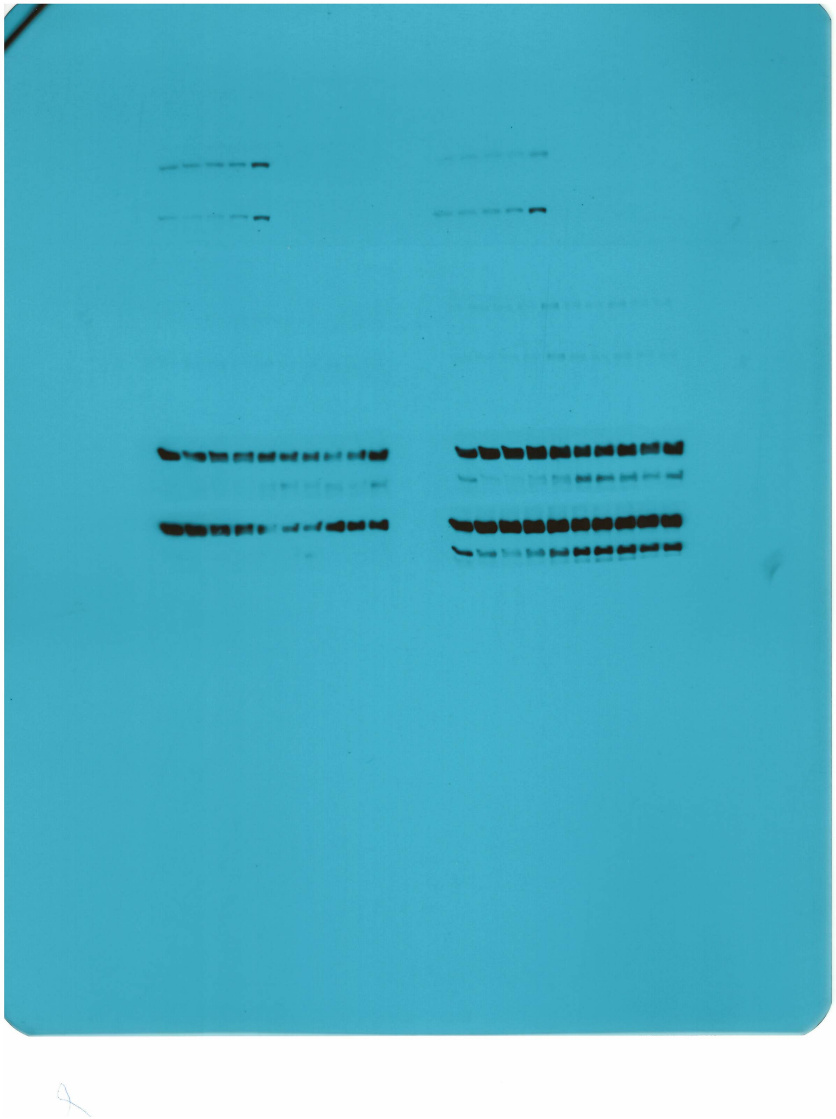

Actin

Fig 7C

AKT

pAKT

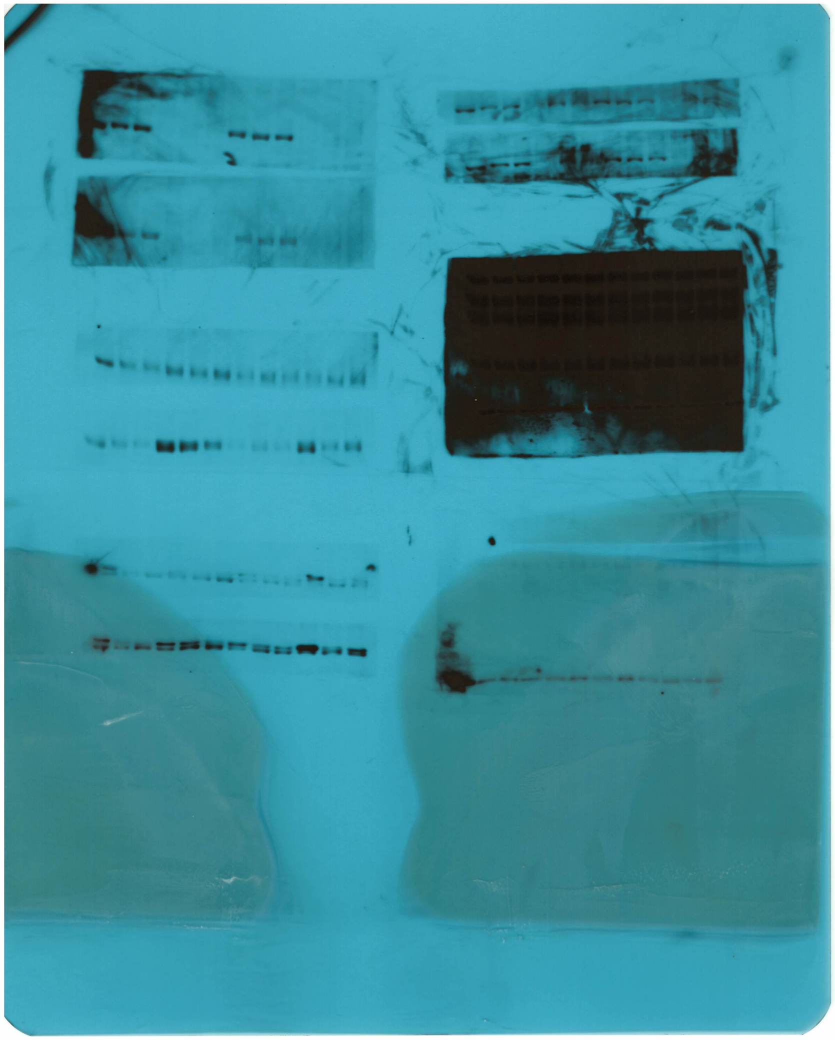

Fig 7C

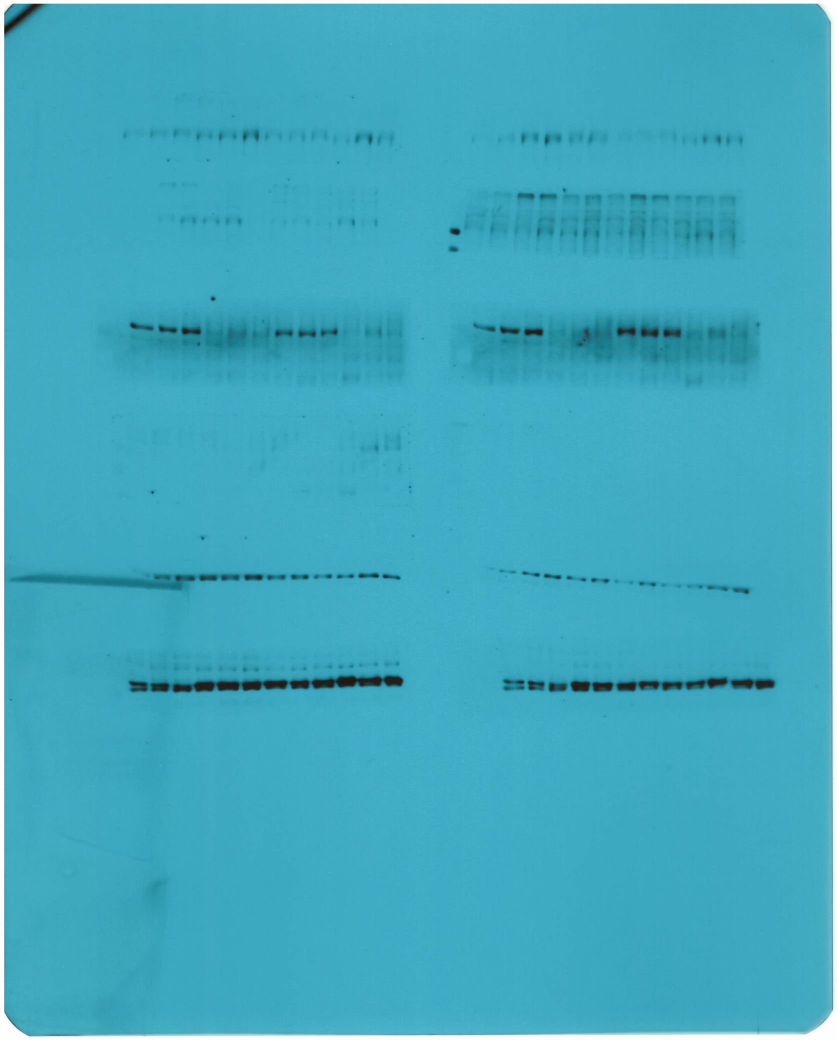

Actin

Fig 7C

IRP1

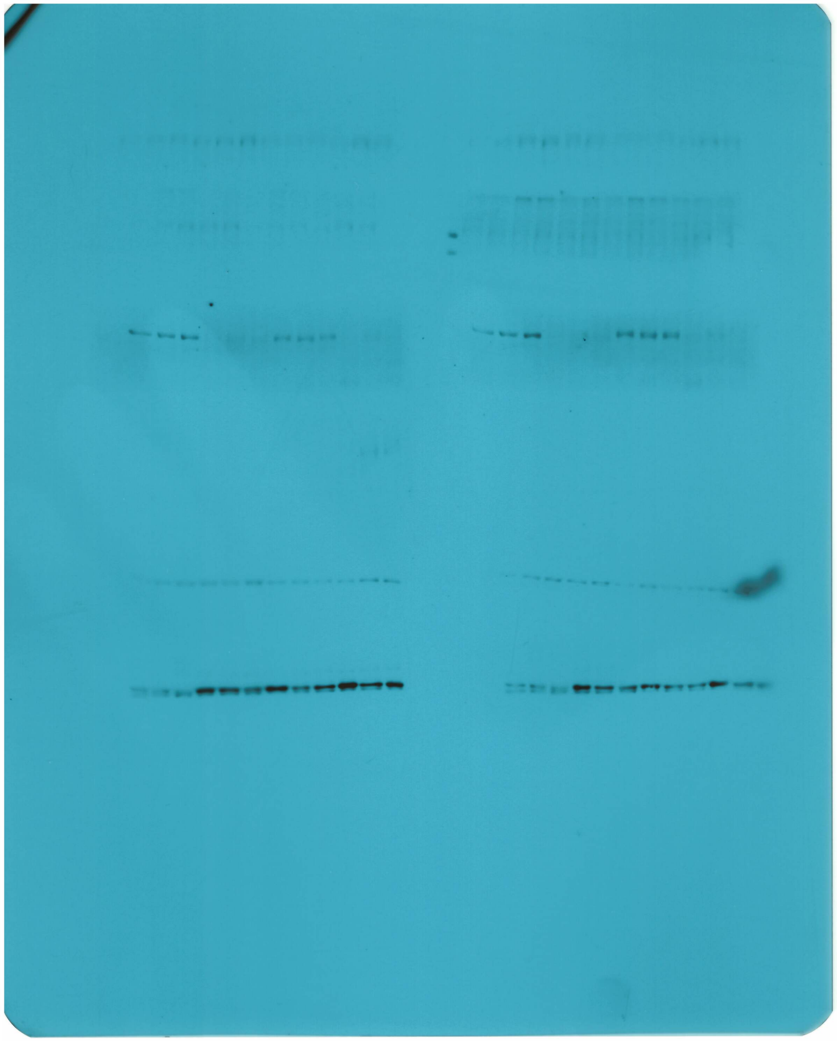

Fig 7D

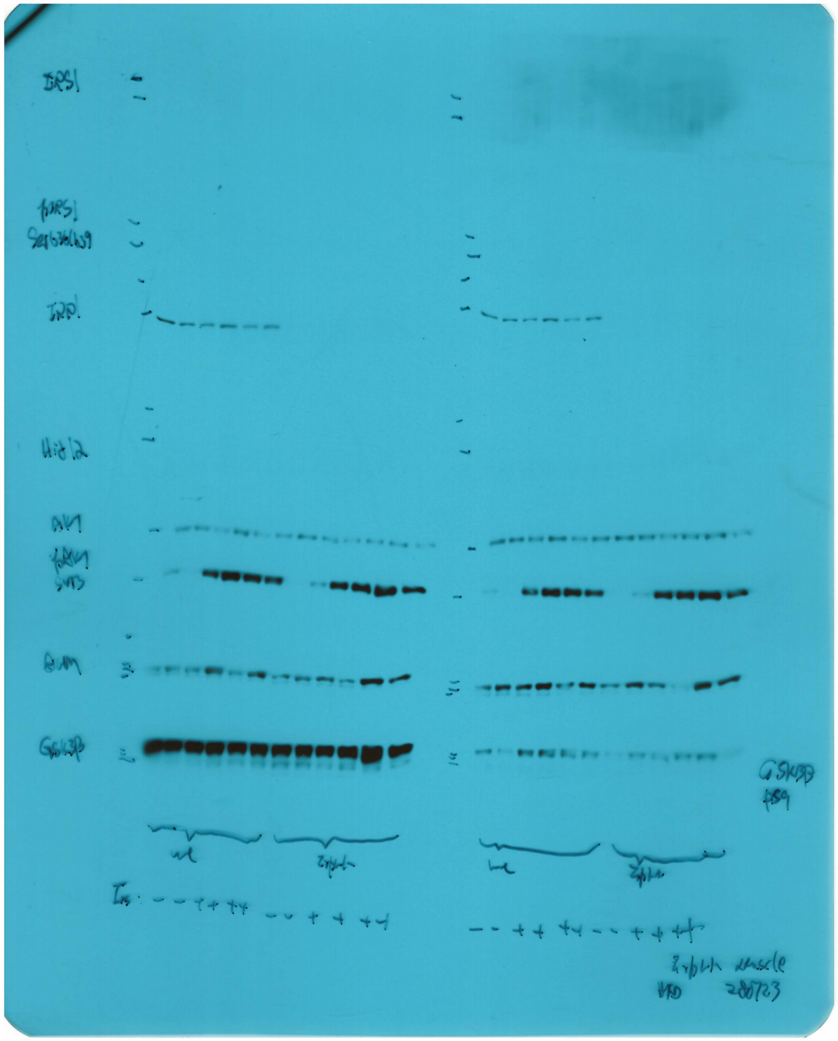

pAKT

Fig 7D

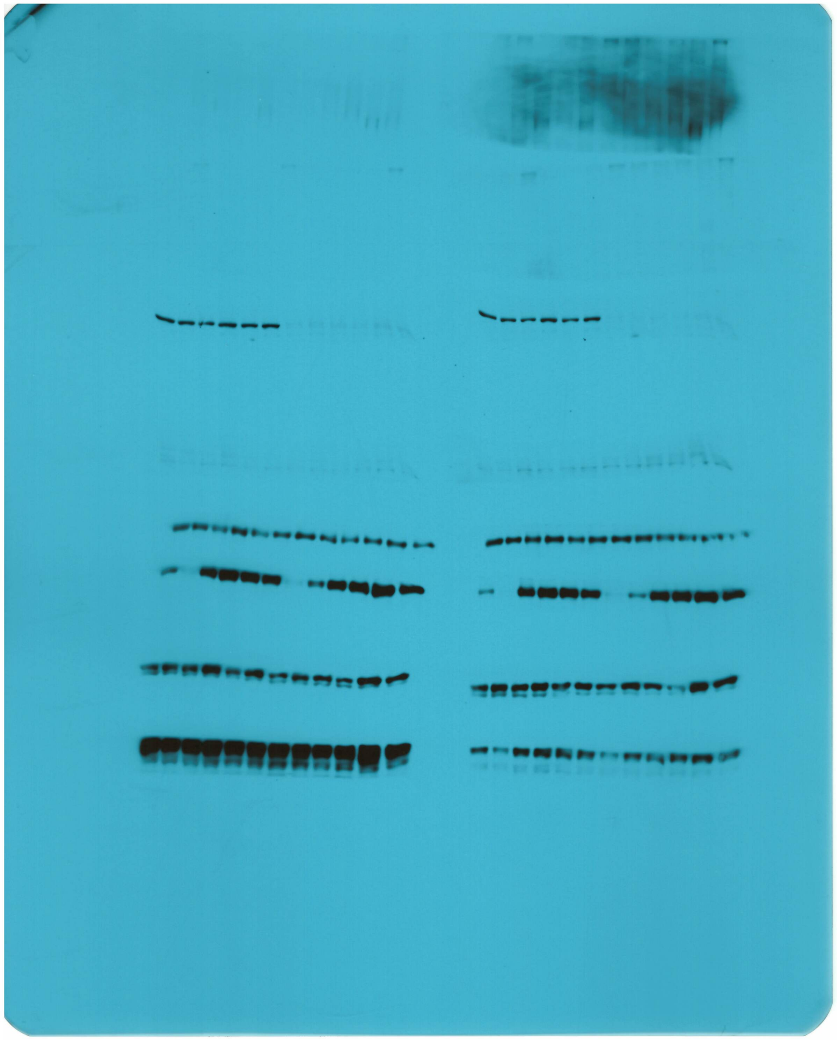

Fig 7D

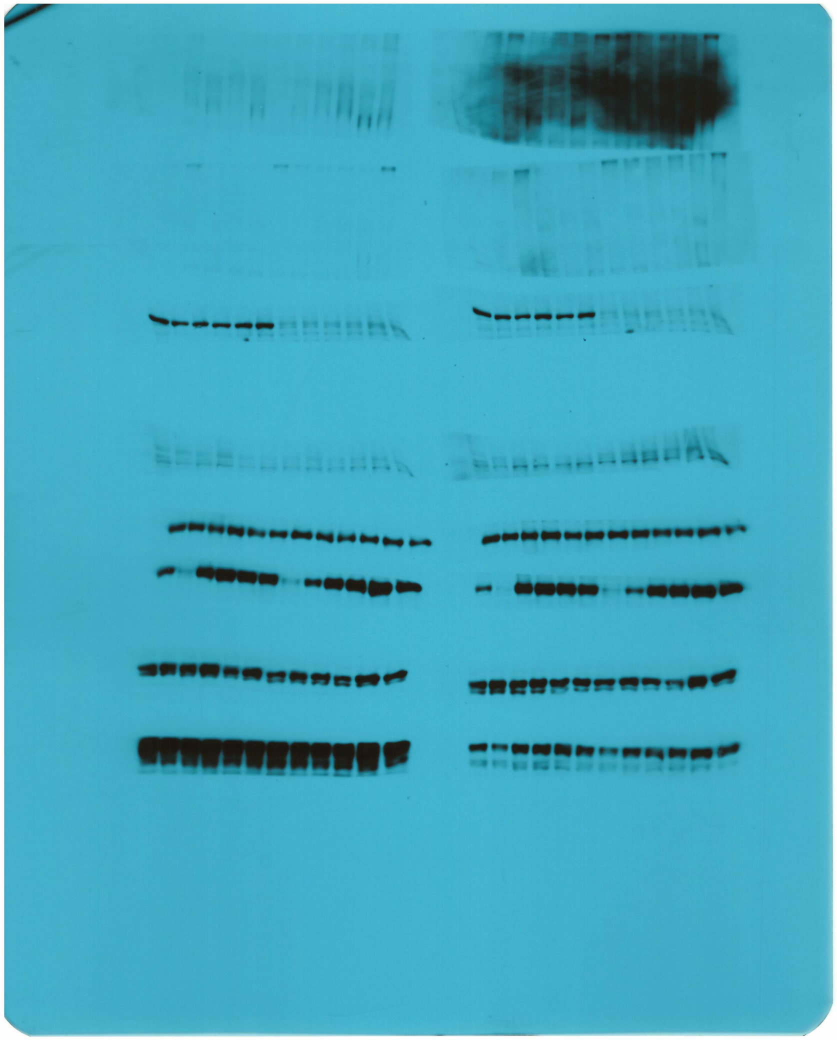

Actin

Fig S9G

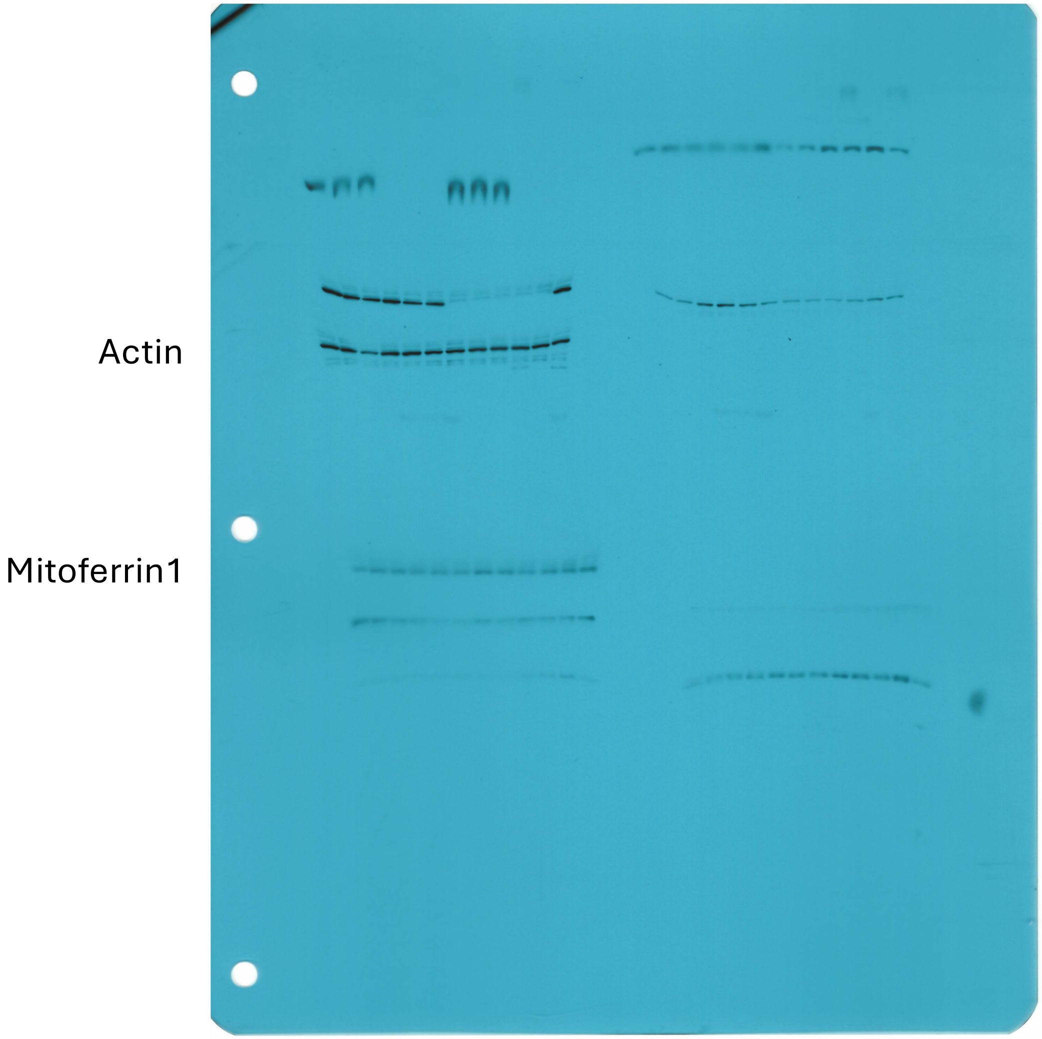

Fig S9G

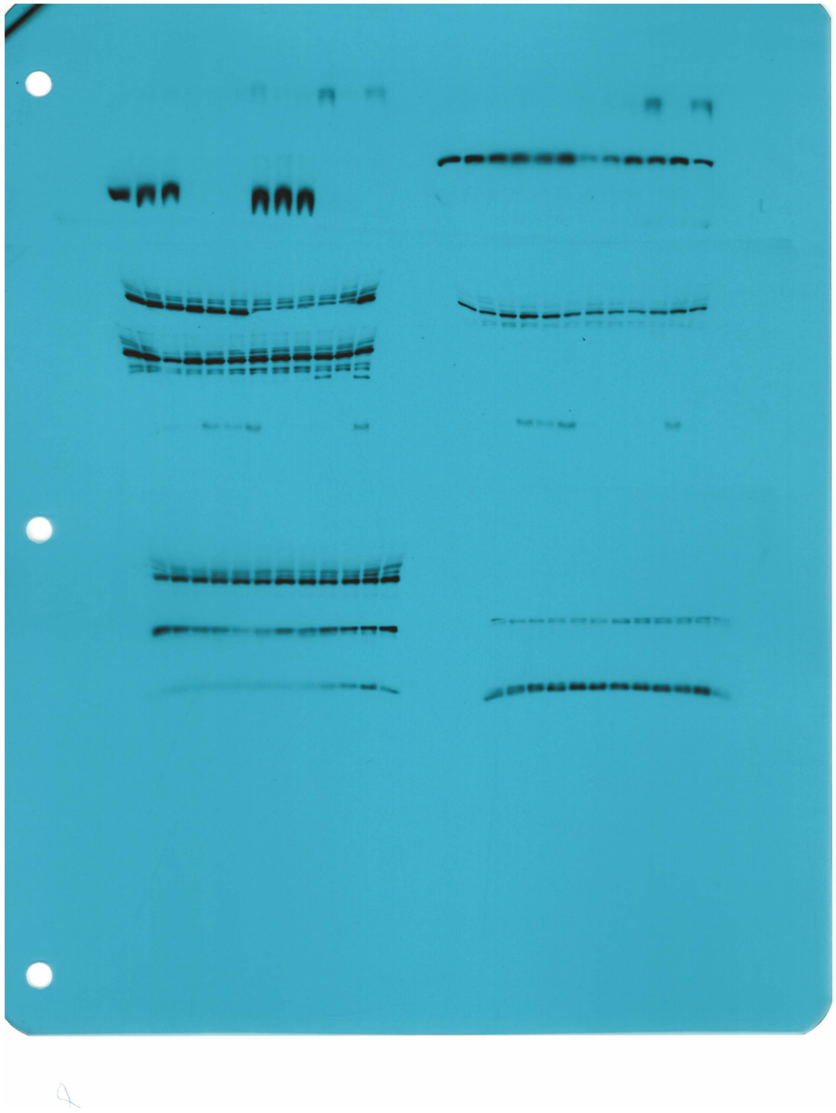

frataxin

Fig S9G

ISCU

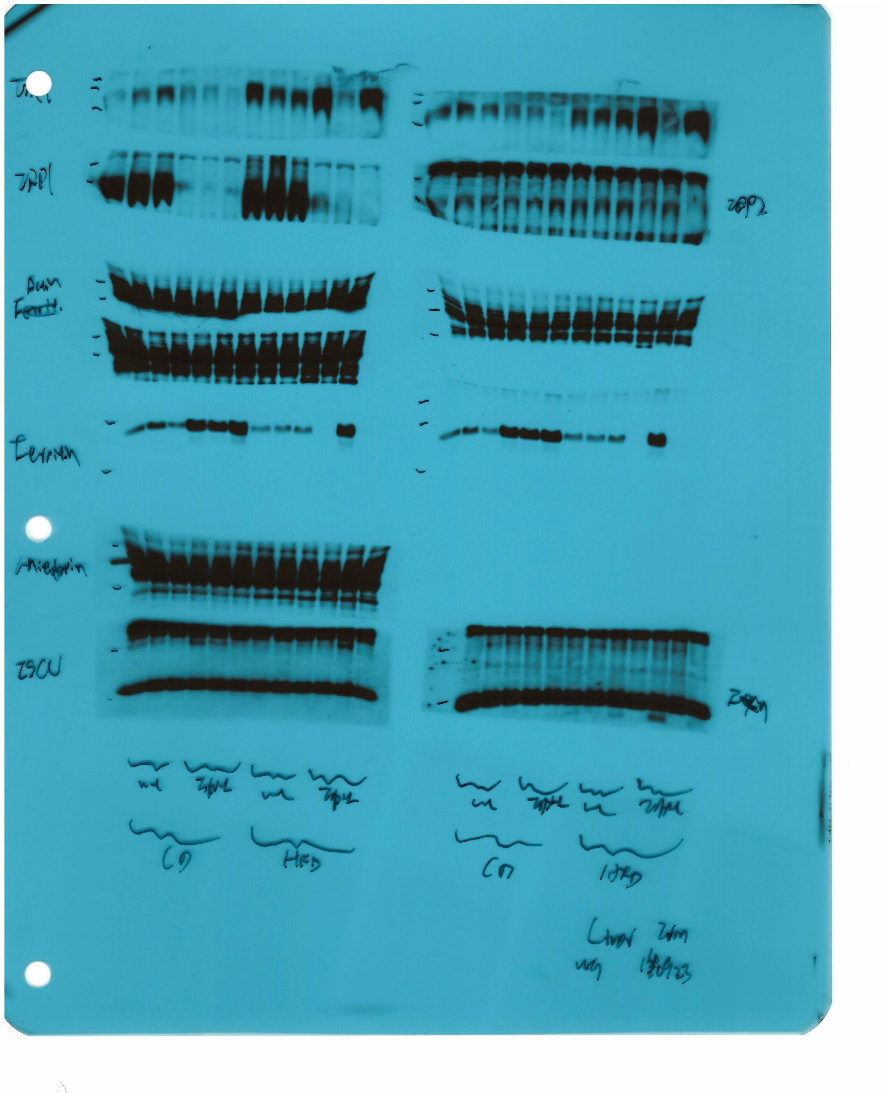

Fig S9H

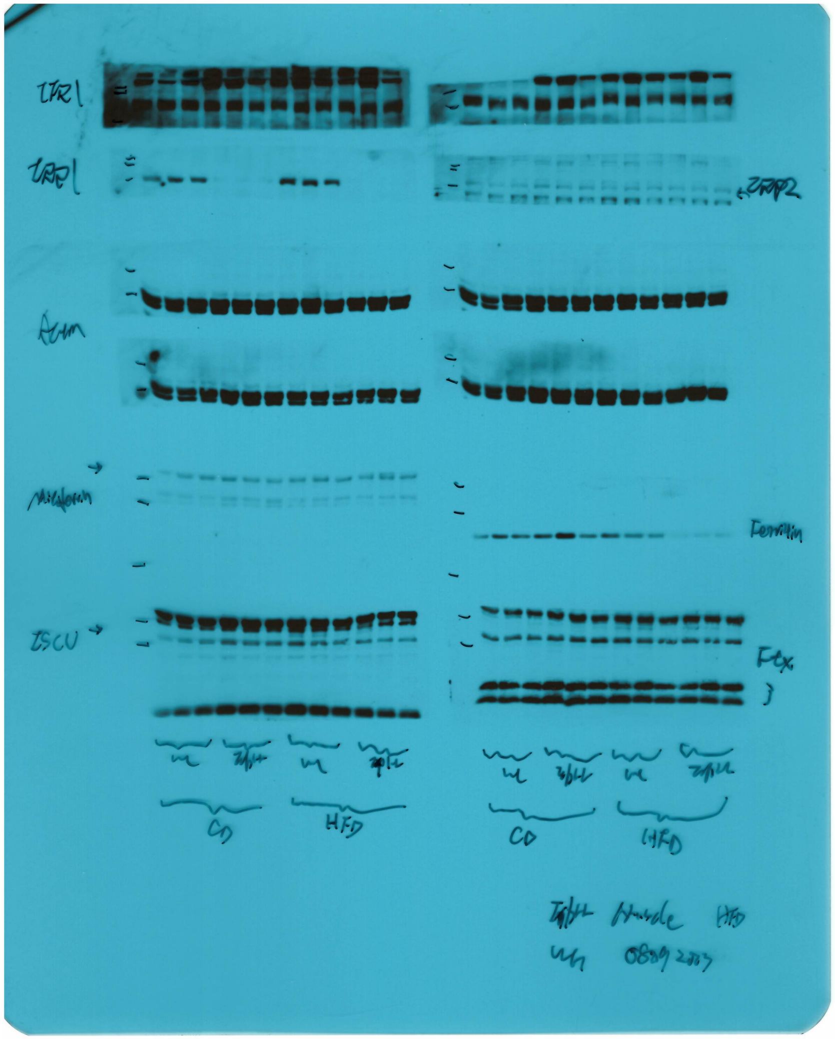

Fig S9H

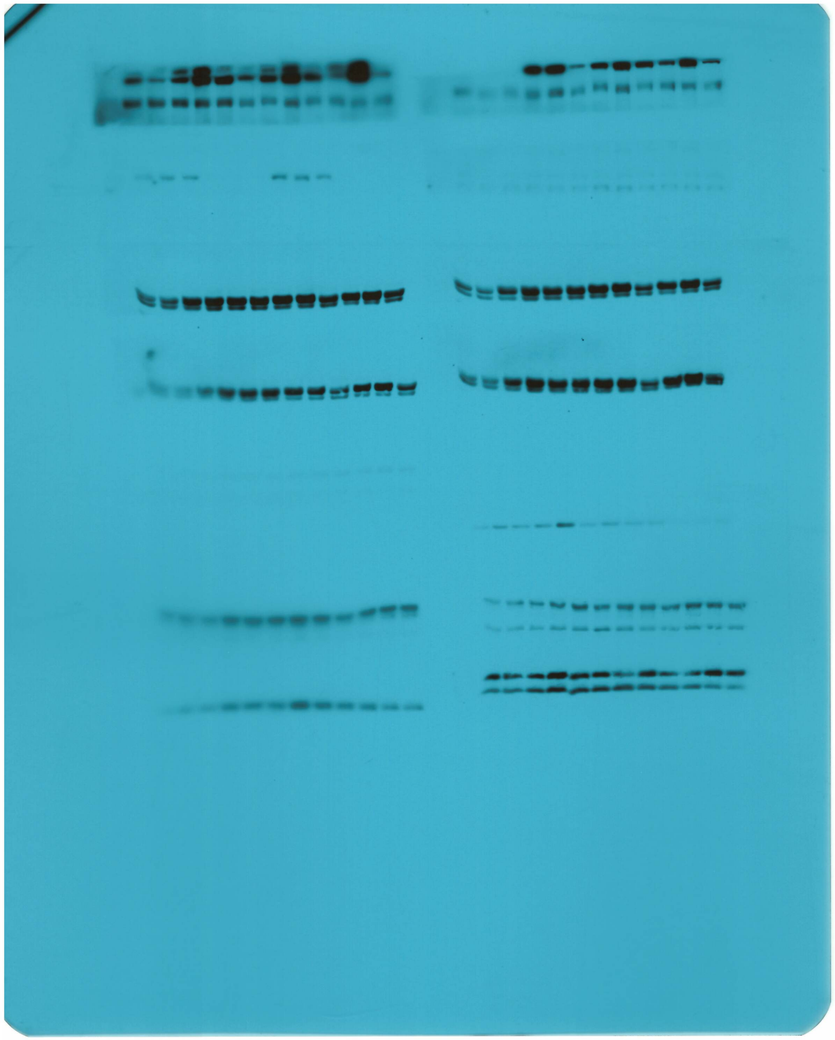

frataxin

Fig S9H

actin

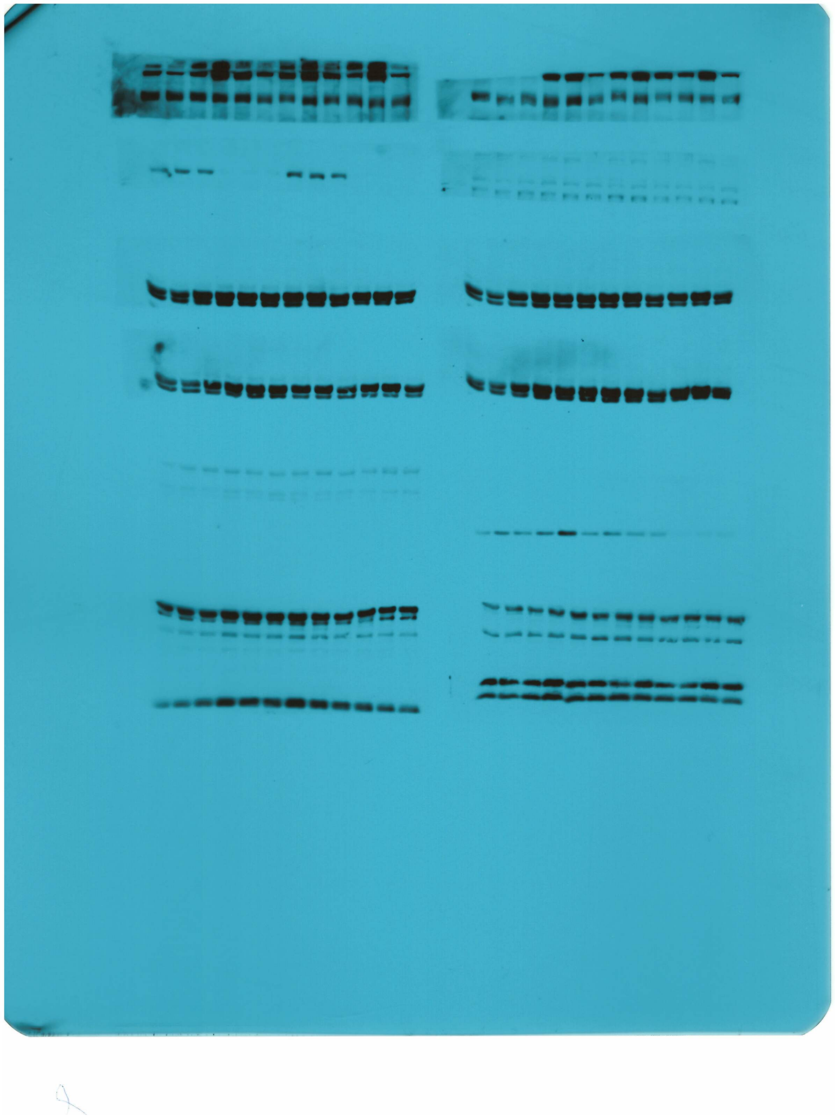

Supplement: Unedited blot and gel images [file jciinsight-11-183247-s115.pdf]
